# Polarity profiling of porous architectures: solvatochromic dye encapsulation in metal–organic frameworks†

**DOI:** 10.1039/d4tc01401d

**Published:** 2024-05-28

**Authors:** Heidi A. Schwartz, Murat Atar, Matthias Spilles, Michael Fill, Manuel Ott, Felix R. S. Purtscher, Josef M. Gallmetzer, Baris Öcal, Selina Olthof, Axel Griesbeck, Klaus Meerholz, Thomas S. Hofer, Uwe Ruschewitz

**Affiliations:** a Department of Chemistry, University of Cologne Greinstraße 4-6 D-50939 Cologne Germany uwe.ruschewitz@uni-koeln.de; b Institute of General, Inorganic and Theoretical Chemistry, University of Innsbruck Innrain 80-82 A-6020 Innsbruck Austria heidi.schwartz@uibk.ac.at

## Abstract

Metal–organic frameworks (MOFs) have gathered significant interest due to their tunable porosity leading to diverse potential applications. In this study, we investigate the incorporation of the fluorosolvatochromic dye 2-butyl-5,6-dimethoxyisoindoline-1,3-dione (=Phth) into various MOF structures as a means to assess the polarity of these porous materials. As a purely inorganic compound, zeolite Y was tested for comparison. The fluorosolvatochromic behavior of Phth, which manifests as changes in its emission spectra in response to solvent polarity, provides a sensitive probe for characterizing the local environment within the MOF pores. Through systematic variation of the MOF frameworks, we demonstrate the feasibility of using (fluoro-)solvatochromic dyes as probes for assessing the polarity gradients within MOF structures. Additionally, the fluorosolvatochromic response was studied as a function of loading amount. Our findings not only offer insights into the interplay between MOF architecture and guest molecule interactions but also present a promising approach for the rational design and classification of porous materials based on their polarity properties.

## Abstract 2

Polarity determination, analogously to solvent-polarity, of various solid porous materials was performed by utilizing a solvatochromic phthalimide as sensor dye.

## Graphical abstract

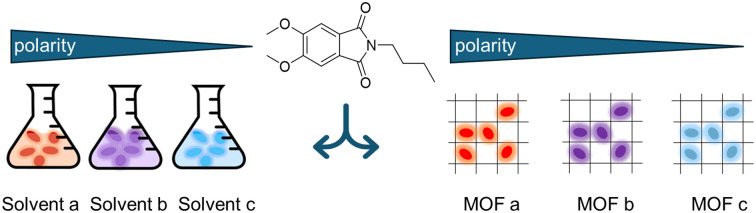

## Introduction

Within the last two decades, interest in metal–organic frameworks (denoted as MOFs in the following) rose rapidly, as these porous compounds were found to have the potential to be applied in various technologically relevant areas. MOFs consist of metal nodes, which are connected *via* organic linkers to form networks with potential voids.^1^ One of their potential applications is their ability to host guest molecules within these voids. In this respect, photoswitchable molecules have been considered, as due to their spatial separation within the MOF's voids photoswitching is improved or even enabled in the solid state: various switch@MOF systems (= the “@” denotes the incorporation of a switch as a non-covalently attached guest molecule within the MOF pore) have been synthesized so far, starting with the work of Fujita and co-workers in 2010 on the *E*/*Z* isomerization of stilbene.^2^ Ever since, many reports followed and showed impressively the enormous potential of MOFs to function as nano-vessels enabling structural transformations of non-covalently attached guest molecules, and thus, introducing new functionalities.^3–16^ Among the many photochromic dyes applied, spiropyrans and spirooxazines are of specific interest, as they do not only exhibit photochromic but also solvatochromic behavior. Solvatochromism describes the dependence of the absorption maximum of a molecule on the surrounding environment.^17^ By the embedment of spiropyrans and spirooxazines into MOFs, our group was able to achieve both photochromism and solvatochromism in the solid state.^7,8,10^ The MOF dependent absorption properties of these spiro-compounds allowed for a very basic qualitative polarity classification of the respective MOF hosts.^7,8,10^ The dependence of the emission maximum of an embedded chromophore on the surrounding MOF environment was already reported in 2017 by the Shustova group,^18^ but without connecting this shift of the emission maximum to the chemical nature of the MOF voids. In later work, it was attempted to relate this behavior to the different sizes of the MOF pores.^19,20^ Remarkably, Hirai *et al.* showed that the relocation of a TICT fluorophore (TICT: twisting intramolecular charge transfer) encapsulated in a MOF pore from a polar to a nonpolar environment induced by a UV laser led to a drastic change of the emission properties of the fluorophor.^21^

**Fig. 1:**
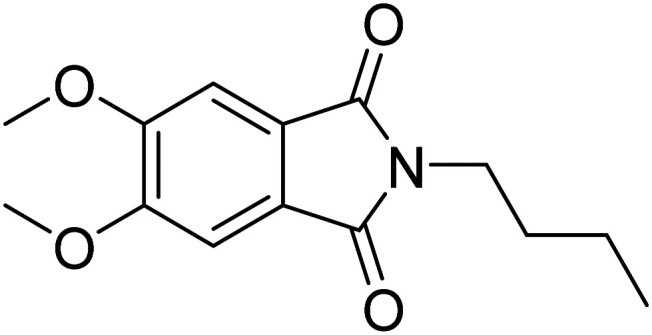
Structure of 2-butyl-5,6-dimethoxyisoindoline-1,3-dione (=Phth). The molecule size is ∼6.2 Å × 12 Å, and measures 13 Å in its diagonal. Sizes were estimated utilizing the VMD (visual molecular dynamics) package.^29^

To investigate the impact of different MOF pores on the emission properties of encapsulated fluorophores in more detail, it is desirable to have a probe molecule with a smaller size at hand compared to the molecules applied in the studies summarized above. This would allow to include MOFs with smaller entrance windows and pores in these investigations. Even more, smaller molecules are expected to show a higher mobility within larger MOF pores thus reducing the effect of specific orientations of the fluorophore due to spatial restrictions on its emission properties. Notably, smaller molecules should also increase the probability of pore filling with more than one guest molecule so that next to host–guest interactions, guest–guest interactions must also be considered. For this reason, we have chosen a small and polarity sensitive phthalimide dye to be investigated as a probe molecule for the polarity within various MOF pores. These phthalimide dyes typically exhibit shifted emission bands upon varying polarity of the surrounding medium (solvent), which is referred to as fluorosolvatochromism^22^ or solvatofluorochromism.^23^ A very well studied example of such a strong solvatochromic fluorophore with a change in dipole moment with *μ*_e_ > *μ*_g_ upon excitation is 4-aminophthalimide with a Stokes shift of Δ*ν̃* = 5010 cm^−1^ for diethyl ether → water.^24–27^ In the present work, the structurally related compound 2-butyl-5,6-dimethoxyisoindoline-1,3-dione^28^ (=Phth) was chosen as guest molecule to confirm the concept of MOFs as “solid solvents” for dye molecules^10^ and to probe the polarity within MOF pores. This phthalimide was selected as it shows an even higher Stokes shift of Δ*ν̃* = 6547 cm^−1^ (diethyl ether → water),^28^ and is therefore expected to respond more sensitive to changes in polarity of the surrounding medium. The structure of Phth is shown in Fig. 1.

When dissolved in solvents of varying polarity, the emission maxima are progressively red-shifted by increasing the solvent polarity (see Fig. S1 and Table S1, ESI†). According to the elutrope series of Snyder,^30^ methanol is the most polar of the used solvents, followed by ethanol, chloroform, acetone and finally toluene. The red-shift between the less polar toluene (*λ*_em_ = 431 nm) and the most polar methanol solution (*λ*_em_ = 490.5 nm) is significant. In this work, the optical response of Phth encapsulated in different MOF host matrices as well as zeolite Y is studied. These findings are compared to the results of the formerly reported systems containing a nitro-substituted spiropyran^10^ and a spirooxazine.^8^ In order to address the influence of varying pores sizes, pore shapes and building blocks, the following MOFs were selected: MOF-5,^31^ MIL-68(In) and MIL-68(Ga),^32^ MIL-53(Al),^33^ MFM-300(Ga_2_),^34^ UoC-2(Ga,1F),^35^ UoC-2(Ga,2F),^35^ UiO-66^36^ and ZIF-8.^37^ The four first mentioned MOFs are built up with terephthalate linkers but differ in the respective metal cations and the sizes of the pores. MFM-300(Ga_2_), UoC-2(Ga,1F) and UoC-2(Ga,2F) are isostructural with the MFM-300 topology and contain the same (Ga^3+^) cation, but the incorporated bptc linker (biphenyl-3,3′,5,5′ tetracarboxylate) shows an increasing degree of fluorination. With UiO-66 and ZIF-8, two MOFs with comparably small pore sizes were added, which are expected to lead to strong host–guest interactions. Furthermore, UiO-66 with a Zr_6_O_4_(OH)_4_ core as well as ZIF-8, the only MOF not based on carboxylate linkers in this investigation, show a significantly different structural composition compared to the other MOFs in this study. Finally, as a different type of a porous material, zeolite Y^38^ was selected to elucidate the impact of an anionic, purely inorganic cage on the optical properties of the embedded dye. In Table 1, the important characteristics of these host matrices are summarized, including the respective metal-nodes, organic linkers as well as pore sizes (taking the van der Waals radii into account). Additionally, the individual MOF structures are shown. Notably, MIL-68(In) and MIL-68(Ga) as well as MFM-300(Ga_2_) and its fluorinated derivatives UoC-2(Ga,1F) and UoC-2(Ga,2F) exhibit analogous structural motifs, so only one MOF structure is depicted.

**Table 1:** Individual characteristics of the applied porous host materials including organic linkers, metal-nodes and the pore size (diameter) as well as the size of the pore aperture (bdc = benzenedicarboxylate; bptc = biphenyltetracarboxylate; mim = methylimidazolate). Pore diameters are given for the solvent-free MOFs (taking the van der Waals radii into account). Additionally, the individual MOF structural motifs are shown. The structural motifs were visualized *via* Diamond 4.4^39^ and the VMD program package^29^

Host matrix	Linker	Metal cation	Pore size (diameter)	Size of the pore aperture	MOF structure
MOF-5^31,40^	bdc	Zn^2+^	∼12 Å	∼8 Å	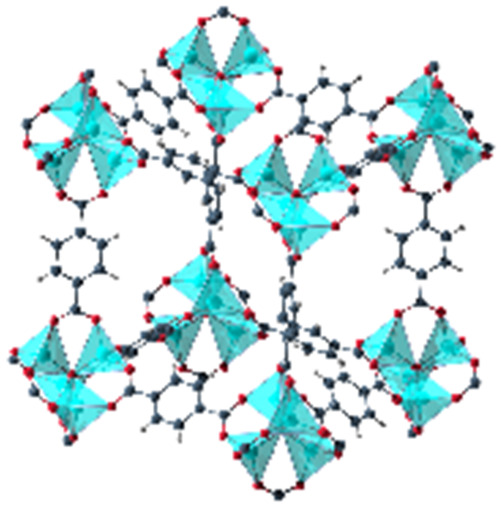
MIL-68(In)^32^	bdc	In^3+^	∼18 Å	∼18 Å	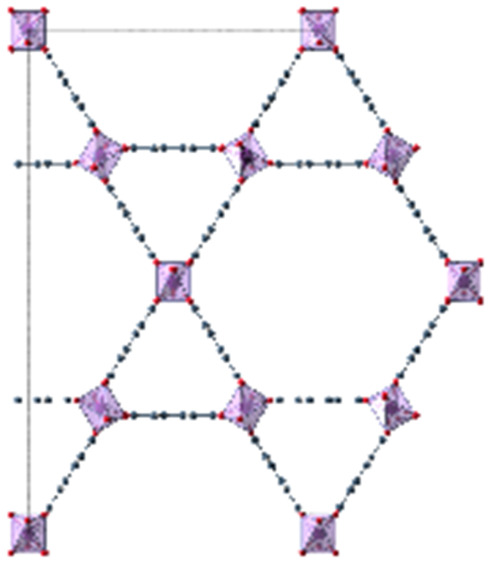
			Hexagonal pore	Hexagonal pore	
MIL-68(Ga)^32^	bdc	Ga^3+^	∼17 Å	∼17 Å	
			Hexagonal pore	Hexagonal pore	
MIL-53(Al)^33^	bdc	Al^3+^	∼12.3 Å x 7.8 Å	∼12.3 Å x 7.8 Å	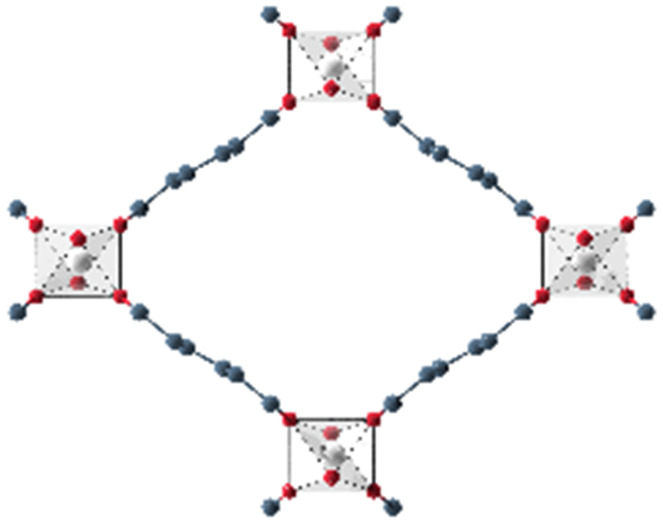
MFM-300(Ga_2_)^34^	bptc	Ga^3+^	6.7 Å	6.7 Å	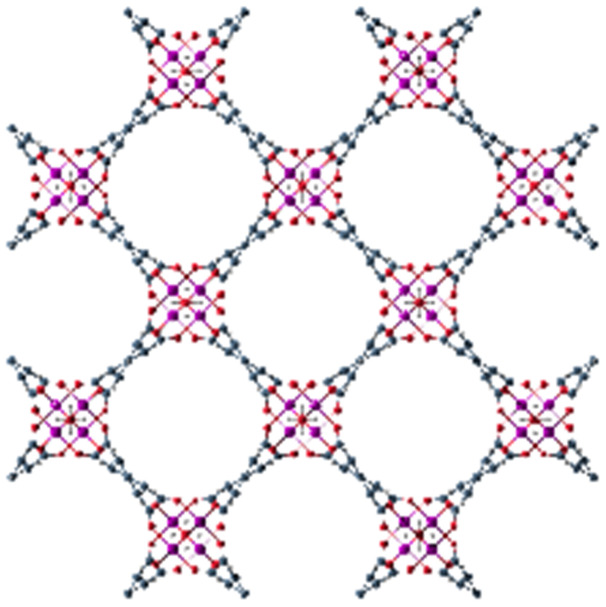
UoC-2(Ga,1F)^35^	4-1F-bptc	Ga^3+^	∼6.5 Å	∼6.5 Å	
UoC-2(Ga,2F)^35^	4,4′-2F-bptc	Ga^3+^	∼6.5 Å	∼6.5 Å	
UiO-66^36,41^	bdc	Zr^4+^	7.5 Å and 12 Å	6 Å	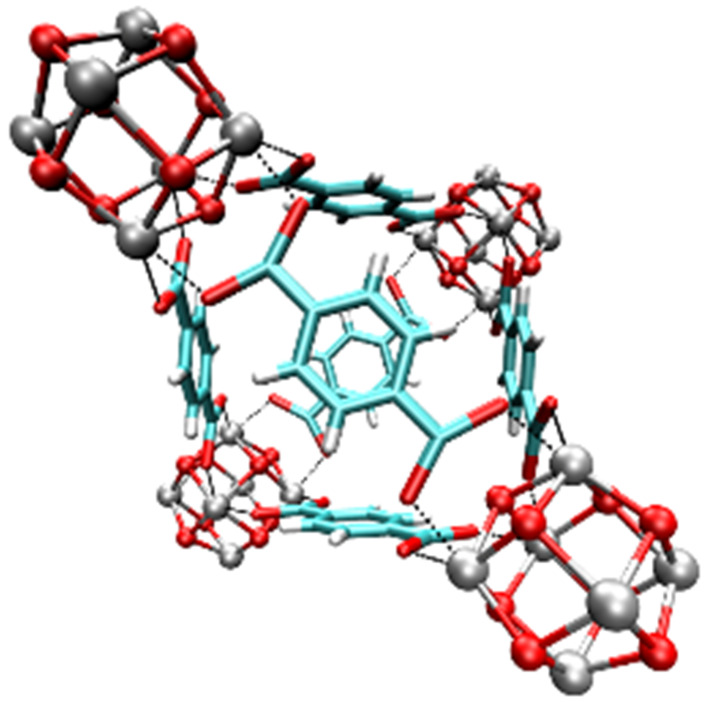
ZIF-8^37,42^	mim	Zn^2+^	11.6 Å	3.4 Å	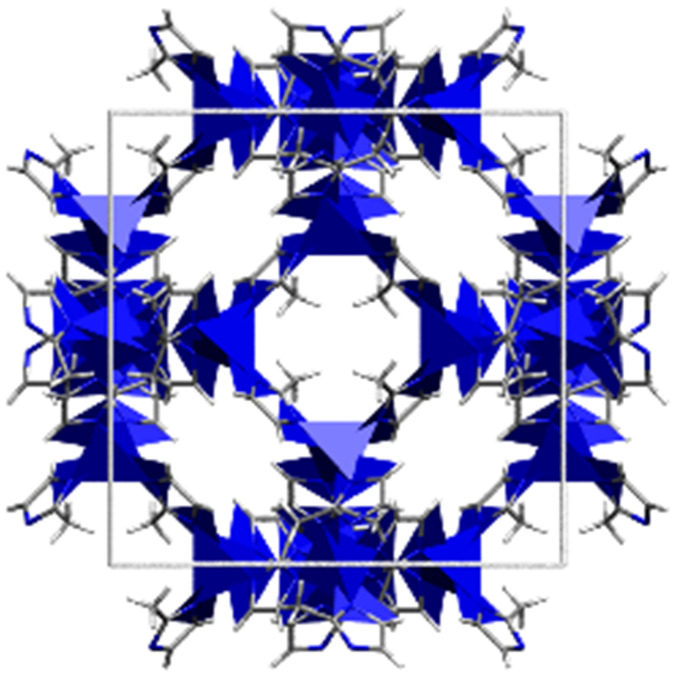
Zeolite Y^38,43^	Sodalith cages built up from edge sharing SiO_4_ and AlO_4_ tetrahedra		12 Å	7.4 Å	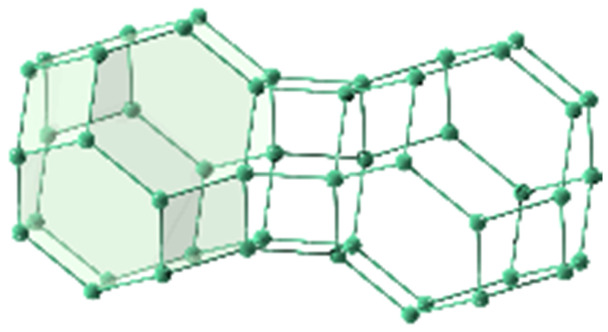

In addition to these investigations, a dilution series of Phth inside MOF-5 and MIL-68(Ga) was studied to address the influence of guest–guest interactions on the optical properties of the embedded Phth dye. These two MOFs were selected, as they exhibit a different polarity, as determined in our previous studies on spiropyrans^10^ and spiroxazines.^8^ Furthermore, alongside the experimental investigations, molecular dynamics (MD) simulations have been performed. In particular, density functional tight binding (DFTB) approaches provide a versatile and effective framework to achieve an accurate description of pristine MOFs as well as guest@host systems.^44,45^ In combination, DFTB MD simulations can achieve comparatively long simulations of guest@MOF systems up to the nanosecond range,^46,47^ providing detailed insight into the underlying host–guest interactions at the molecular level. Thus, in this work, a series of DFTB MD simulations of Phth in the topologically different host systems MOF-5 and MIL68(Ga) has been carried out, thereby considering different degrees of loading.

## Results

Formation of the hybrid systems containing Phth as guest molecules and various MOFs as well as zeolite Y as host materials was achieved by the gas-phase loading approach, which is advantageous, as no effects from solvent molecules have to be considered. This is an essential prerequisite to exclusively study the influence of the porous host on the electronic structure and thus optical properties of the inserted dye molecule. Using this approach, ten new compounds were synthesized:

Phth@MOF-5 (1), Phth@MIL-68(In) (2), Phth@MIL-68(Ga) (3), Phth@MIL-53(Al) (4), Phth@MFM-300(Ga_2_) (5), Phth@UoC-2(Ga,1F) (6), Phth@ UoC-2(Ga,2F) (7), Phth@UiO-66 (8), Phth@ZIF-8 (9) and Phth@zeolite Y (10).

The successful formation of the hybrid systems was confirmed by powder X-ray diffraction (= PXRD) measurements. Here, modulations in intensity of the MOF reflections compared to the non-loaded MOF occur, as the embedded Phth guests change the electron density within the MOF pores and thus modulate the structure factors. The respective diffraction patterns can be found in Fig. S2–S11, ESI.† The composition Phth:MOF, *i.e.* the loading of the MOF pores, was determined utilizing X-ray photoelectron spectroscopy (=XPS). Here, the characteristic core level peaks of both the guest and the host were analysed and their relative intensities were compared to obtain the guest-to-host ratios. The fitted XPS spectra as well as the calculated ratios can be found in the ESI,† Fig. S13–S24 and Tables S3–S7.

### Optical properties of Phth as a function of local environment

**Fig. 2:**
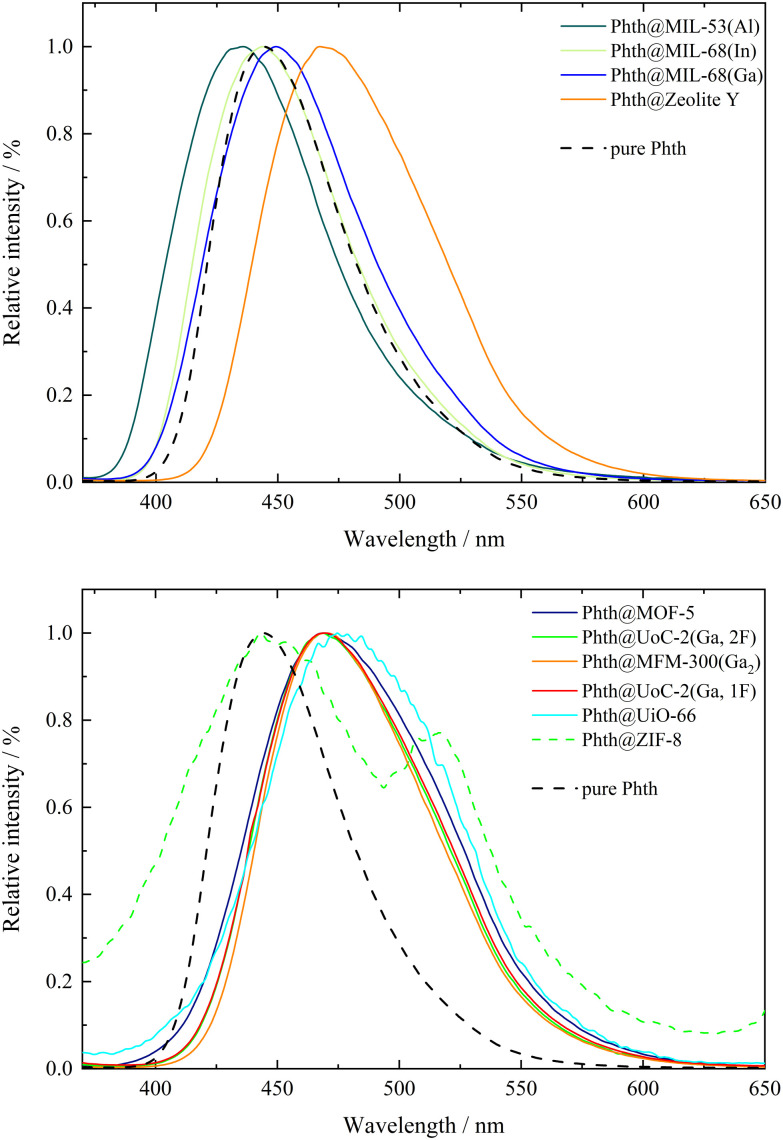
Normalized emission bands of Phth embedded in different porous host matrices in comparison to pure Phth (dashed black lines). Excitation wavelength: *λ*_ex_ = 347 nm (pure Phth and 1 to 8) and *λ*_ex_ = 340 nm (9 and 10).

Phthalimides feature a high sensitivity of their emission bands on the polarity of the solvent, which results in a profound red-shift of the emission bands with increasing polarity of the solvent. According to the previously reported results on the solvatochromism of spiropyrans and spirooxazines inside different MOF hosts,^7,8,10^ host dependent emission maxima are expected for Phth within the different porous matrices as well. The emission spectra of all compounds 1 to 10 are summarized in Fig. 2; for better clarity, the spectra were separated into two figures (top: 2–4, 10; bottom: 1, 5–9). Additionally, the emission spectrum of pristine solid Phth was recorded (dashed black line) to compare the shift of its emission band with those of compounds 1 to 10. The emission bands of the Phth@PM compounds (PM: porous material) are presented as solid lines in different colors. Phth@ZIF-8 (dashed green line, Fig. 2, bottom) features an additional linker-based emission band at *λ*_em_ = 443.5 nm (*λ*_em_ = 449 nm for unloaded ZIF-8).^48^ Additionally, the luminescence characteristics of the pristine host materials were determined to exclude self-emission or excitation of the host instead of the Phth molecule under the given conditions. The respective spectra can be found in Fig. S25–S34, ESI.† In the following, the emission characteristics of Phth inside the various host matrices will be described, starting with Fig. 2, top.

Overall, significant differences in the emission maxima of Phth inside the various host matrices are obvious. When embedded inside MIL-53(Al) (dark green line, Fig. 2, top), an emission maximum at 435.5 nm is found, followed by the slightly red-shifted emission maxima of MIL-68(In) (light green line, respectively, Fig. 2, top) and MIL-68(Ga) (blue line, Fig. 2, top). With emission maxima in the range of 467–470 nm, the host matrices zeolite Y (orange line, Fig. 2 top) as well as MOF-5 (purple line, Fig. 2, bottom), UoC-2(Ga,2F) (green line, Fig. 2, bottom), MFM-300(Ga_2_) (orange line, Fig. 2, bottom), UoC-2(Ga,1F) (red line, Fig. 2, bottom) and UiO-66 (turquoise line, Fig. 2, bottom) show a significantly higher red-shift. For ZIF-8 (dashed green line, Fig. 2, bottom), two emission maxima are present, of which one is positioned at 443.5 nm (self-emission of ZIF-8, *vide supra*)^48^ and one at 516 nm originating from the embedded Phth (see Fig. 2, bottom, green dashed line). The shifted self-emission of ZIF-8 (shift from 449 nm^48^ to 443.5 nm) originates from the interactions of the linker with the Phth guest. The second emission maximum is considerably bathochromically shifted in comparison to the other host matrices of this study. For a better overview, the fitted values of the emission maxima *λ*_em_ of 1 to 10 are listed in Table 2.

**Table 2:** Emission maxima of Phth embedded in different porous host materials, Phth@PM

Host matrix	*λ*_em_(Phth) in nm
MIL-53(Al) (4)	435.5
MIL-68(In) (2)	444
MIL-68(Ga) (3)	449.5
zeolite Y (10)	467.5
MOF-5 (1)	468.5
UoC-2(Ga,2F) (7)	468.5
MFM-300(Ga_2_) (5)	469.5
UoC-2(Ga,1F) (6)	469.5
UiO-66 (8)	474.5
ZIF-8 (9)	516

In previous studies, solvatochromic spiropyran^10^ and spirooxazine^8^ could also serve as polarity probes for a few selected MOFs. In both studies, we found that the polarity increases from MIL-53(Al) to the MIL-68 family and, finally, to MOF-5 being fully consistent with this current study using Phth as sensor dye. As Phth is small compared to the previously studied spiropyran and spirooxazine molecules, we were able to investigate a larger variety of potential host materials including those with smaller pores and opening windows. *E.g.*, the emission spectrum of Phth@zeolite Y shows that this purely inorganic host has a polarity very similar to that of MOF-5.

Considering only MOFs with unsubstituted carboxylate linkers, a polarity trend MIL-53(Al) < MIL-68(In) < MIL-68(Ga) < MOF-5 < MFM-300 < UiO-66 is found. Tracing this trend back to structural features is not straightforward for all MOFs. The first four MOFs are built from different metal nodes and terephthalate linkers. The metal core of MOF-5 has a significantly higher charge ([Zn_4_O]^6+^) and thus the higher polarity compared to the MIL series is plausible, as the latter contain M^3+^ cations in an octahedral MO_6_ coordination with *trans*-hydroxy anions bridging them into chain-like structural units. For the smaller Ga^3+^ compared to larger In^3+^ cation, a higher ionicity and thus a higher polarity is not surprising. However, the low polarity of MIL-53(Al) is unexpected. An influence of the MOF topology (shielded metal centers) or the sizes of the MOF pores (cp. the work by the Shustova group)^19,20^ on their polarity cannot be excluded. MFM-300 also consists of Ga^3+^ cations in an octahedral GaO_6_ coordination. In contrast to the MOFs of the MIL family, hydroxy groups in *cis* positions connect the octahedra to zig-zag chains. This and the highly charged bptc linker with four carboxylate groups are assumed to be the reasons for the higher polarity of this MOF. Furthermore, the pore size of this MOF is significantly smaller (see Table 1) than those of the aforementioned MOFs. This could lead to geometrical stress on the embedded Phth (tilting and twisting of the inserted dye) and therefore also to a shift in the emission spectrum. The highest polarity in the series above is found for UiO-66, which is also built from terephthalate linkers, but contains a metal core with the highest charge of all compounds in this investigation, namely [Zr_6_O_4_(OH)_4_]^12+^. Analogous to MFM-300, UiO-66 also exhibits a small pore, which further contributes to the shift in the emission spectrum as a result of geometrical stress (*vide supra*).

It is surprising that fluoro-substituents of the bptc linker in UoC-2(Ga,1F) and UoC-2(Ga,2F) have only a negligible impact on their polarity, although it was shown that such fluorination of linkers has a significant influence on their gas sorption properties.^49,50^ However, a close inspection of the crystal structures of both UoC-2 derivatives shows^35^ that the fluorine atoms do not protrude into the pore interior, as they lie in the plane of the aromatic rings and are thus part of the channel “walls”.^35^ Furthermore, *ab initio* DFT calculations show that the influence of a monofluorination of a benzene ring on its electric (dipole/quadrupole) moments is relatively small and only increases significantly with a higher degree of fluorination.^51^ Therefore, it is plausible that the polarity of the MFM-300 type structures is not significantly affected by the introduction of fluoro-substituents. The high polarity of ZIF-8 may sound surprising, but the high density of ZnN_4_ secondary building units (= SBUs) in this zeolitic MOF explains the high measured red-shift (*λ*_em_ = 516 nm) rather straightforward.

**Fig. 3:**
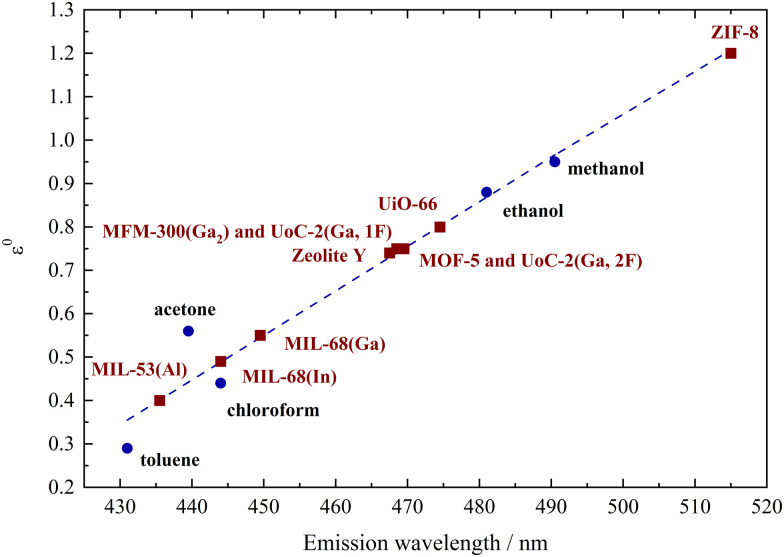
Plot of the emission maxima of Phth dissolved in different solvents (blue dots) *versus* the elution power *ε*^0^ on alumina taken from Snyder^30^ connected by a linear fit (blue dotted line), classifying the polarity of the respective MOFs and porous materials (red squares).

To illustrate the correlation between the polarity of the investigated porous hosts and different solvents, the emission maxima of dissolved Phth were plotted as a function of the elution power *ε*^0^ of the respective solvent on alumina according to Snyder^30^ (see Fig. 3). These values (blue dots) were linearly fitted (blue broken line) and the results of 1 to 10 (Table 1) were added in a way that the measured *λ*_em_ was placed on this blue broken line (red squares).

Within this polarity classification scheme referring to the elution power *ε*^0^ on alumina, MIL-53(Al) is positioned between toluene and acetone, while MIL-68(In) and MIL-68(Ga) show a polarity similar to chloroform. The polarity of MOF-5, zeolite Y, MFM-300(Ga_2_), UoC-2(Ga,1F) and UoC-2(Ga,2F) is located between chloroform and ethanol, while the one of UiO-66 is closer to ethanol. The highest red-shift was found for Phth in ZIF-8 with *λ*_em_ = 516 nm, which is according to Fig. 3 beyond the polarity of methanol.

Basically, there is no exact definition of the term “polarity”, as many different variables contribute to this term, which are referred to as specific and non-specific solute/solvent interactions.^17^ This includes not only the relative permittivities, but also *e.g.*, the dipole moment and molar volume of the respective solvent. Polarity could potentially encompass (a) the permanent dipole moment of a compound, (b) its relative permittivity, or (c) the collective molecular attributes contributing to the interaction forces between solvent and solute molecules.^17^ These considerations are also reflected in the distribution of emission maxima of phthalimide in various solvents as shown in Fig. 3. According to Snyder,^30^ only the relative permittivities are considered here, while the potential formation of hydrogen bonds is neglected. Utilization of the *E*_T_(30) scale according to Reichardt^52,53^ would result in the same polarity ranking. When applying this to a porous host matrix, the nature of both the linker and the metal node naturally plays a significant role. Additionally, the pore size must be considered, as it can exert geometric stress on (fluoro-)solvatochromic molecules under given conditions, thus also contributing to the modulation of their optical properties. Thus, it is not solely the permittivity stemming from the nature of the MOF linkers and the formation of hydrogen bonds, which can be considered as analogous to solute/solvent interactions. As an additional point, also the structure of the cavity contributes to the host/guest interactions. This structural aspect is a MOF specific feature. In the case of UiO-66 and ZIF-8, due to the small pore diameter (see Table 1), it is therefore assumed that geometric stress is induced on the embedded phthalimide, leading to a distortion of the molecule and thus modulation of the emission properties. Thus, it becomes evident that further investigations, taking these structural aspects and interaction sites within the MOF into account, are necessary to convert the qualitative polarity scale, which has now been formulated based on the results with spiropyrans,^10^ spirooxazines,^8^ and phthalimide (this study), into a more quantitative one. A mandatory step towards this quantitative polarity scale can be accomplished *via* MD simulations of MOF–guest interaction strength as well as preferred binding sites (this work within the following section) and *via* structural analysis (solid-state NMR and total X-ray scattering) to understand the occurring deviations from the established polarity scales (cp. acetone *vs.* chloroform for the “better” understood solvent-dependent polarity, Table S1, ESI†).

### Optical properties as a function of guest loading

**Fig. 4:**
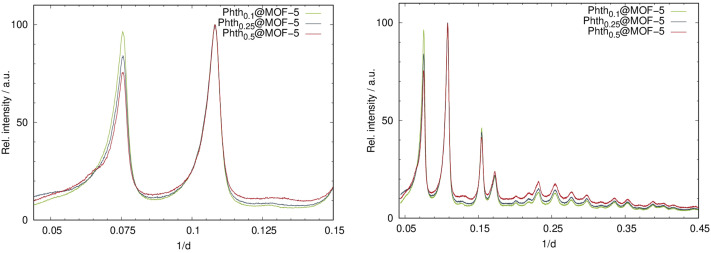
Left: PXRD patterns of the serial dilution of Phth in MOF-5; right: detailed view of the two low angle reflections of Phth_*x*_@MOF-5 (Huber G670: *λ* = 1.54056 Å; 298 K).

In the previous chapter, we have shown that there is a distinct influence of the surrounding host matrix (*i.e.* voids of the porous host) on the optical properties of the encapsulated fluorosolvatochromic Phth dye, which we interpret in comparison to the respective behavior in solvents as a changing polarity of the MOF/zeolite pores. Our assumption is supported by very similar findings on spiropyrans and spirooxazines embedded in MOF hosts.^8,10^ However, one might argue that a solvent provides an isotropic environment, which is certainly not correct for *e.g.*, MOFs consisting of cationic metal cores and, in most cases, anionic aromatic carboxylate linkers. On the other hand, at room temperature a high mobility of the guest molecules within the voids of the porous hosts can be assumed,^47^ which would “simulate” an isotropic surrounding. Furthermore, up to now we have only considered host–guest interactions, which should be correct for low guest loadings. However, for higher loadings guest–guest interactions also need to be taken into account. Therefore, we have explored the influence of guest loading on the optical properties of the encapsulated Phth dye as well. It is postulated that increased loading leads to the occupation of various adsorption sites, thereby prompting a change in optical behavior. Note: in solvents, typically a diluted solution is investigated. Accordingly, within this work three different amounts of Phth loadings within two different MOF hosts were studied with regard to their optical properties. For this, one MOF with a high cubic symmetry (MOF-5) and one with a lower orthorhombic symmetry (MIL-68(Ga)) were selected. For each, three dilutions were synthesized with a starting molar ratio *n*_Phth_ : *n*_MOF_ of (a) 0.125 : 1, (b) 0.25 : 1, and (c) 0.5 : 1, respectively. The incorporation of Phth with increasing amounts was successfully confirmed by means of PXRD measurements. In Fig. 4, the diffraction patterns for the serial dilution of Phth in MOF-5 are presented with the starting *n*_Phth_ amount as index. The ratio of the intensities of the first two (strongest) reflections (Fig. 4, right) changes according to the expected loading indicating the successful incorporation of the Phth dye molecules. Furthermore, no additional peaks are present thus confirming the complete absence of free crystalline Phth.

**Fig. 5:**
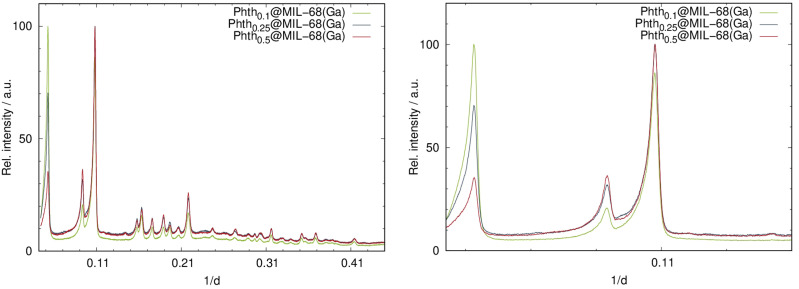
Left: PXRD patterns of the serial dilution of Phth in MIL-68(Ga); right: detailed view of the low angle region of the PXRD patterns of Phth_*x*_@MIL-68(Ga) (Huber G670: *λ* = 1.54056 Å; 298 K).

Analogous to the findings of Phth_*x*_@MOF-5, different loading degrees also result in intensity alterations for Phth_*x*_@MIL-68(Ga) (Fig. 5). Again, the absence of additional reflections in the PXRD patterns is indicative that the sample does not contain free crystalline Phth.

To determine the amount of encapsulated Phth guest molecules quantitatively, XPS measurements were performed. The results are summarized in Table 3. Detailed information on the calculation processes of the guest-to-host ratios from XPS data is given in Tables S6 and S7 (ESI†). Notably, all nitrogen signals were very broad, pointing to a successful incorporation and a high mobility of the Phth dye inside the MOF voids (see Fig. S23 and S24, ESI†).

**Table 3:** Nominal compositions of Phth_*x*_@MOF-5 and Phth_*x*_@MIL-68(Ga) compared to the composition obtained by XPS measurements; results of emission spectra (Fig. 6) are added in the last column

Phth_*x*_@MOF-5	Nominal composition	Composition as obtained *via* XPS^a^	*λ*_em_(Phth) in nm
Phth: Zn_4_O(bdc)_3_	0.1 : 1	0.1 : 1	484.5
Phth: Zn_4_O(bdc)_3_	0.25 : 1	0.2 : 1	473.5
Phth: Zn_4_O(bdc)_3_	0.5 : 1	0.4 : 1	471.5
Phth@MOF-5 (1)		2.4 : 1 (see Table 1 and Table S5, ESI)	468.5

Phth_*x*_@MIL-68(Ga)			
Phth: Ga(OH)(bdc)	0.1 : 1	0.2 : 1	448.5
Phth: Ga(OH)(bdc)	0.25 : 1	0.3 : 1	448.5
Phth: Ga(OH)(bdc)	0.5 : 1	0.3 : 1	450
Phth@MIL-68(Ga) (3)		0.4 : 1 (see Table 1 and Table S5, ESI)	449.5

a Due to the modest precision of the analysis from XPS data, the values for the composition are generally given with only one decimal. Including the second decimal for Phth: Ga(OH)(bdc), the ratios 0.18 : 1, 0.26 : 1, 0.31 : 1, and 0.37 : 1 are calculated representing the expected trend from the nominal composition.

The results of the XPS measurements are in good agreement with the nominal composition of Phth_*x*_@MOF-5. For Phth_*x*_@MIL-68(Ga), significant discrepancies are found for *x* = 0.1 and *x* = 0.5. For Phth_0.1_@MIL-68(Ga), the low signal-to-noise ratio (cp. Fig. S24, top right; ESI†) might be responsible for the discrepancy in the calculated amount of Phth. For the highest loading (*x* = 0.5), it was found in previous experiments (Table S5, ESI†) that only a maximum loading of approx. 0.4 can be reached. The excess of Phth sublimed during the loading process.

**Fig. 6:**
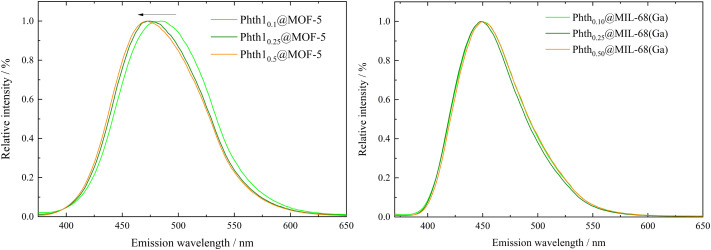
Emission spectra of Phth_*x*_@MOF-5 (left) and Phth_*x*_@MIL-68(Ga) (right) with *λ*_ex_ = 347 nm.

To investigate possible influences of the degree of filling on the emission bands of the embedded Phth, fluorescence spectra of all six compounds Phth_*x*_@MOF were recorded (Fig. 6).

For Phth_*x*_@MIL-68(Ga), no significant trend of *λ*_em_ in dependence of the pore filling is observed (Fig. 6, right and Table 2). A shift from 448.5 nm (*x*_nominal_ = 0.1) to 450 nm (*x*_nominal_ = 0.5) is in the range of the resolution of the spectrometer. Obviously, orientation and position of the Phth guest molecule inside the channels of MIL-68(Ga) do not influence its spectroscopic properties. They are also not influenced by guest–guest interactions, which are to be assumed at higher pore fillings. A different situation is observed, when Phth is embedded in MOF-5. With an increasing amount of embedded Phth, *λ*_em_ is blue-shifted from 484.5 nm (*x*_nominal_ = 0.1) to 471.5 nm (*x*_nominal_ = 0.5) (see Fig. 6, left and Table 2). This could be due to varying orientations of the Phth guest within the cubic pores of MOF-5 depending upon the loading. Therefore, in the following, the positions of one, two and three Phth molecules per MOF pore (of MOF-5 and MIL-68(Ga)) were studied *via* molecular dynamics (MD) simulations.

### MD simulations

**Fig. 7:**
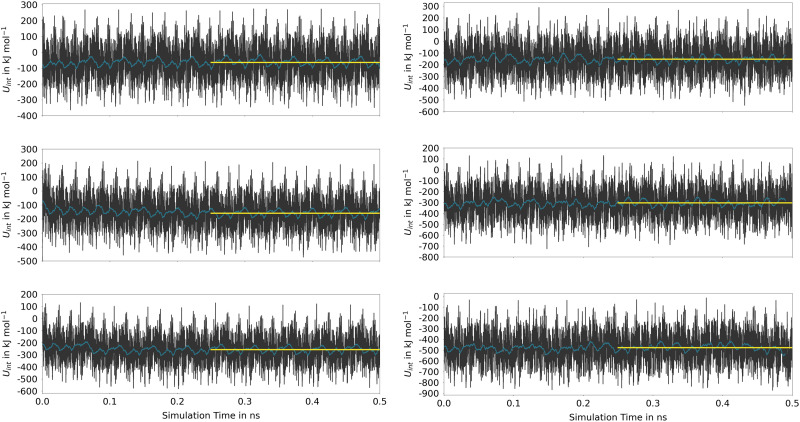
Time evolution of the interaction energy (black) and the associated running average over 2500 data points (blue) obtained for one (top), two (middle) and three guest molecules (bottom) for MOF-5 (left) and MIL-68(Ga) (right) determined *via* SCC DFTB MD simulations. The associated averages (yellow) have been determined using only the last 0.25 ns of the trajectory.

Based on the simulation data, the associated instantaneous interaction energy *U*_Int_ can be determined as*U*_Int_ = *U*_guest@MOF_ − 〈*U*_MOF_〉 − *n*〈*U*_guest_〉with *U*_guest@MOF_ being the instantaneous total energy (*i.e.* kinetic plus potential energy) obtained from the MD simulation of the combined guest@host system, *U*_MOF_ and *U*_guest_ are the corresponding total energies obtained from separate MD simulations of the isolated guest and MOF systems under the same conditions. The angular brackets 〈⋯〉 denote the respective ensemble average and *n* corresponds to the total number of guest molecules. Fig. 7 depicts the time evolution of the interaction energy *U*_Int_ for Phth embedded in MOF-5 and MIL-68(Ga), thereby considering three different degrees of loading.

The time series reflects the instantaneous changes in the configuration of the host material, including variations in the cell parameters in the MOF-5 case. These oscillations that are replicated in all neighboring cells due to the periodic setup of the DFTB calculations, are responsible for the very large oscillations in *U*_Int_. However, as seen from the associated running averages also shown in Fig. 7, these oscillations in the energy are short-lived and cancel over the course of the simulation. When comparing the average values determined for the second half of the simulation, it is evident that the host–guest interaction is significantly stronger in (Phth)_1_@MIL-68(Ga), showing an interaction energy of −152.4 kJ mol^−1^, in contrast to the −63.3 kJ mol^−1^ observed in the (Phth)_1_@MOF-5 case (note: (Phth)_*x*_ denotes the number of Phth molecules inside one MOF pore). These findings are reinforced by an additional simulation executed at the same simulation setting, in which a Phth dimer in its parallel stacked configuration was placed inside of the two host-systems. Within less than 50 ps, the stacked dimer configurations showed a separation in favor of the energetically much more favorable guest–host stacking in which both guest molecules interact directly with the host structure. Nevertheless, when comparing the associated interaction energies of the (Phth)_2_@MOF systems, it is evident that overall interaction energies are more than twice the value obtained for the respective monomers. Thus, although the guest molecules prefer to bind directly to the host structure, a notable contribution arising from the respective guest–guest interaction is included in *U*_Int_. This aspect is even more dominant when considering a loading with three guest molecules per MOF pore.

**Fig. 8:**
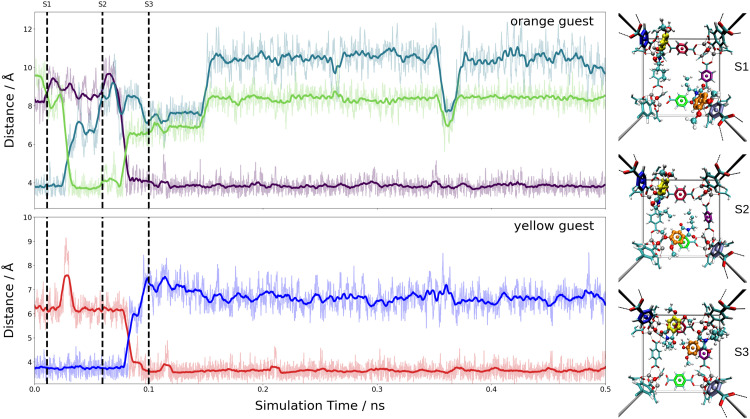
Time evolution of the nearest-neighbor distances between Phth guests and bdc linkers of the Phth_2_@MOF-5 host system analyzed based on the centroids of the aromatic units. The latter were determined as an average over the respective carbon atoms. Snapshots displaying the key configurations encountered along the DFTB MD simulations are marked as S1 to S3. The colors of the nearest-neighbor distances (left) reflect the distances between the inserted Phth to the similarly colored phenyl-ring of the bdc linkers (right).

These estimates for the interaction energy suggest that guest molecules are more strongly bound in MIL-68(Ga) compared to MOF-5, where a higher degree of mobility is expected. As in previous studies,^54,55^ the nearest-neighbor host–guest contacts were monitored using the centroids of the aromatic rings in the bdc linkers and the six-membered rings of the guest molecules. Fig. 8 shows the time evolution of the centroid–centroid distances for (Phth)_2_@MOF-5 (for (Phth)_1_@MOF-5 and (Phth)3@MOF-5 see Fig. S35–S36 (ESI†), ESI†). The guest molecule exhibits a high degree of mobility, resulting in several structural reorientations in the nearest-neighbor contacts over the course of the simulation. Fig. 8 also provides snapshots of several key configurations labeled as S1, S2 and S3. The distance plots suggest that the lifetime of these configurations can vary greatly. In all configurations observed during the simulations, the aromatic rings of the guest molecules form a parallel stacked configuration with the aromatic parts of individual bdc linkers. Furthermore, the Phth molecules tend to align themselves close to each other within the MOF-5 pore, which can be seen in Fig. 8 and Fig. S35 and S36 (ESI†).

**Fig. 9:**
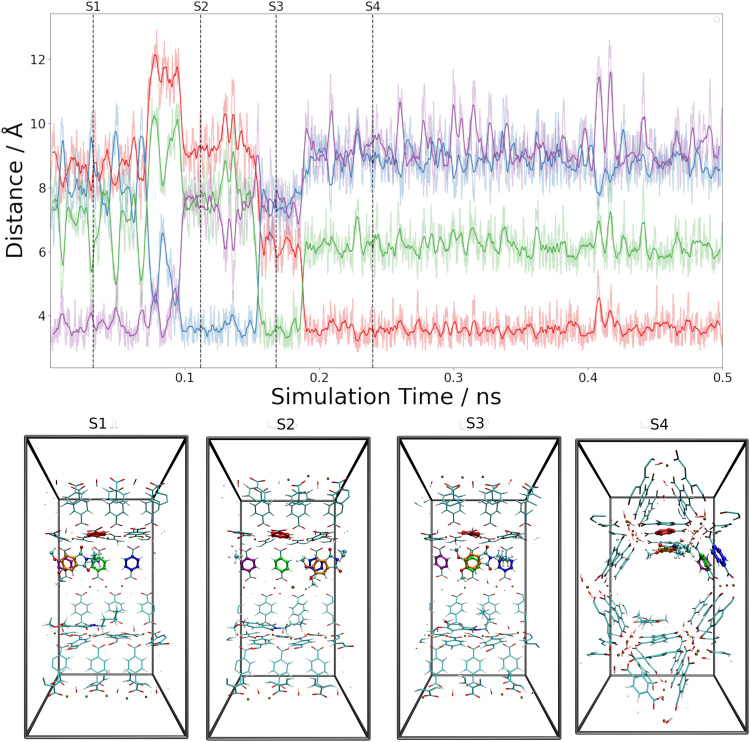
Time evolution of the nearest-neighbor distances between Phth guests and bdc linkers of the Phth_2_@MIL-68(Ga) host system analyzed based on the centroids of the aromatic units. The latter were determined as an average over the respective carbon atoms. Snapshots displaying the key configurations encountered along the DFTB MD simulations are marked as S1 to S4. The colors of the nearest-neighbor distances (top) reflect the distances between the inserted Phth to the similarly colored phenyl-ring of the bdc linkers (bottom).

When following the centroid–centroid distances described above in the (Phth)_2_@MIL-68(Ga) system over the course of the simulation (*cf.*Fig. 9; for (Phth)_1_@MIL-68(Ga) and(Phth)_3_@MIL-68(Ga), see Fig. S37 and S38, ESI†), it is evident that the guest molecule moves along the inner circumference of the large pore channel as illustrated in snapshots S1, S2 and S3, respectively. Although this mobility may seem contradictory to the higher interaction energies, it is important to consider the less confined structural nature of MIL-68(Ga) compared to MOF-5. While MOF-5 can be viewed as a nearly closed cube, MIL-68 systems display a more channel-like structure, with neighboring bdc linker units in a regular, parallel arrangement. However, movements perpendicular to this open axis, as shown in snapshot S4, are generally less frequent than in MOF-5 as already observed in a similar MD study of thioindigo embedded in MOF-5 and MIL-68(Ga).^47^ This can be explained by the tilting of the bdc linkers, leading to the characteristic structure of MOF-5 being composed of alternating large and small pores. This in turn can be expected to lead to a different pattern in the migration of the guest throughout the host material.

Both the higher mobility of Phth inside MOF-5 as well as the fact that the guest molecules arrange themselves next to each other (see Fig. 8 and Fig. S35, S36, ESI†) can explain the increasing blue-shift of the emission upon a higher degree of loading (see Fig. 6). While the guest molecules strongly bind to MIL-68(Ga) and only move along the channels within this MOF host, the Phth molecules are more closely oriented towards each other as a result of the cubic MOF-5 pore and therefore influencing each other. A detailed insight into the local structures *via* solid-state NMR and total X-ray scattering coupled to PDF (= pair distribution function) analysis should be pursued in the future to resolve the interaction patterns structurally and correlate them with the occurring optical properties.

## Conclusion

In the course of the present work, the polarity of ten different host matrices was qualitatively examined *via* the incorporation of the fluorosolvatochromic dye Phth (= 2-butyl-5,6-dimethoxyisoindoline-1,3-dione), confirming the ranking previously established through the embedment of the two solvatochromic and photochromic dyes, spiropyran^10^ and spirooxazine.^8^ Nine MOFs, differing in their nodes, organic linkers, and pore structures, were selected, along with a purely inorganic material, namely zeolite Y. The observed emission characteristics of Phth within these porous hosts were related to the nature of the metal cations, organic linkers and pore sizes. In contrast to solvents, it is postulated for MOFs that a polarity ranking must be defined not only by their permittivities but also by the given structure. Furthermore, the impact of varying guest concentrations within two distinct MOFs on their emission properties was examined. It was observed that in the case of the channel-like structured MIL-68(Ga), no alterations were detected, while for MOF-5 with a cubic pore structure, a blue shift with increasing guest loading was evident. These results were further corroborated by molecular dynamics simulations executed at DFTB level of theory. Based on a detailed analysis of the interaction energies as function of loading as well as the associated guest mobility, a detailed picture of host–guest interactions was provided. The simulation data have shown that strong π–π interactions between the bdc linker and the aromatic moiety of the Phth guest are preferred, even when explicitly considering guest–guest stacking in the initial configuration of the MD simulation. Furthermore, a high degree of mobility of the guest molecules on the sub-nanosecond scale has been observed. In case of MOF-5, the guest molecules have been found to avoid interactions with the inorganic ZnO_4_^6+^-clusters, while preferring parallel stacking with different bdc linkers. To migrate from one linker to the next, comparably large distances have to be overcome. Due to the denser arrangement of bdc linkers along the pore channels in MIL-68(Ga), a much stronger host–guest interaction has been observed. However, similar as in the case of MOF-5, the MD simulations have shown that the Phth guests prefer to form parallel π–π stacking interactions with the bdc linkers, thereby migrating along the inner circumference of the large pore. The different behaviour of the Phth molecules embedded in two host materials MOF-5 and MIL68(Ga) observed from the MD simulations are a direct consequence of the differences in the host–guest interactions at the molecular level.

Conclusively, the determination of polarity on inner surfaces of porous materials is crucial for understanding their chemistry and physics. Incorporating polarity-sensitive dyes is an effective method for such investigations, though some questions remain unanswered. To gain deeper insights, detailed structural investigations are necessary to locate Phth guests within the pores of different hosts and visualize their interactions. However, such investigations are complex due to potential disorder of guest molecules, with only a few examples reported to date.^15,16^ A potential solution to resolve this disorder issue is to combine total X-ray scattering analysis/PDF with solid-state NMR spectroscopy, as demonstrated in the structure determination of a SNP-silica nanocomposite.^56^ These findings will significantly impact further research on inner surface properties of porous compounds, spanning metal–organic frameworks, zeolites, and purely organic materials *e.g.*, COFs.^57^

## Experimental

### Syntheses of the host materials

The host materials MOF-5, MIL-68(In), MIL-68(Ga), MIL-53(Al), MFM-300(Ga_2_), UoC-2(Ga,1F), UoC-2(Ga,2F), UiO-66 and ZIF-8 were synthesized according to the literature known procedures. Commercially available *N*,*N*′-dimethylformamide, DMF (Acros Organics), Ga(NO_3_)_3_·*x*H_2_O (ABCR), In(NO_3_)_3_·5H_2_O (ABCR), Al(NO_3_)_3_·9H_2_O (ABCR), Zn(NO_3_)_3_·6H_2_O (Sigma Aldrich), ZrCl_4_ (Sigma Aldrich), triethylamine, Zn(OAc)_2_·2H_2_O, chloroform (Alfa Aesar), terephthalic acid (Alfa Aesar), tetrahydrofuran (Acros Organics), 2-methylimidazole (Sigma Aldrich), 4,5-dimethoxy-phthalic acid anhydride (ABCR), *n*-butylamine (ABCR), NaOAc (ABCR) and AcOH (ABCR) were used without further purification. Zeolite Y was used as purchased (Alfa Aesar).

#### MOF-5(Zn_4_O(bdc)_3_)(bdc = benzenedicarboxylate)

MOF-5 was synthesized following the protocol given in the literature.^58^ 1.266 g terephthalic acid (7.62 mmol) and triethylamine (2.13 ml) were dissolved in 100 ml of DMF. 4.25 g Zn(OAc)_2_·2H_2_O (19.35 mmol) was dissolved in 125 ml DMF. While stirring, the zinc salt solution was added dropwise to the organic solution over 15 min. The mixture was stirred for 2.5 h. The precipitate was filtered off and immersed in 62.5 ml DMF overnight. It was then filtered off again and immersed in 87.5 ml CHCl_3_. The solvent was exchanged 3 times over 7 days. Finally, the precipitate was decanted. To remove the solvent completely, the resulting powder was heated at 100 °C for 12 h under reduced pressure and stored under an argon atmosphere in the glovebox to prevent absorption of humidity and decomposition by contact with moisture.

#### MIL-68(Ga) (GaOH(bdc)·0.9DMF·*z*H_2_O) and MIL-68(In) (InOH(bdc)·1.0DMF·*z*H_2_O)

MIL-68(Ga) and MIL-68(In) were synthesized by following mainly the protocol given in the literature.^32^

For MIL-68(In), 408.20 mg In(NO_3_)_3_·5H_2_O (1.04 mmol) and 200.00 mg terephthalic acid (1.20 mmol) were mixed with 5.0 ml DMF in a 23 ml Teflon lined autoclave. The mixture was heated to 100 °C with a rate of 20 °C h^−1^, kept at this temperature for 48 h, and afterwards cooled down to room temperature with a rate of 5 °C h^−1^. The resulting colourless powder was washed several times with DMF and dried in air. To remove the embedded DMF molecules, the powder was heated in air at 200 °C for 12 h, then at 100 °C for 1 h under reduced pressure and was stored afterwards under an argon atmosphere in the glovebox to prevent absorption of humidity.

For MIL-68(Ga), 207.40 mg Ga(NO_3_)_3_·*x*H_2_O (0.81 mmol) and 100.00 mg terephthalic acid (0.60 mmol) were mixed with 5.0 ml DMF in a 23 ml Teflon lined autoclave. The mixture was heated to 100 °C with a rate of 20 °C h^−1^, kept at this temperature for 48 h, and afterwards cooled down to room temperature with a rate of 5 °C h^−1^. The resulting colourless powder was washed several times with DMF and dried in air. To remove the embedded DMF molecules, the residue was heated in air at 200 °C for 12 h, then at 100 °C for 1 h under reduced pressure and was stored under an argon atmosphere in the glovebox to prevent absorption of humidity.

#### MIL-53(Al) (AlOH(bdc))

MIL-53(Al) was synthesized according to the protocol known in the literature^33^ with variations in the amount of the starting materials and the applied temperature programs. 1.95 g Al(NO_3_)_3_·9 H_2_O (5.20 mmol) and 432.00 mg of terephthalic acid (2.60 mmol) were mixed with 5.0 ml of deionized water in a 23 ml Teflon lined autoclave. The mixture was heated to 180 °C at 10 °C h^−1^, kept at this temperature for 72 h, and afterwards cooled to room temperature at 5 °C h^−1^. The resulting colourless powder was washed with deionized water several times and dried in air. To remove the embedded terephthalic acid molecules, the residue was heated in air at 350 °C for 7 days followed by 3 days of heating at 400 °C. Finally, the resulting powder was heated at 100 °C for 1 h under reduced pressure and stored under an argon atmosphere in the glovebox to prevent absorption of humidity.

#### MFM-300(Ga_2_) (Ga_2_(OH)_2_(bptc) (bptc = biphenyltetracarboxylate))

The synthesis of MFM-300(Ga_2_) was conducted following the literature known procedure.^34^ Biphenyl-3,3′,5,5′ tetracarboxylic acid (H_4_-bptc, 21.8 mg, 0.067 mmol) and Ga(NO_3_)_3_·*x*H_2_O (calculated for the monohydrate: 38.0 mg, 0.148 mmol) were dissolved in a mixture of DMF, THF and water (2 : 5 : 1, v : v : v, 8 ml) and two drops of hydrochloric acid (37%) were added. The solution was heated for 72 hours at 80 °C in a Teflon lined 20-ml Parr pressure vessel. The obtained colourless powder was washed with DMF several times and dried for 1 h at 100 °C under reduced pressure. Afterwards, the product was kept under argon atmosphere to prevent absorption of humidity.

#### UoC-2(Ga,1F) (Ga_2_(OH)_2_(1F-bptc))

The synthesis of UoC-2(Ga,1F) was conducted as described in the literature.^35^ 4-Fluoro-biphenyl-3,3′,5,5′ tetracarboxylic acid (H_4_-4-1F-bptc, 20.0 mg, 0.06 mmol) and Ga(NO_3_)_3_·*x*H_2_O (calculated for the monohydrate: 100.5 mg, 0.36 mmol) were dissolved in a mixture of 8 ml DMF/THF/H_2_O (2 : 5 : 1, v : v : v, 8 ml) and 15 drops of hydrochloric acid (37%) were added. The solution was heated for 72 hours at 80 °C in a Teflon lined 20-ml Parr pressure vessel. The obtained colourless powder was washed with DMF several times and dried for 1 h at 100 °C under reduced pressure. Afterwards, the product was kept under argon atmosphere to prevent absorption of humidity.

#### UoC-2(Ga,2F) (Ga_2_(OH)_2_(2F-bptc))

UoC-2(Ga,2F) was synthesized according to the literature.^35^ 42.6 mg (0.11 mmol) 4,4′-fluoro-biphenyl-3,3′,5,5′ tetracarboxylic acid (H_4_-4,4′-2F-bptc) and 63.9 mg (calculated for the monohydrate, 0.23 mmol, 2.0 eq.) Ga(NO_3_)_3_·*x*H_2_O were dissolved in DMF/H_2_O (2 : 1, v : v, 8 ml) and two drops of conc. hydrochloric acid (37%) were added. The solution was heated for 72 hours at 80 °C in a Teflon lined 20-ml Parr pressure vessel. The obtained colourless powder was washed with DMF several times and dried for 1 h at 100 °C under reduced pressure. Afterwards, the product was kept under argon atmosphere to prevent absorption of humidity.

#### UiO-66 (Zr_6_O_4_(OH)_4_(bdc)_6_)

UiO-66 was synthesized according to the procedure described in the literature.^36^ ZrCl_4_ (0.212 g, 0.908 mmol) and terephthalic acid (0.136 g, 0.908 mmol) were dissolved in 120 ml DMF. The mixture was sealed and placed in a pressure stable glass vessel and heated to 120 °C for 24 h. Subsequently, the mixture was cooled down to RT under static conditions. The resulting colorless powder was filtered, repeatedly washed with DMF, dried at 100 °C for 1 h under reduced pressure. The product was stored under an argon atmosphere in the glovebox to prevent absorption of humidity.

#### ZIF-8 (ZnC_6_H_6_N_4_)

ZIF-8 was synthesized following the protocol given in the literature.^59^ A solution of Zn(NO_3_)_2_·6H_2_O (1.174 g, 3.95 mmol) in 8 ml deionized water was added dropwise to a solution of 2-methylimidazole (22.714 g, 276.66 mmol) in 80 ml deionized water in a round flask. The mixture was stirred for about an hour. After that, the precipitate was centrifuged. The supernatant was decanted, and the solid residue was dried in an oven at 100 °C overnight. Afterwards, the product was dried for 1 h at 100 °C under a reduced pressure and stored under an argon atmosphere to prevent absorption of humidity.

### Synthesis of the dye 2-butyl-5,6-dimethoxyisoindoline-1,3-dione

2-Butyl-5,6-dimethoxyisoindoline-1,3-dione was synthesized by following the protocol given in the literature.^28^ 300 mg (1.44 mmol, 1.0 eq.) 4,5-dimethoxy-phthalic acid anhydride, 0.16 ml (1.58 mmol, 1.1 eq.) *n*-butylamine and 118 mg (1.44 mmol, 1.0 eq.) NaOAc were heated to reflux for 4 h at 125 °C in 20 ml of AcOH. After cooling to RT, the organic solution was poured into ice-cold water, the precipitate filtered and washed with water. Afterwards, the residue was dried over phosphorous pentoxide. The phase purity was confirmed *via* liquid-state NMR measurements.

^1^H-NMR (300 MHz, CDCl_3_): *δ* [ppm] = 7.29 (s, 2H, H3), 3.99 (s, 6H, H5), 3.64 (t, *J* = 7.3 Hz, 2H, H6), 1.69–1.59 (m, 2H, H7), 1.40–1.32 (m, 2H, H8), 0.94 (t, *J* = 7.3 Hz, 3H, H9); ^13^C-NMR (75 MHz, CDCl3): *δ* [ppm] = 168.6 (C1), 153.7 (C4), 125.5 (C2), 105.3 (C3), 56.6 (C5), 37.8 (C6), 30.8 (C7), 20.1 (C8), 13.7 (C9).

### Preparation of Phth@PM systems

A mixture of the respective activated MOF and an excess of 2-butyl-5,6-dimethoxyisoindoline-1,3-dione was ground thoroughly under an argon atmosphere. The resulting homogeneous powder was placed into a small glass vessel inside a Schlenk tube and heated to 120 °C to 130 °C and a reduced pressure of ∼5 × 10^−2^ mbar for several hours. The excess of Phth, which cannot enter the pores when the maximum loading capacity of the respective porous host material is reached, resublimed at the top of the glass tube. To prevent the absorption of water and decomposition upon contact with air and moisture, all compounds were stored in a glovebox under an argon atmosphere.

#### Phth_*x*_@MOF-5 (1)

The synthesis was carried out as described above with 60.00 mg (0.08 mmol) MOF-5 and 61.0 mg (0.24 mmol) Phth yielding a colorless powder. The weighed in molar ratio of the guest to host is 3 : 1.

#### Phth_*x*_@MIL-68(In) (2)

The synthesis was carried out as described above with 60.00 mg (0.20 mmol) MIL-68(In) and 53.4 mg (0.20 mmol) Phth yielding a colorless powder. The weighed in molar ratio of the guest to host is 1 : 1.

#### Phth_*x*_@MIL-68(Ga) (3)

The synthesis was carried out as described above with 60.00 mg (0.24 mmol) MIL-68(Ga) and 63 mg (0.24 mmol) Phth yielding a colorless powder. The weighed in molar ratio of the guest to host is 1 : 1.

#### Phth_*x*_@MIL-53(Al) (4)

The synthesis was carried out as described above with 60.00 mg (0.28 mmol) MIL-53(Al) and 75.9 mg (0.28 mmol) Phth yielding a colorless powder. The weighed in molar ratio of the guest to host is 1 : 1.

#### Phth_*x*_@MFM-300(Ga_2_) (5)

The synthesis was carried out as described above with 30.00 mg (0.06 mmol) MFM-300(Ga_2_) and 16.0 mg (0.06 mmol) Phth yielding a colorless powder. The weighed in molar ratio of the guest to host is 1 : 1.

#### Phth_*x*_@UoC-2(Ga,1F) (6)

The synthesis was carried out as described above with 30.00 mg (0.057 mmol) UoC-2(Ga,1F) and 15 mg (0.057 mmol) Phth yielding a colorless powder. The weighed in molar ratio of the guest to host is 1 : 1.

#### Phth_*x*_@UoC-2(Ga,2F) (7)

The synthesis was carried out as described above with 60.00 mg (0.11 mmol) UoC-2(Ga,2F) and 29.4 mg (0.11 mmol) Phth yielding a colorless powder. The weighed in molar ratio of the guest to host is 1 : 1.

#### Phth_*x*_@UiO-66 (8)

The synthesis was carried out as described above with 30.00 mg (0.181 mmol) UiO-66 and 4.8 mg (0.181 mmol) Phth yielding a colorless powder. The weighed in molar ratio of the guest to host is 1 : 1.

#### Phth_*x*_@ZIF-8 (9)

The synthesis was carried out as described above with 60.00 mg (0.30 mmol) ZIF-8 and 69.41 mg (0.26 mmol) Phth yielding a colorless powder. The weighed in molar ratio of the guest to host is 1 : 1.

#### Phth_*x*_@Zeolite Y (10)

The synthesis was carried out as described above with 60.00 mg (0.14 mmol) zeolite Y and 97.5 mg (0.14 mmol) Phth yielding a colorless powder. The weighed in molar ratio of the guest to host is 1 : 1.

## X-ray powder diffraction

### Laboratory measurements

Generally, for each measurement, samples were prepared under an argon atmosphere to prevent absorption of humidity and decomposition. For that purpose, the respective MOF or hybrid system was thoroughly ground and filled about 2 cm high into Lindemann glass capillaries (diameter of 0.7 mm). The filled capillaries were sealed with the help of a glow wire and closed airtight with pizine. Samples were adjusted on a goniometer head in a way that the substance was in the beam the whole time while rotating. Measurements were carried out on three different devices:

(1) Measurements were performed on a Huber G670 powder diffractometer (Guinier geometry, Ge (111) monochromator, image plate detector) with Cu-K_α1_ radiation. Data were collected at 298 K between 4 and 80.70° in 2*θ* with steps of 0.005° and a measurement time of 2 s per step.

(2) MIL-53(Al) ht was measured on a STOE Stadi P diffractometer with Cu-K_α1_ radiation (*λ* = 1.5406 Å) utilizing a focusing Ge(111) primary beam monochromator and a PSD detector. Data were collected at 298 K between 4° and 80.70° in 2*θ* with steps of 0.01° and a measurement time of 5 s per step. Seven such scans were added for one measurement.

(3) Measurements were performed on a STOE Stadi P diffractometer (Stoe, Darmstadt, Germany) in transmission geometry with Mo-K_α1_-radiation (*λ* = 0.7093 Å) utilizing a focusing Ge(111) primary beam monochromator and a Mythen 2 DCS4 detector. Data were collected in the 2*θ* range of 2.0–40.4° with a step size of 0.015°.

### High resolution synchrotron powder diffraction measurements

High resolution diffraction data were recorded at BL9 of the Dortmunder Elektronen-Speicherring-Anlage (DELTA). Data were collected at room temperature with a wavelength of 0.49594 Å using a PILATUS100K detector between 2.8° and 25° in 2*θ* with steps of 0.01° and 10 s integration time per data point.

### X-ray photoelectron spectroscopy (XPS)

For XPS measurements, Phth@MOF powders were placed on an adhesive copper foil. Measurements were performed on a multichamber UHV system at a pressure of 5 × 10^−10^ mbar using a Phoibos 100 hemispherical analyzer (Specs). As the excitation source, a Mg K_α_ anode was used (*hν* = 1252.6 eV, probing depth ∼10 nm). Due to charging effects during measurements caused by the low conductivity of the powder samples, the binding energy scale as measured by XPS was shifted by a few electronvolts for the different samples. To account for this, the binding energies were corrected such that adventitious carbon is positioned at 284.8 eV. Integrated peak areas of characteristic core level excitations were used to calculate the embedded amount of Phth inside the MOF matrices. For this, the peak areas of N, Zn, In, Ga, Al and Zr were evaluated and corrected by their relative sensitivity factors (RSF).^60^

### Fluorescence spectroscopy

Solid-state fluorescence spectra of Phth@PM samples were recorded on a PerkinElmer LS-50B Fluorescence spectrometer. The samples were placed into the sample holder under Argon atmosphere and kept there right before the measurement started. Scans were done in the range of 350 nm to 700 nm with an excitation wavelength of *λ*_ex_ = 347 nm and 340 nm.

Liquid-state fluorescence spectra of the phthalimide were recorded on a PerkinElmer LS-50B Fluorescence spectrometer. A concentration of 10^−5^ mol l^−1^ was chosen based on the sensitivity of the fluorescence spectrometer's detector.

### Liquid-state NMR spectroscopy

^1^H and ^13^C NMR spectra were recorded at room temperature on Bruker instruments. Chemical shifts are given in ppm relative to the signal of the deuterated solvent (^13^C NMR) or non-deuterated traces of the solvent (^1^H NMR). Deuterated chloroform CDCl_3_ [*δ*(^1^H) = 7.24 ppm; *δ*(^13^C) = 77.0 ppm] from Deutero GmbH was used as solvent. The description of the fine structure of the signals in the ^1^H NMR spectrum was given by the abbreviations “s” (singlet), “d” (doublet), “dd” (double-doublet), “t” (triplet), “q” (quadruplet), and “m” (multiplet). The multiplicity of the carbon signals was determined by APT (attached proton test) experiments. The coupling constants J are given in Hertz (Hz).

### SCC DFTB calculation setup

To obtain detailed insight into the host–guest interaction of Phth embedded in MOF-5 and MIL-68(Ga), molecular dynamic (MD) simulations in conjunction with the self-consistent charge density functional tight binding (SCC DFTB) were conducted. Previously, this level of theory has successfully been applied to describe similar systems as those explored in this study.^47^ In case of MOF-5, DFTB of third order (*i.e.* DFTB3) in conjunction with the 3ob parameter set^61–63^ as implemented in the DFTB+ package^64^ was applied. In case of the MIL-68(Ga)-system, second order DFTB (DFTB2) utilizing the mio/hyb parameter set^65,66^ was used, as it has already been applied in previous studies of MIL68(Ga).^44,45^ In order to apply the latter method for the description of Phth@MIL68(Ga), the mio/hyb parameter set had to be extended to include the Ga-N interactions as described in the following:

### Parametrization of the DFTB2 Ga-N Interactions

The creation of the Slater–Koster tables for the heteronuclear Ga–N interaction was carried out based on the workflow outlined by Van den Bossche *et al.* using the program hotcent.^67,68^ The confinement parameters for Ga and N were taken from the literature as given in the mio set^69,70^ and its respective extensions.^65,66^

DFTB2^71^ parameters consist of two separate sets of parameters, the electronic part describing the electronic interactions of the atoms in the calculation, and the repulsive part, a pairwise-additive interaction term fitted to relevant reference geometries.^70,72^ The parametrization of the electronic part usually involves the calculation of one-center parameters such as orbital eigenvalues and associated Hubbard *U* values in addition to the evaluation of suitable confinement potentials for the orbitals and the electron density. However, as the mio set^69,70^ and the respective extensions^65,66^ already include parameters for gallium and nitrogen, the one-center parameters as well as the respective confinement potential data required for the creation of the Ga–N Slater–Koster table are readily available. The latter were created using the hotcent^67,68^ package employing the Perdew–Burke–Enzerhofer functional.^73^

The repulsive interaction terms are comprised of a short-range exponential function followed by several spline functions (typically featuring intervals in the range of 0.01 to 0.05 bohr) until a preset cutoff radius. The latter is usually set in the order of two to three times of the respective covalent radii.^72^ The interactions were fitted to data of potential energy scans of a planar Ga(NH_2_)_3_ molecule, executed with Gaussian16 at B3LYP/SDD+6-31G(d) level^74–79^ with the tango program package^80^ employing the DFTB+ program package using the previously created Slater–Koster tables as input.

### Molecular dynamics simulation protocol

The DFTB+ package was integrated with the in-house developed MD simulation routines,^81–84^ specifically designed for solid state systems, to perform the SCC DFTB MD simulations. To accommodate holonomic bond constraints for bonds involving hydrogen atoms, the SHAKE/RATTLE algorithm^85,86^ was employed, enabling an increased MD time step of 2.0 fs in the time integration. The latter was realized using the velocity Verlet propagator.^85^

To achieve constant temperature conditions at 298.15 K within the individual simulations, the Bussi–Donadio–Parrinello thermostat algorithm^87^ was applied, while constant pressure conditions at 1.013 bar were realized using the Berendsen manostat algorithm.^88^ The respective relaxation times were set to 0.1 and 5 ps in case of MOF-5. Contrary to the case of MOF-5, the simulations of the MIL-68(Ga) have to be carried out in a canonical ensemble (NVT) as discussed in previous works.^44,47^ It was shown that MIL-68(Ga) undergoes a phase transition under constant pressure conditions resulting in a collapse of the nanoporous structure. Nevertheless, the properties obtained under NVT conditions still provide adequate structural characteristics.^44,47^ For this reason, only the Bussi-Donadio-Parrinello thermostat algorithm with a corresponding relaxation time of 0.1 ps was applied in this case.

Snapshots of key configurations within the simulations were generated using the VMD (visual molecular dynamics) package.^29^ The initial configurations for all simulations were generated from equilibrated systems of the pristine MOFs taken from previous research.^47^ Next, the guests were randomly placed into the large pores of the target MOF while maintaining a sensible distance (*i.e.* >1.5 Å) between host and guest atoms.

In the case of MOF-5, each system underwent an equilibration phase of at least 5000 MD steps (0.01 ns) under NpT conditions to achieve an adequate relaxation of the cell parameters, followed by a comprehensive sampling phase of 250 000 MD steps (0.5 ns). Since no adjustment of the unit cell is feasible in the NVT simulation of MIL-68(Ga), an adjustment of the cell parameters was not required.

## Conflicts of interest

There are no conflicts to declare.

## Supplementary Material

TC-012-D4TC01401D-s001

## References

[cit1] Batten S. R., Champness N. R., Chen X.-M., Garcia-Martinez J., Kitagawa S., Öhrström L., O’Keeffe M., Suh M. P., Reedijk J. (2013). Terminology of Metal–Organic Frameworks and Coordination Polymers (IUPAC Recommendations 2013). Pure Appl. Chem..

[cit2] Ohara K., Inokuma Y., Fujita M. (2010). The Catalytic *Z* to *E* Isomerization of Stilbenes in a Photosensitizing Porous Coordination Network. Angew. Chem., Int. Ed..

[cit3] Yanai N., Uemura T., Inoue M., Matsuda R., Fukushima T., Tsujimoto M., Isoda S., Kitagawa S. (2012). Guest-to-Host Transmission of Structural Changes for Stimuli-Responsive Adsorption Property. J. Am. Chem. Soc..

[cit4] Müller K., Wadhwa J., Singh Malhi J., Schöttner L., Welle A., Schwartz H., Hermann D., Ruschewitz U., Heinke L. (2017). Photoswitchable Nanoporous Films by Loading Azobenzene in Metal–Organic Frameworks of Type HKUST-1. Chem. Commun..

[cit5] Eichler C., Rázková A., Müller F., Kopacka H., Huppertz H., Hofer T. S., Schwartz H. A. (2021). Paving the Way to the First Functional Fulgide@MOF Hybrid Materials. Chem. Mater..

[cit6] Kremer S., Ober I., Greussing V., Kopacka H., Gallmetzer H. G., Demmel D., Olthof S., Huppertz H., Schwartz H. A. (2021). Modulating the Optical Characteristics of Spiropyran@Metal–Organic Framework Composites as a Function of Spiropyran Substitution. Langmuir.

[cit7] Greussing V., Gallmetzer J. M., Huppertz H., Hofer T. S., Schwartz H. A. (2022). Optical Characteristics of Spiropyran@MOF Composites as a Function of the Metal–Organic Framework Linker Substitution. J. Phys. Chem. C.

[cit8] Schwartz H. A., Werker M., Tobeck C., Christoffels R., Schaniel D., Olthof S., Meerholz K., Kopacka H., Huppertz H., Ruschewitz U. (2020). Novel Photoactive Spirooxazine Based Switch@MOF Composite Materials. ChemPhotoChem.

[cit9] Garg S., Schwartz H., Kozlowska M., Kanj A. B., Müller K., Wenzel W., Ruschewitz U., Heinke L. (2019). Conductance Photoswitching of Metal–Organic Frameworks with Embedded Spiropyran. Angew. Chem., Int. Ed..

[cit10] Schwartz H. A., Olthof S., Schaniel D., Meerholz K., Ruschewitz U. (2017). Solution-Like Behavior of Photoswitchable Spiropyrans Embedded in Metal–Organic Frameworks. Inorg. Chem..

[cit11] Walton I. M., Cox J. M., Coppin J. A., Linderman C. M., Patel D. G. (Dan), Benedict J. B., Ren H., Zhu G. (2013). Photo-Responsive MOFs: Light-Induced Switching of Porous Single Crystals Containing a Photochromic Diarylethene. Chem. Commun..

[cit12] Zhang F., Zou X., Feng W., Zhao X., Jing X., Sun F., Ren H., Zhu G. (2012). Microwave-Assisted Crystallization Inclusion of Spiropyran Molecules in Indium Trimesate Films with Antidromic Reversible Photochromism. J. Mater. Chem..

[cit13] Das D., Agarkar H. (2018). Unexpected Nonresponsive Behavior of a Flexible Metal-Organic Framework under Conformational Changes of a Photoresponsive Guest Molecule. ACS Omega.

[cit14] Shepherd N. D., Wang T., Ding B., Beves J. E., D’Alessandro D. M. (2021). Visible Light Stimulated Bistable Photo-Switching in Defect Engineered Metal–Organic Frameworks. Inorg. Chem..

[cit15] Hermann D., Schwartz H. A., Werker M., Schaniel D., Ruschewitz U. (2019). Metal–Organic Frameworks as Hosts for Fluorinated Azobenzenes: A Path towards Quantitative Photoswitching with Visible Light. Chem. – Eur. J..

[cit16] Hermann D., Emerich H., Lepski R., Schaniel D., Ruschewitz U. (2013). Metal–Organic Frameworks as Hosts for Photochromic Guest Molecules. Inorg. Chem..

[cit17] Reichardt C. , Solvents and Solvent Effects in Organic Chemistry, WILEY VCH Verlag GmbH, Weinheim, 3rd edn, 2003 10.1002/3527601791

[cit18] Dolgopolova E. A., Moore T. M., Ejegbavwo O. A., Pellechia P. J., Smith M. D., Shustova N. B. (2017). A Metal–Organic Framework as a Flask: Photophysics of Confined Chromophores with a Benzylidene Imidazolinone Core. Chem. Commun..

[cit19] Dolgopolova E. A., Berseneva A. A., Faillace M. S., Ejegbavwo O. A., Leith G. A., Choi S. W., Gregory H. N., Rice A. M., Smith M. D., Chruszcz M., Garashchuk S., Mythreye K., Shustova N. B. (2020). Confinement-Driven Photophysics in Cages, Covalent-Organic Frameworks, Metal–Organic Frameworks, and DNA. J. Am. Chem. Soc..

[cit20] Leith G. A., Martin C. R., Mayers J. M., Kittikhunnatham P., Larsen R. W., Shustova N. B. (2021). Confinement-Guided Photophysics in MOFs, COFs, and Cages. Chem. Soc. Rev..

[cit21] Hirai K., Kitagawa T., Fujiwara H., Pirillo J., Hijikata Y., Inose T., Uji-I H. (2020). Multicolour Photochromic Fluorescence of a Fluorophore Encapsulated in a Metal–Organic Framework. Chem. Commun..

[cit22] Tyutyulkov N. , Fabian J., Mehlhorn A., Dietz F. and Tadjer A., Polymethine Dyes - Strucuture and Properties, St. Kliment Ohridski University Press, Sofia, 1991

[cit23] Ishchenko A. A. , Constitution and Spectral-Luminescent Properties of Polymethine Dyes, Naukova Dumka: Kiew, 1994

[cit24] Langhals H. (1987). The Polarity of Solutions of Electrolytes. Tetrahedron.

[cit25] Suppan P. (1987). Local Polarity of Solvent Mixtures in the Field of Electronically Excited Molecules and Exciplexes. J. Chem. Soc., Faraday Trans. 1.

[cit26] Soujanya T., Fessenden R. W., Samanta A. (1996). Role of Nonfluorescent Twisted Intramolecular Charge Transfer State on the Photophysical Behavior of Aminophthalimide Dyes. J. Phys. Chem..

[cit27] Wetzler D. E., Chesta C., Fernández-Prini R., Aramendía P. F. (2001). Dynamic Solvatochromism in Solvent Mixtures. Pure Appl. Chem..

[cit28] Atar M., Öngel B., Riedasch H., Lippold T., Neudörfl J., Sampedro D., Griesbeck A. G. (2020). Intra- and Intermolecular Fluorescence Quenching of Alkylthio-Substituted Phthalimides by Photoinduced Electron Transfer: Distance, Position and Conformational Dependence. ChemPhotoChem.

[cit29] Humphrey W., Dalke A., Schulten K. (1996). VMD: Visual Molecular Dynamics. J. Mol. Graphics.

[cit30] Snyder L. R. , in High Performance Liquid Chromatography, ed. C. Horvath, Academic Press, New York, 3rd edn, 1983

[cit31] Li H., Eddaoudi M., O’Keeffe M., Yaghi O. M. (1999). Design and Synthesis of an Exceptionally Stable and Highly Porous Metal–Organic Framework. Nature.

[cit32] Volkringer C., Meddouri M., Loiseau T., Guillou N., Marrot J., Férey G., Haouas M., Taulelle F., Audebrand N., Latroche M. (2008). The Kagomé Topology of the Gallium and Indium Metal–Organic Framework Types with a MIL-68 Structure: Synthesis, XRD, Solid-State NMR Characterizations, and Hydrogen Adsorption. Inorg. Chem..

[cit33] Loiseau T., Serre C., Huguenard C., Fink G., Taulelle F., Henry M., Bataille T., Férey G. (2004). A Rationale for the Large Breathing of the Porous Aluminum Terephthalate (MIL-53) Upon Hydration. Chem. – Eur. J..

[cit34] Krap C. P., Newby R., Dhakshinamoorthy A., García H., Cebula I., Easun T. L., Savage M., Eyley J. E., Gao S., Blake A. J., Lewis W., Beton P. H., Warren M. R., Allan D. R., Frogley M. D., Tang C. C., Cinque G., Yang S., Schröder M. (2016). Enhancement of CO_2_ Adsorption and Catalytic Properties by Fe-Doping of [Ga_2_(OH)_2_(L)](H_4_L = Biphenyl-3,3′,5,5′-Tetracarboxylic Acid), MFM-300(Ga_2_). Inorg. Chem..

[cit35] Breitenbach (née Stastny) C., Christoffels R., Mattick T., Edelmann A., Pierkes T., König S., Fröba M., Ruschewitz U. (2024). UoC-2: A Fluorinated Derivative of the Robust Metal–Organic Framework MFM-300. Eur. J. Inorg. Chem..

[cit36] Cavka J. H., Jakobsen S., Olsbye U., Guillou N., Lamberti C., Bordiga S., Lillerud K. P. (2008). A New Zirconium Inorganic Building Brick Forming Metal Organic Frameworks with Exceptional Stability. J. Am. Chem. Soc..

[cit37] Karagiaridi O., Lalonde M. B., Bury W., Sarjeant A. A., Farha O. K., Hupp J. T. (2012). Opening ZIF-8: A Catalytically Active Zeolitic Imidazolate Framework of Sodalite Topology with Unsubstituted Linkers. J. Am. Chem. Soc..

[cit38] Breck D. W. , Crystalline Zeolite Y. U.S. Patent, 3130007, 1964

[cit39] Brandenburg K. , Diamond 4.4, Crystal Impact GbR, Bonn, 2017

[cit40] Yaghi O. M., Keeffe O., Ockwig M., Chae N. W., Eddaoudi H. K., Kim M. (2003). J. Reticular Synthesis and the Design of New Materials. Nature.

[cit41] Valenzano L., Civalleri B., Chavan S., Bordiga S., Nilsen M. H., Jakobsen S., Lillerud K. P., Lamberti C. (2011). Disclosing the Complex Structure of UiO-66 Metal Organic Framework: A Synergic Combination of Experiment and Theory. Chem. Mater..

[cit42] Park K. S., Ni Z., Côté A. P., Choi J. Y., Huang R., Uribe-Romo F. J., Chae H. K., O’Keeffe M., Yaghi O. M. (2006). Exceptional Chemical and Thermal Stability of Zeolitic Imidazolate Frameworks. Proc. Natl. Acad. Sci. U. S. A..

[cit43] Kim H. S. (2016). Zeolite Y Film as a Versatile Material for Electrochemical Sensors. Mater. Lett..

[cit44] Purtscher F. R. S., Christanell L., Schulte M., Seiwald S., Rödl M., Ober I., Maruschka L. K., Khoder H., Schwartz H. A., Bendeif E. E., Hofer T. S. (2022). Structural Properties of Metal-Organic Frameworks at Elevated Thermal Conditions *via* a Combined Density Functional Tight Binding Molecular Dynamics (DFTB MD) Approach. J. Phys. Chem. C.

[cit45] Hofer T. S., Listyarini R. V., Hajdarevic E., Maier L., Purtscher F. R. S., Gamper J., Hanser F. (2023). Beyond the Status Quo: Density Functional Tight Binding and Neural Network Potentials as a Versatile Simulation Strategy to Characterize Host–Guest Interactions in Metal- and Covalent Organic Frameworks. J. Phys. Chem. Lett..

[cit46] Listyarini R. V., Gamper J., Hofer T. S. (2023). Storage and Diffusion of Carbon Dioxide in the Metal Organic Framework MOF-5—A Semi-Empirical Molecular Dynamics Study. J. Phys. Chem. B.

[cit47] Fischereder A., Rödl M., Suta M., Hofer T. S., Schwartz H. A. (2023). From Blue Jeans to Luminescent Materials: Designing Thioindigo-Based Red-Fluorescent Hybrid Systems. J. Phys. Chem. C.

[cit48] Liu S., Xiang Z., Hu Z., Zheng X., Cao D. (2011). Zeolitic Imidazolate Framework-8 as a Luminescent Material for the Sensing of Metal Ions and Small Molecules. J. Mater. Chem..

[cit49] Yang C., Kaipa U., Mather Q. Z., Wang X., Nesterov V., Venero A. F., Omary M. A. (2011). Fluorous Metal–Organic Frameworks with Superior Adsorption and Hydrophobic Properties toward Oil Spill Cleanup and Hydrocarbon Storage. J. Am. Chem. Soc..

[cit50] Peikert K., Hoffmann F., Fröba M. (2015). Fluorine Magic: One New Organofluorine Linker Leads to Three New Metal–Organic Frameworks. CrystEngComm.

[cit51] Hernández-Trujillo J., Vela A. (1996). Molecular Quadrupole Moments for the Series of Fluoro- and Chlorobenzenes. J. Phys. Chem..

[cit52] Dimroth K., Reichardt C., Siepmann T., Bohlmann F. (1963). Über Pyridinium-*N*-Phenol-Betaine Und Ihre Verwendung Zur Charakterisierung Der Polarität von Lösungsmitteln. Justus Liebigs Ann. Chem..

[cit53] Reichardt C. (1992). Solvatochromism, Thermochromism, Piezochromism, Halochromism, and Chiro-Solvatochromism of Pyridinium *N*-Phenoxide Betaine Dyes. Chem. Soc. Rev..

[cit54] Rödl M., Kerschbaumer S., Kopacka H., Blaser L., Purtscher F. R. S., Huppertz H., Hofer T. S., Schwartz H. A. (2021). Structural, Dynamical, and Photochemical Properties of *Ortho*-Tetrafluoroazobenzene inside a Flexible MOF under Visible Light Irradiation. RSC Adv..

[cit55] Rödl M., Reka A., Panic M., Fischereder A., Oberlechner M., Mairegger T., Kopacka H., Huppertz H., Hofer T. S., Schwartz H. A. (2022). Fundamental Study of the Optical and Vibrational Properties of Fx-AZB@MOF Systems as Functions of Dye Substitution and the Loading Amount. Langmuir.

[cit56] Bendeif E.-E. E., Gansmuller A., Hsieh K.-Y. Y., Pillet S., Woike T., Zobel M., Neder R. B., Bouazaoui M., El Hamzaoui H., Schaniel D. (2015). Structure Determination of Molecular Nanocomposites by Combining Pair Distribution Function Analysis and Solid-State NMR. RSC Adv..

[cit57] Côté A. P., Benin A. I., Ockwig N. W., O’Keeffe M., Matzger A. J., Yaghi O. M. (2005). Porous, Crystalline, Covalent Organic Frameworks. Science.

[cit58] Tranchemontagne D. J., Hunt J. R., Yaghi O. M. (2008). Room Temperature Synthesis of Metal–Organic Frameworks. Tetrahedron.

[cit59] Pan Y., Liu Y., Zeng G., Zhao L., Lai Z. (2011). Rapid Synthesis of Zeolitic Imidazolate Framework-8 (ZIF-8) Nanocrystals in an Aqueous System. Chem. Commun..

[cit60] Scofield J. H. (1976). Hartree-Slater Subshell Photoionization Cross-Sections at 1254 and 1487 EV. J. Electron Spectros. Relat. Phenom..

[cit61] Gaus M., Goez A., Elstner M. (2013). Parametrization and Benchmark of DFTB3 for Organic Molecules. J. Chem. Theory Comput..

[cit62] Lu X., Gaus M., Elstner M., Cui Q. (2015). Parametrization of DFTB3/3OB for Magnesium and Zinc for Chemical and Biological Applications. J. Phys. Chem. B.

[cit63] Gaus M., Lu X., Elstner M., Cui Q. (2014). Parameterization of DFTB3/3OB for Sulfur and Phosphorus for Chemical and Biological Applications. J. Chem. Theory Comput..

[cit64] Hourahine B., Aradi B., Blum V., Bonafé F., Buccheri A., Camacho C., Cevallos C., Deshaye M. Y., Dumitric T., Dominguez A., Ehlert S., Elstner M., Van Der Heide T., Hermann J., Irle S., Kranz J. J., Köhler C., Kowalczyk T., Kubař T., Lee I. S., Lutsker V., Maurer R. J., Min S. K., Mitchell I., Negre C., Niehaus T. A., Niklasson A. M. N., Page A. J., Pecchia A., Penazzi G., Persson M. P., Řezáč J., Sánchez C. G., Sternberg M., Stöhr M., Stuckenberg F., Tkatchenko A., Yu V. W. Z., Frauenheim T. (2020). DFTB+, a Software Package for Efficient Approximate Density Functional Theory Based Atomistic Simulations. J. Chem. Phys..

[cit65] Szücs B., Hajnal Z., Frauenheim T., González C., Ortega J., Pérez R., Flores F. (2003). Chalcogen Passivation of GaAs(1 0 0) Surfaces: Theoretical Study. Appl. Surf. Sci..

[cit66] Szucs B., Hajnal Z., Scholz R., Sanna S., Frauenheim T. (2004). Theoretical Study of the Adsorption of a PTCDA Monolayer on S-Passivated GaAs(1 0 0). Appl. Surf. Sci..

[cit67] Van Den Bossche M. (2019). DFTB-Assisted Global Structure Optimization of 13- and 55-Atom Late Transition Metal Clusters. J. Phys. Chem. A.

[cit68] Van den Bossche M. (2024). Three-Center Tight-Binding Together with Multipolar Auxiliary Functions. J. Chem. Theory Comput..

[cit69] Elstner M., Porezag D., Jungnickel G., Elsner J., Haugk M., Frauenheim T. (1998). Self-Consistent-Charge Density–Functional Tight-Binding Method for Simulations of Complex Materials Properties. Phys. Rev. B: Condens. Matter Mater. Phys..

[cit70] Yang Y., Yu H., York D., Elstner M., Cui Q. (2008). Description of Phosphate Hydrolysis Reactions with the Self-Consistent-Chage Density–Functional-Tight-Binding (SCC-DFTB) Theory. 1. Parameterization. J. Chem. Theory Comput..

[cit71] Vuong V. Q., Akkarapattiakal Kuriappan J., Kubillus M., Kranz J. J., Mast T., Niehaus T. A., Irle S., Elstner M. (2018). Parametrization and Benchmark of Long-Range Corrected DFTB2 for Organic Molecules. J. Chem. Theory Comput..

[cit72] Koskinen P., Mäkinen V. (2009). Density-Functional Tight-Binding for Beginners. Comput. Mater. Sci..

[cit73] Perdew J. P., Burke K., Ernzerhof M. (1996). Generalized Gradient Approximation Made Simple. Phys. Rev. Lett..

[cit74] Becke A. D. (1993). Density-Functional Thermochemistry. III. The Role of Exact Exchange. J. Chem. Phys..

[cit75] Ditchfield R., Hehre W. J., Pople J. A. (1971). Self-Consistent Molecular–Orbital Methods. IX. An Extended Gaussian-Type Basis for Molecular–Orbital Studies of Organic Molecules. J. Chem. Phys..

[cit76] Hariharan P. C., Pople J. A. (1973). The Influence of Polarization Functions on Molecular Orbital Hydrogenation Energies. Theor. Chim. Acta.

[cit77] Hehre W. J., Ditchfield K., Pople J. A. (1972). Self-Consistent Molecular Orbital Methods. XII. Further Extensions of Gaussian-Type Basis Sets for Use in Molecular Orbital Studies of Organic Molecules. J. Chem. Phys..

[cit78] Rassolov V. A., Ratner M. A., Pople J. A., Redfern P. C., Curtiss L. A. (2001). 6-31G* Basis Set for Third-Row Atoms. J. Comput. Chem..

[cit79] Bergner A., Dolg M., Küchle W., Stoll H., Preuß H. (1993). Ab Initio Energy-Adjusted Pseudopotentials for Elements of Groups 13–17. Mol. Phys..

[cit80] Van Den Bossche M., Grönbeck H., Hammer B. (2018). Tight-Binding Approximation-Enhanced Global Optimization. J. Chem. Theory Comput..

[cit81] Hofer T. S., Tirler A. O. (2015). Combining 2d-Periodic Quantum Chemistry with Molecular Force Fields: A Novel QM/MM Procedure for the Treatment of Solid-State Surfaces and Interfaces. J. Chem. Theory Comput..

[cit82] Saleh M., Hofer T. S. (2019). A DFTB/MM MD Approach for Solid-State Interfaces: Structural and Dynamical Properties of H_2_O and NH_3_ on R-TiO_2_(001). J. Phys. Chem. C.

[cit83] Prasetyo N., Hofer T. S. (2019). Adsorption and Dissociation of Water Molecules at the α-Al_2_O_3_(0001) Surface: A 2-Dimensional Hybrid Self-Consistent Charge Density Functional Based Tight-Binding/Molecular Mechanics Molecular Dynamics (2D SCC-DFTB/MM MD) Simulation Study. Comput. Mater. Sci..

[cit84] Kriesche B. M., Kronenberg L. E., Purtscher F. R. S., Hofer T. S. (2023). Storage and Diffusion of CO_2_ in Covalent Organic Frameworks—A Neural Network-Based Molecular Dynamics Simulation Approach. Front. Chem..

[cit85] Andersen H. C. (1983). Rattle: A “Velocity” Version of the Shake Algorithm for Molecular Dynamics Calculations. J. Comput. Phys..

[cit86] Ryckaert J. P., Ciccotti G., Berendsen H. J. C. (1977). Numerical Integration of the Cartesian Equations of Motion of a System with Constraints: Molecular Dynamics of *n*-Alkanes. J. Comput. Phys..

[cit87] Bussi G., Donadio D., Parrinello M. (2007). Canonical Sampling through Velocity Rescaling. J. Chem. Phys..

[cit88] Berendsen H. J. C., Postma J. P. M., Van Gunsteren W. F., Dinola A., Haak J. R. (1984). Molecular Dynamics with Coupling to an External Bath. J. Chem. Phys..

