# Supplementary material for: Polarity profiling of porous architectures: solvatochromic dye encapsulation in metal–organic frameworks
Source: J Mater Chem C Mater. 2024 May 28;12(24):8759–76. doi: 10.1039/d4tc01401d (PMC11188709; doi:10.1039/d4tc01401d)
Supplement: TC-012-D4TC01401D-s001 [file TC-012-D4TC01401D-s001.pdf]

Electronic Supplementary Material (ESI) for Journal of Materials Chemistry C.
This journal is © The Royal Society of Chemistry 2024

Supporting Information

Heidi A. Schwartz[a,b,*], Murat Atar[a], Matthias Spilles[a], Michael Fill[b], Manuel Ott[b], Felix R. S. Purtscher[b], Josef M. Gallmetzer[b], Baris Öcal[a], Selina Olthof[a], Axel Griesbeck[a], Klaus Meerholz[a], Thomas S. Hofer[b], and Uwe Ruschewitz[a,*]

[a] *Department of Chemistry, University of Cologne, Greinstraße 6, D-50939 Cologne, Germany. E-mail: uwe.ruschewitz@uni-koeln.de*

[b] *Institute of General, Inorganic and Theoretical Chemistry, University of Innsbruck, Innrain 80-82, A-6020 Innsbruck, Austria. Email: Heidi.schwartz@uibk.ac.at*

Content

Figure S1. Emission spectra of 2-butyl-5,6-dimethoxyisoindoline-1,3-dione (= Phth) dissolved in solvents of varying polarity with λ_{ex} = 347 nm.

Figures S2 to S11. PXRD patterns of Phth@PM systems (**1 - 10**) in comparison to measured or simulated patterns of the unloaded porous material (PM).

Figure S12. *LeBail* fit of **4**.

Figures S13 to S22. XPS spectra of the Phth@PM systems (**1 - 10**) with fits of the characteristic core level and N 1s peak.

Figure S23. XPS spectra of the dilution series of Phth@MOF-5.

Figure S24. XPS spectra of the dilution series of Phth@MIL-68(Ga).

Figures S25 to S34. Emission and excitation spectra of the MOF host materials and Zeolite Y.

Figures S35 and S36. Time evolution of the nearest-neighbor distances between Phth and bdc groups of the $(Phth)_1$@MOF-5 and $(Phth)_3$@MOF-5 host systems.

Figures S37 and S38. Time evolution of the nearest-neighbor distances between Phth and bdc groups of the $(Phth)_1$@MIL-68(Ga) and $(Phth)_3$@MIL-68(Ga) host systems.

Table S1. Solvents used in this investigation with their elution power ε^0 according to *Snyder* and λ_{max} of 2-butyl-5,6-dimethoxyisoindoline-1,3-dione dissolved in these solvents.

Table S2. Results of the *Le Bail* fit of high-resolution synchrotron powder diffraction data of Phth@MIL-53(Al) (**4**) compared to the structural data of MIL-53(Al) *ht*.

Tables S3 to S4. Calculations of the composition of **1** to **10** *via* XPS.

Table S5. Ratio of Phth per formula unit of the PM in **1** to **10** as calculated from the XPS data.

Tables S6 to S7. Calculation of the composition of the dilution series of Phth@MOF-5 and Phth@MIL-68(Ga) *via* XPS.

References.

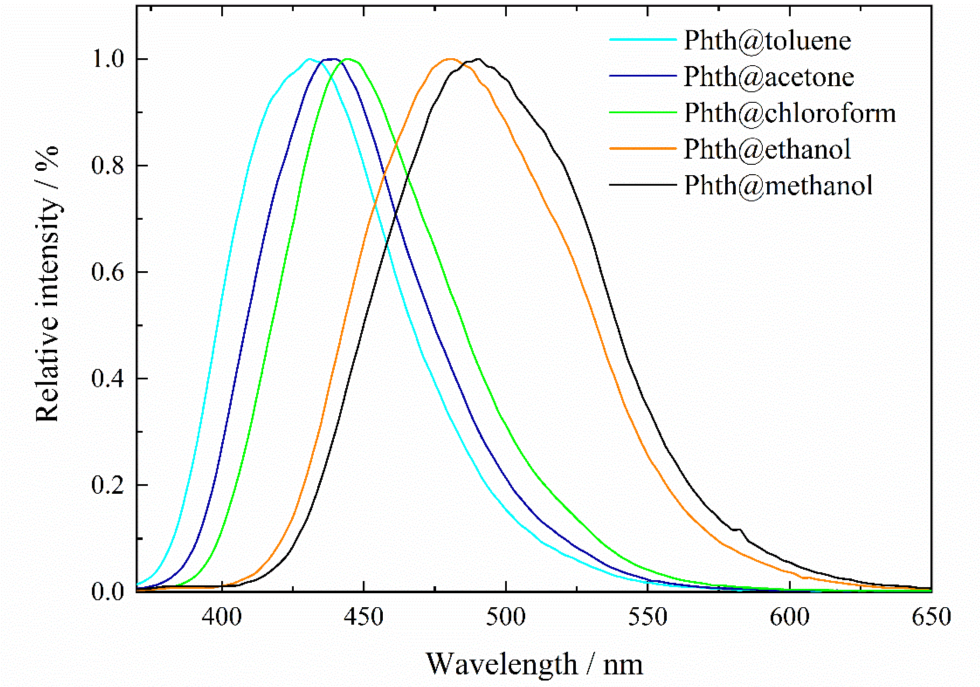

Figure S1. Normalized emission spectra of 2-butyl-5,6-dimethoxyisoindoline-1,3-dione (= Phth) dissolved in solvents of varying polarity with λ_{ex} = 347 nm.

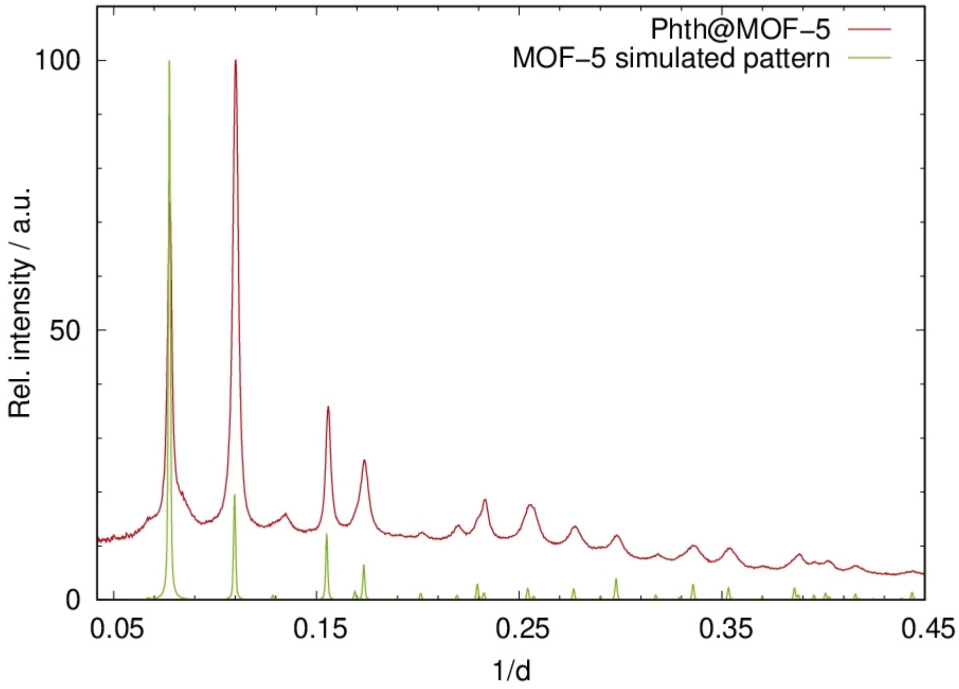

Figure S2. PXRD pattern of Phth@MOF-5 (**1**) (red), measured at 298 K (BL9/DELTA: λ = 0.49594 Å) in comparison to a simulated pattern of pristine MOF-5 (green).

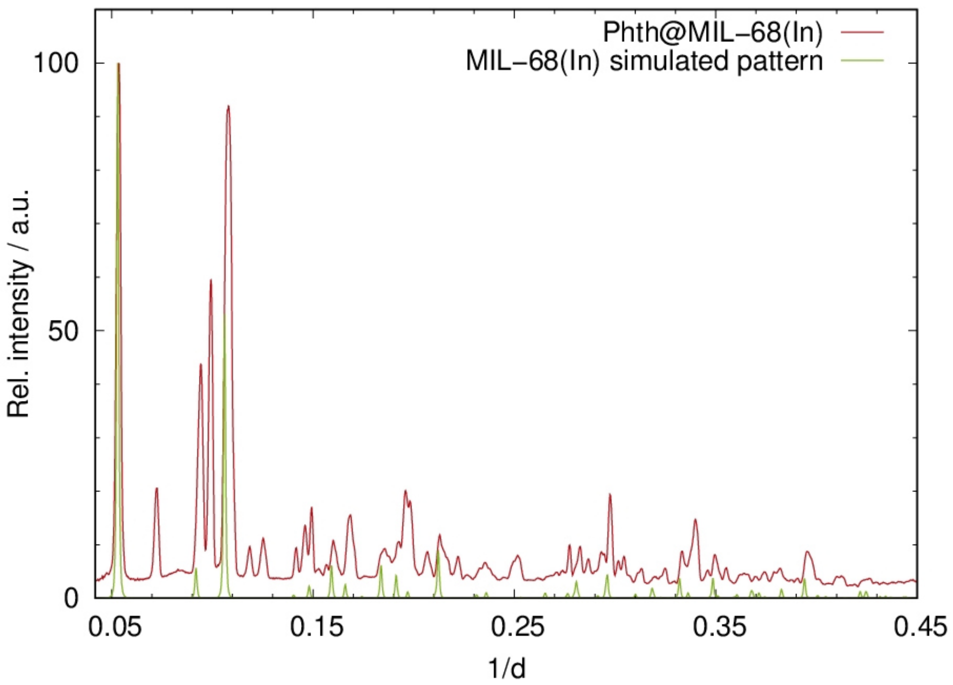

Figure S3. PXRD pattern of Phth@MIL-68(In) (**2**) (red), measured at 298 K (BL9/DELTA: $\lambda = 0.49594$ Å) in comparison to a simulated pattern of pristine MIL-68(In) *ht* (green).

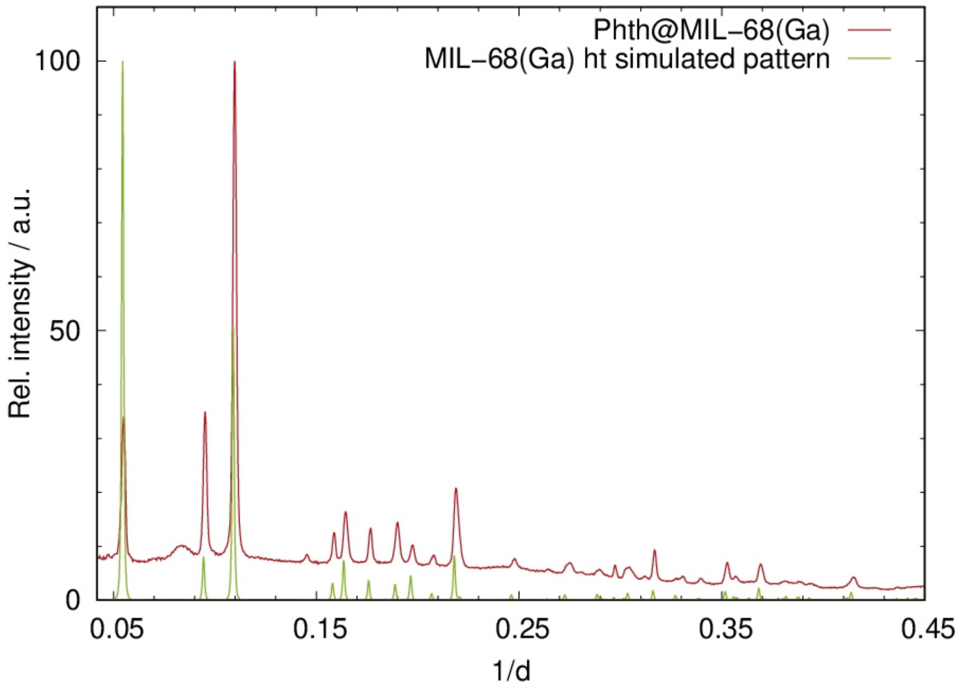

Figure S4. PXRD pattern of Phth@MIL-68(Ga) (**3**) (red), measured at 298 K (BL9/DELTA: $\lambda = 0.49594$ Å) in comparison to a simulated pattern of pristine MIL-68(Ga) *ht* (green).

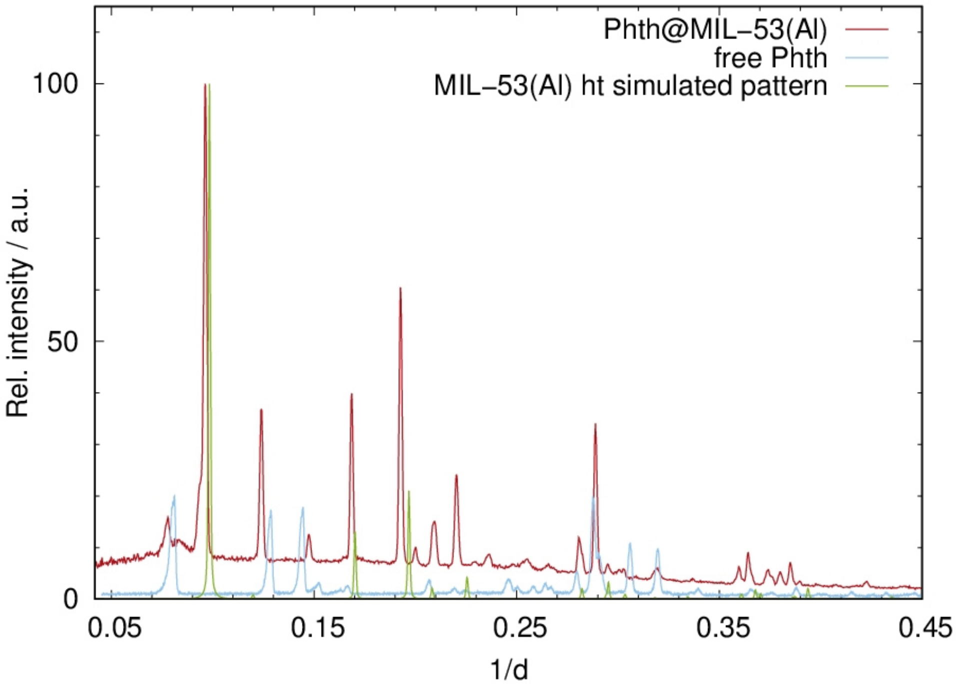

Figure S5. PXRD pattern of Phth@MIL-53(Al) (**4**) (red), measured at 298 K (BL9/DELTA: λ = 0.49594 Å) in comparison to a PXRD pattern of pristine Phth (blue), measured at 298 K (*Stoe StadiP*, *λ* = 1.54056 Å), and a simulated pattern of MIL-53(Al) *ht* (green).

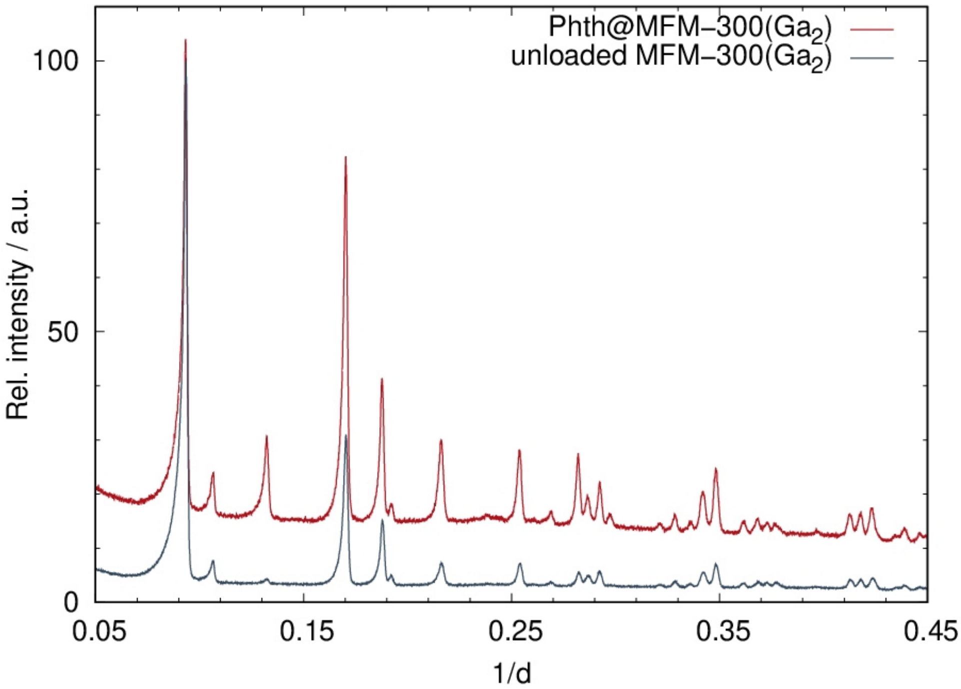

Figure S6. PXRD patterns of Phth@MFM-300(Ga_2) (**5**) (red) in comparison to pristine MFM-300(Ga_2) (blue-grey), both measured at 298 K (*Huber G670*: λ = 1.54056 Å).

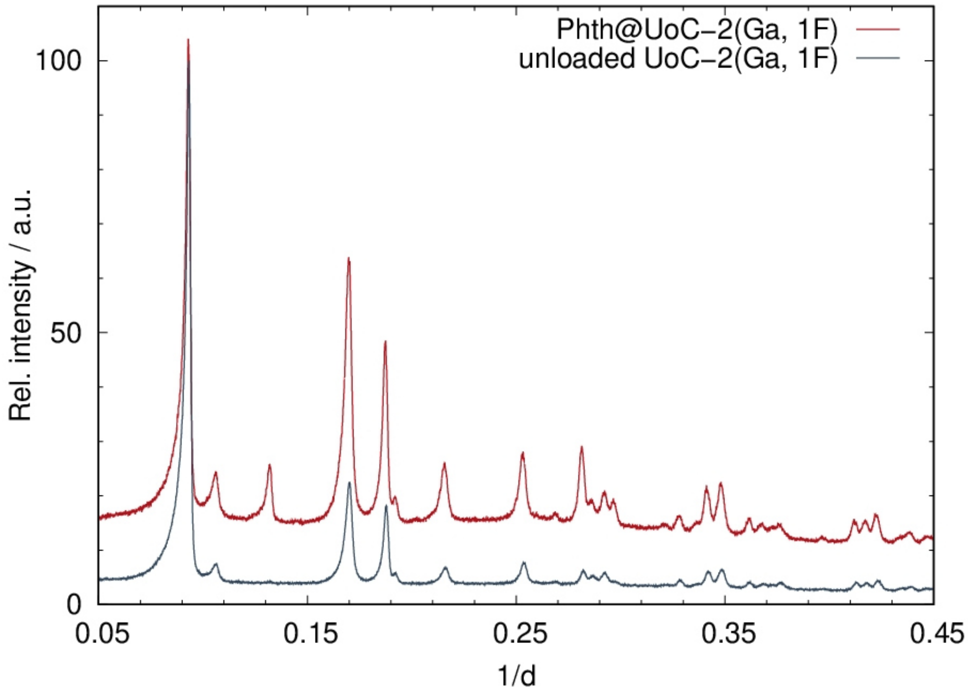

Figure S7. PXRD patterns of Phth@UoC-2(Ga,1F) (**6**) (red) in comparison to pristine UoC-2(Ga,1F) (blue-grey), both measured at 298 K (*Huber G670*: λ = 1.54056 Å).

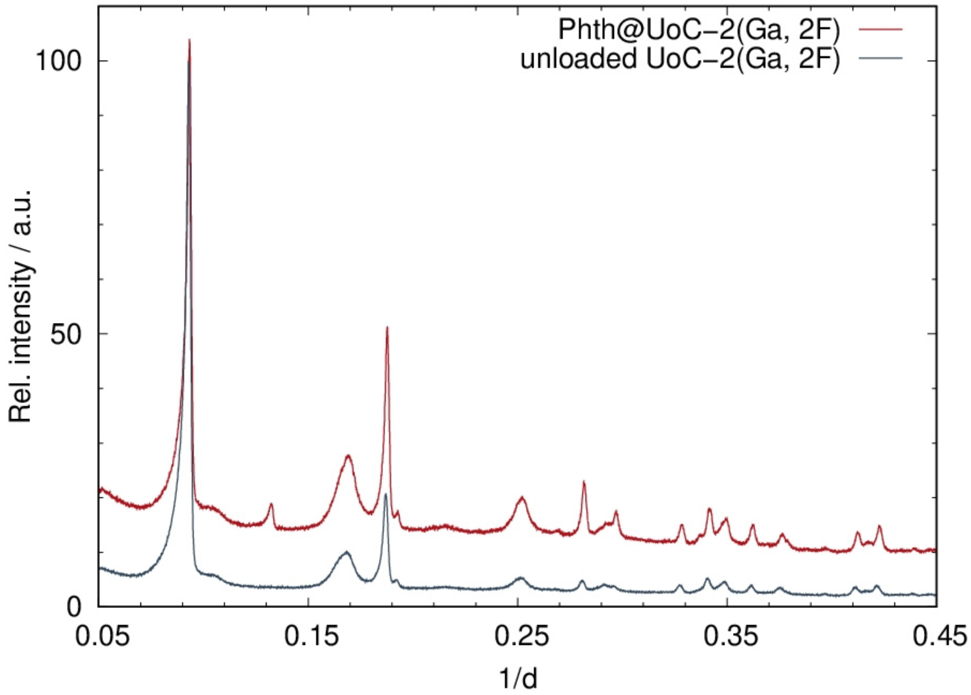

Figure S8. PXRD patterns of Phth@UoC-2(Ga,2F) (**7**) (red) in comparison to pristine UoC-2(Ga,2F) (blue-grey), both measured at 298 K (*Huber G670*: λ = 1.54056 Å).

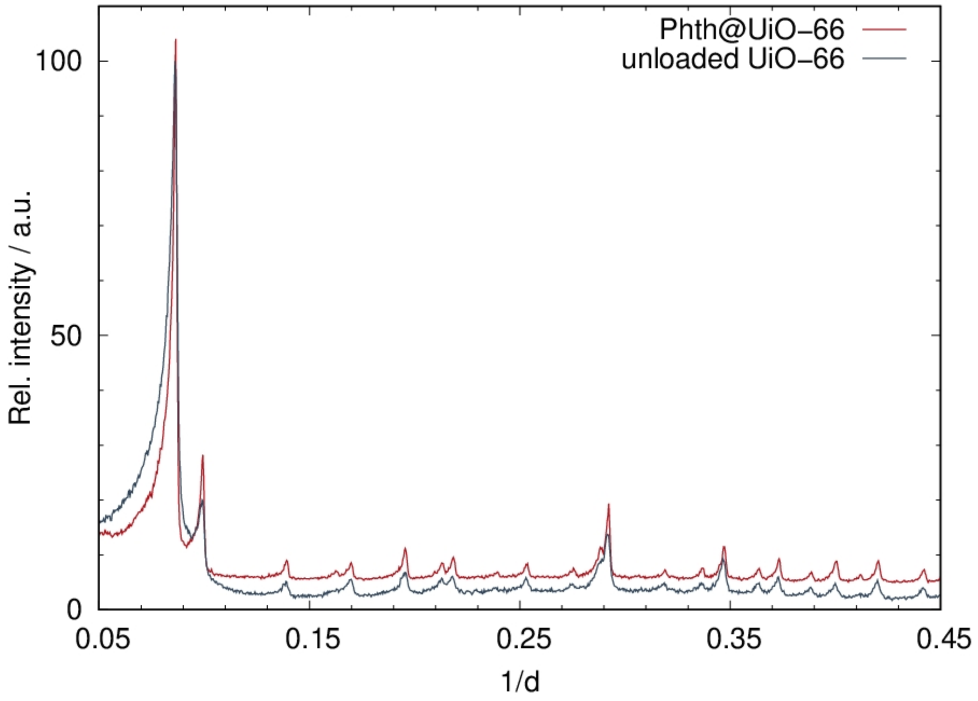

Figure S9. PXRD patterns of Phth@UiO-66 (**8**) (red) in comparison to pristine UiO-66 (blue-grey), both measured at 298 K (*Stoe StadiP*: λ = 0.7093 Å).

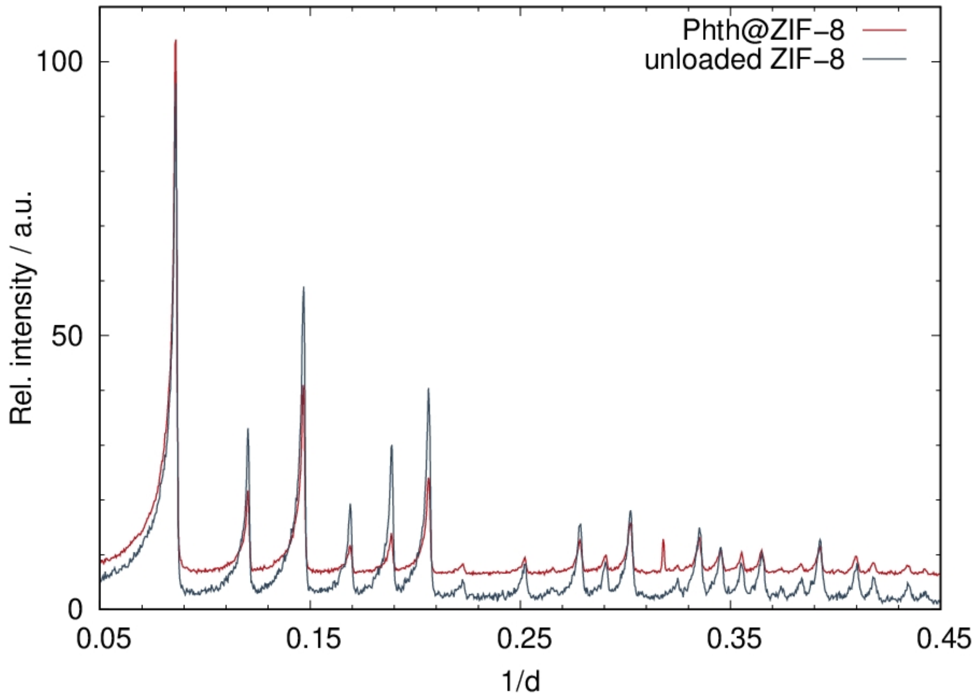

Figure S10. PXRD patterns of Phth@ZIF-8 (**9**) (red) in comparison to pristine ZIF-8 (blue-grey), both measured at 298 K (*Stoe StadiP*: λ = 0.7093 Å).

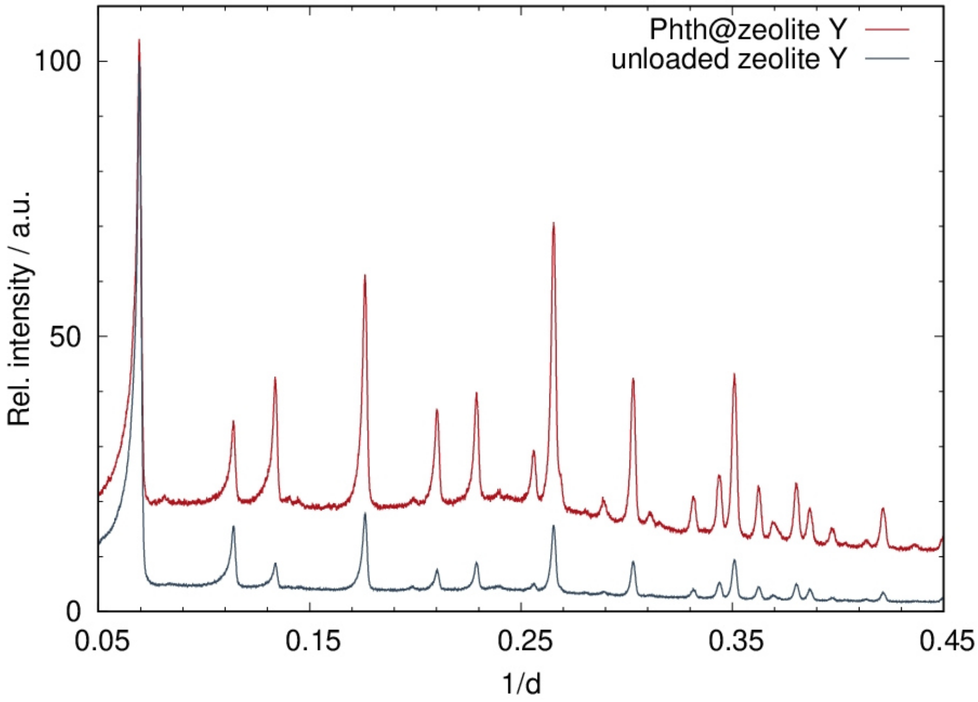

Figure S11. PXRD patterns of Phth@zeolite Y (**10**) (red) in comparison to pristine Zeolite Y (blue-grey), both measured at 298 K (*Huber G670*: λ = 1.54056 Å).

For MIL-53(Al), a change of the diffraction pattern was expected upon guest loading due to the known "breathing effect" of the host material. When trying to upload larger amounts of spiropyrans or spirooxazines as guest molecules, no additional peaks or changes in the diffraction patterns were observed. Since photochromic behavior of the respective dye was still observed, surface adsorption of the photoswitchable molecule as an amorphous film was assumed and confirmed by means of XPS measurements.[1,2] In contrast, for Phth@MIL-53(Al) (**4**), a modulated diffraction pattern is found compared to MIL-53(Al) *ht* with minor amounts of solid (crystalline) Phth being present (Figure S5). After excluding these reflections, the unit cell and possible space group of **4** was determined with the program package *Topas*[3] and fitted with *JANA2006*[4]. The results are listed in Table S2 and the *Le Bail* fit is shown in Figure S12. Guest-free MIL-53(Al) *ht* crystallizes in the space group *Imma* (no. 74) with a unit cell volume of 1411.95 Å^3.[5] Upon loading with Phth, the unit cell volume of Phth@MIL-53(Al) (**4**) increases to 1445.5 Å^3. The symmetry (i.e. space group *Imma*, no. 74) does not change, but the lattice parameters *b* and *c* (the open pores are aligned parallel to *c*) change significantly (cp. Table S2).

For **2**, (Figure S3), additional reflections *e.g.* in the low angle region are the result of non-embedded Phth, which is assumed to arrange itself in an ordered fashion on the surface of the

MOF host. This assumption is corroborated by the XPS measurements, where an additional broad feature is present at ~ 397 eV (see Figure S14). Even after heating the sample to high temperatures (150 °C), the additional reflections in the PXRD pattern remained.

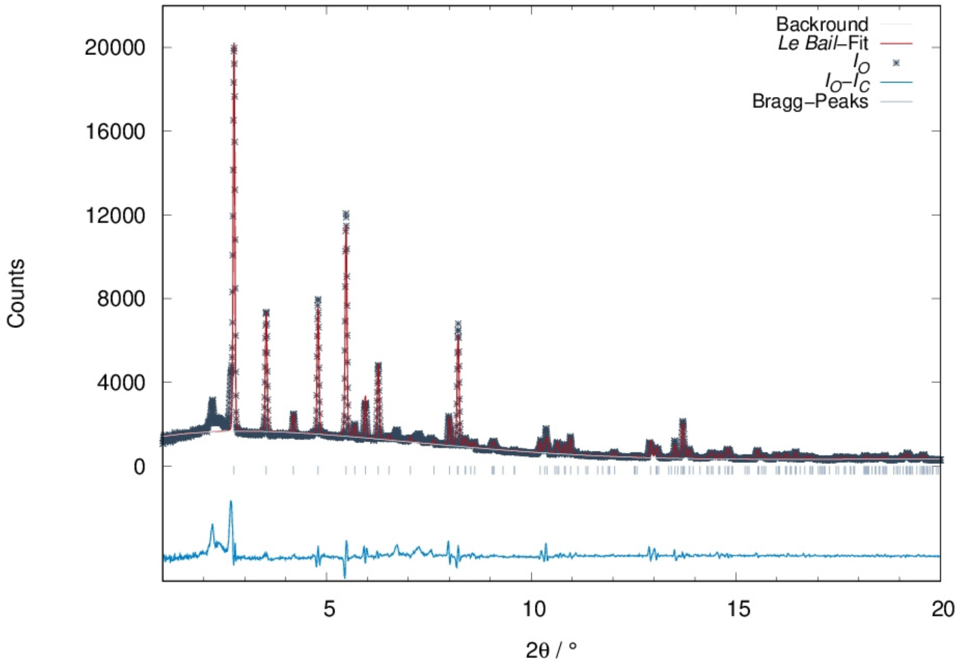

Figure S12. *Le Bail* fit of Phth@MIL-53(Al) (**4**) (298 K, BL9/DELTA: λ = 0.49594 Å) with measured intensities (dark grey, crosses), calculated intensities (red line), differential curve (turquoise), *Bragg* peaks (light blue, ticks) and background of refinement (white line). The strongest reflection of Phth is present at an angle of ~ 1.5 ° *2θ*.

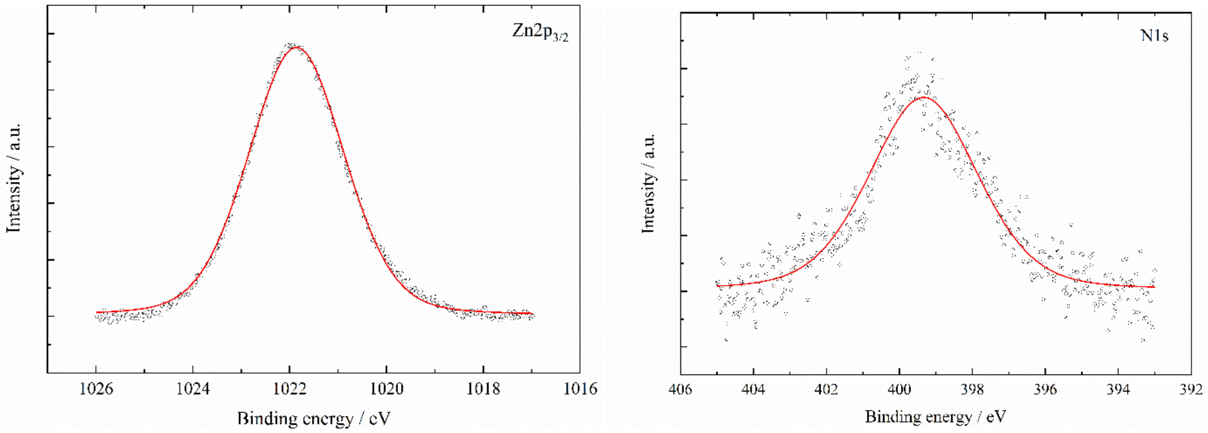

Figure S13. XPS spectrum of the Zn 2p$_{3/2}$ (left) and N 1s peak (right) of Phth@MOF-5 (**1**) with experimental data (black dots) and fit (red line).

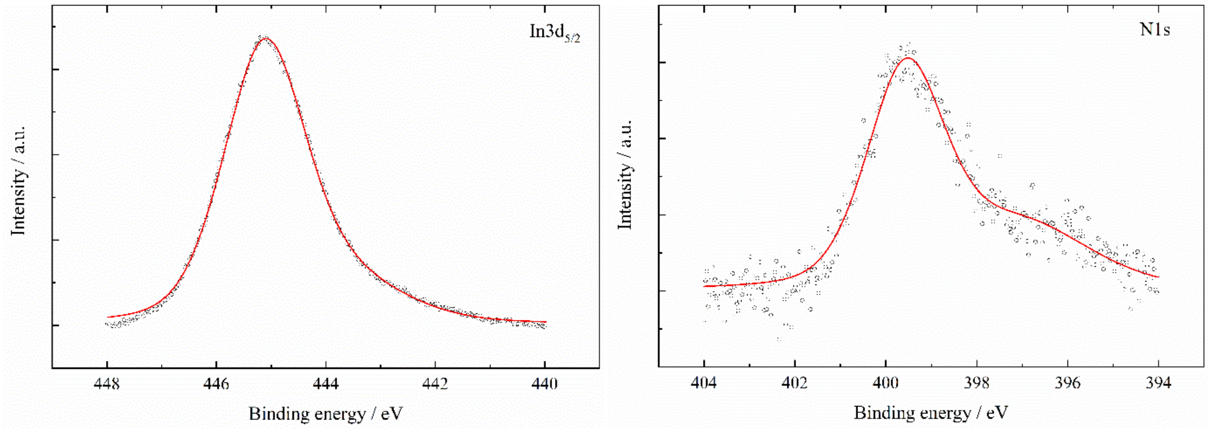

Figure S14. XPS spectrum of the In $3d_{5/2}$ (left) and N 1s peak (right) of Phth@MIL-68(In) (**2**) with experimental data (black dots) and fit (red line).

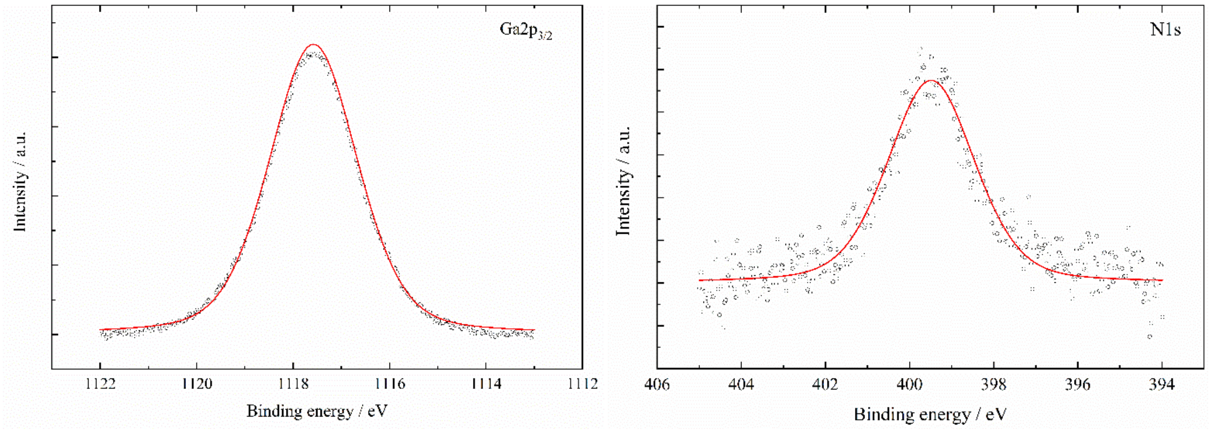

Figure S15. XPS spectrum of the Ga $2p_{3/2}$ (left) and N 1s peak (right) of Phth@MIL-68(Ga) (**3**) with experimental data (black dots) and fit (red line).

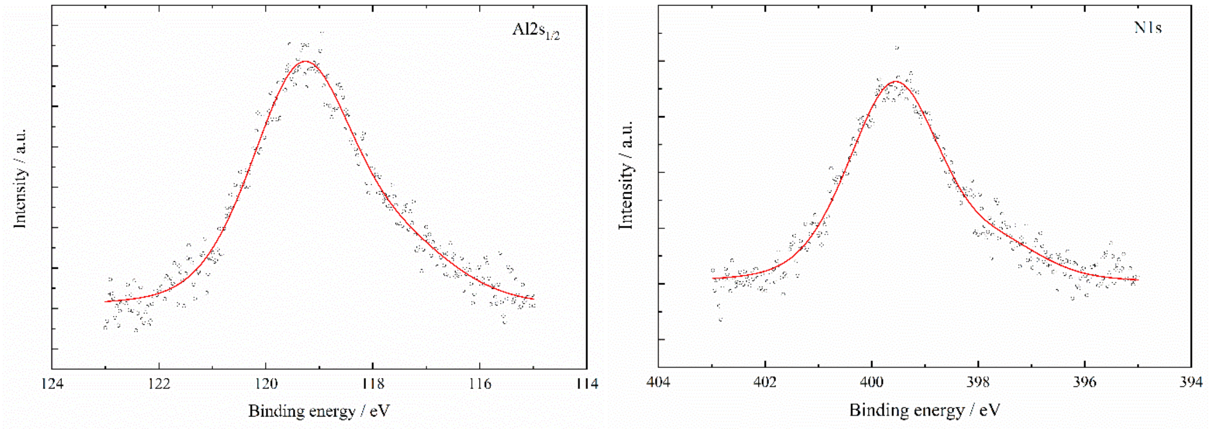

Figure S16. XPS spectrum of the Al 2s (left) and N 1s peak (right) of Phth@MIL-53(Al) (**4**) with experimental data (black dots) and fit (red line).

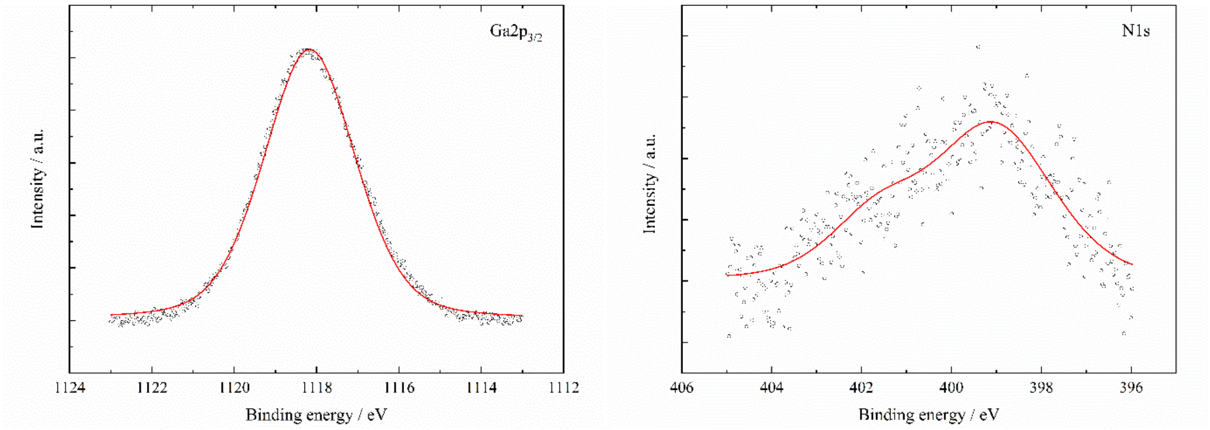

Figure S17. XPS spectrum of the Ga $2p_{3/2}$ (left) and N 1s peak (right) of Phth@MFM-300(Ga_2) (**5**) with experimental data (black dots) and fit (red line).

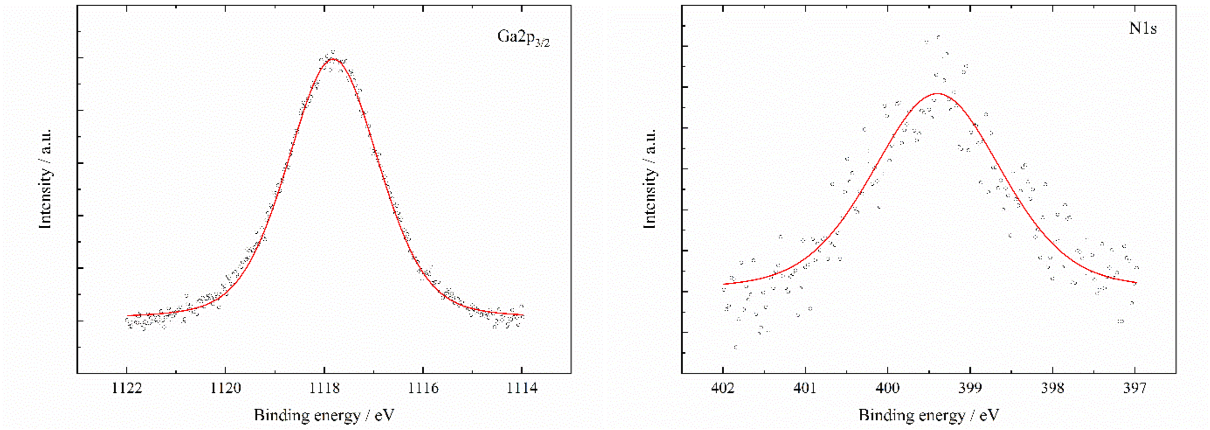

Figure S18. XPS spectrum of the Ga $2p_{3/2}$ (left) and N 1s peak (right) of Phth@UoC-2(Ga,1F) (**6**) with experimental data (black dots) and fit (red line).

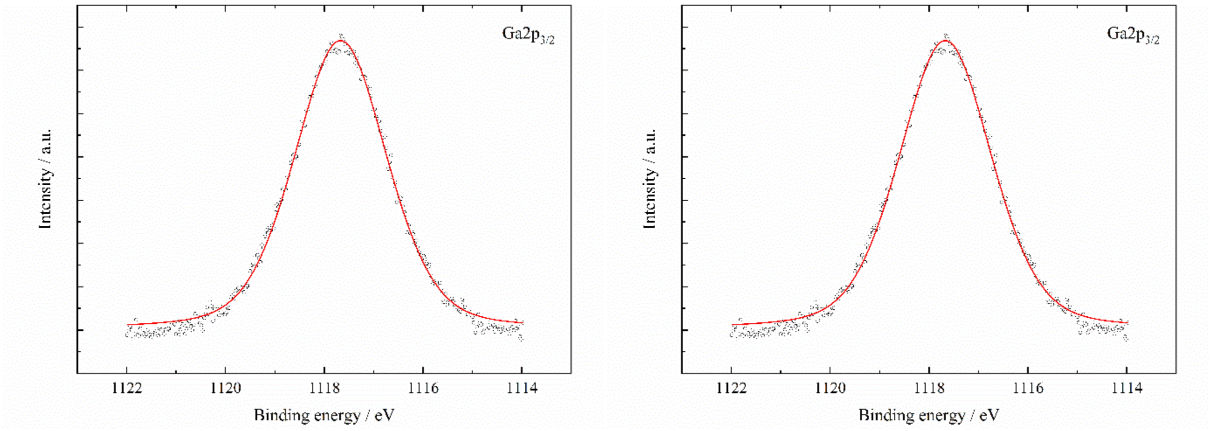

Figure S19. XPS spectrum of the Ga $2p_{3/2}$ (left) and N 1s peak (right) of Phth@UoC-2(Ga,2F) (**7**) with experimental data (black dots) and fit (red line).

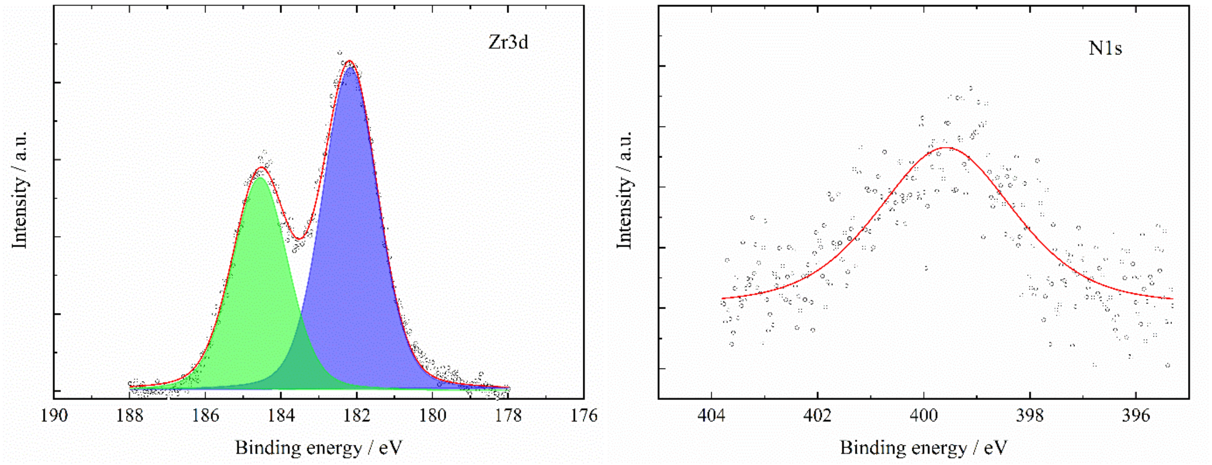

Figure S20. XPS spectrum of the Zr $3d_{5/2}$ (left, blue area), Zr $3d_{3/2}$ (left, green area) and N 1s peak (right) of Phth@UiO-66 (**8**) with experimental data (black dots) and fit (red line).

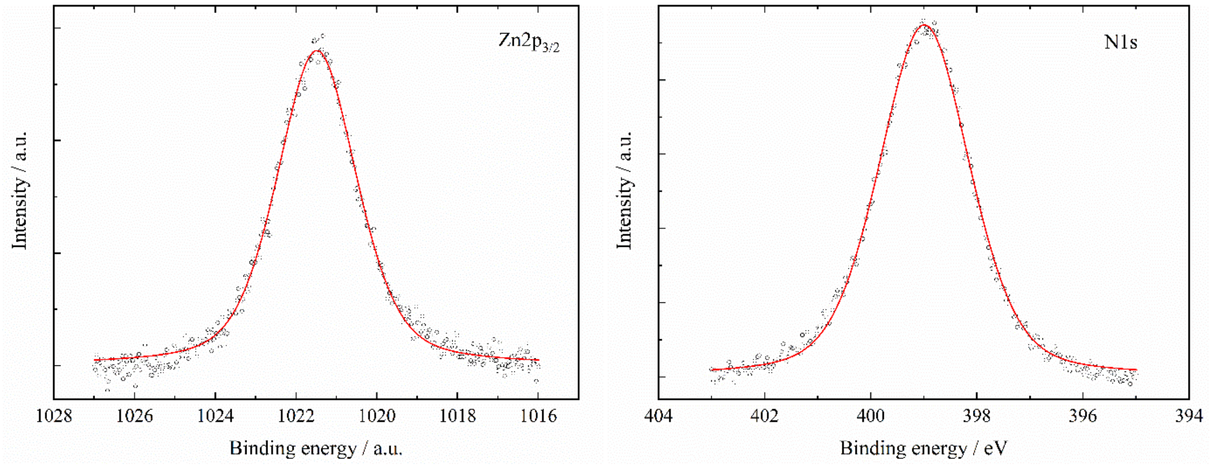

Figure S21. XPS spectrum of the Zn $2p_{3/2}$ (left) and N 1s peak (right) of Phth@ZIF-8 (**9**) with experimental data (black dots) and fit (red line).

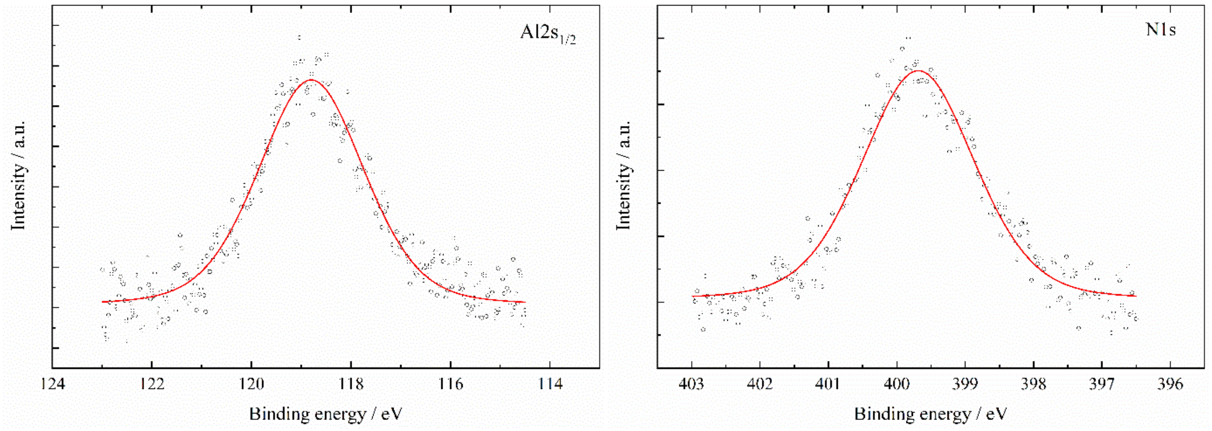

Figure S22. XPS spectrum of Al 2s (left) and N 1s peak (right) of Phth@zeolite Y (**10**) with experimental data (black dots) and fit (red line).

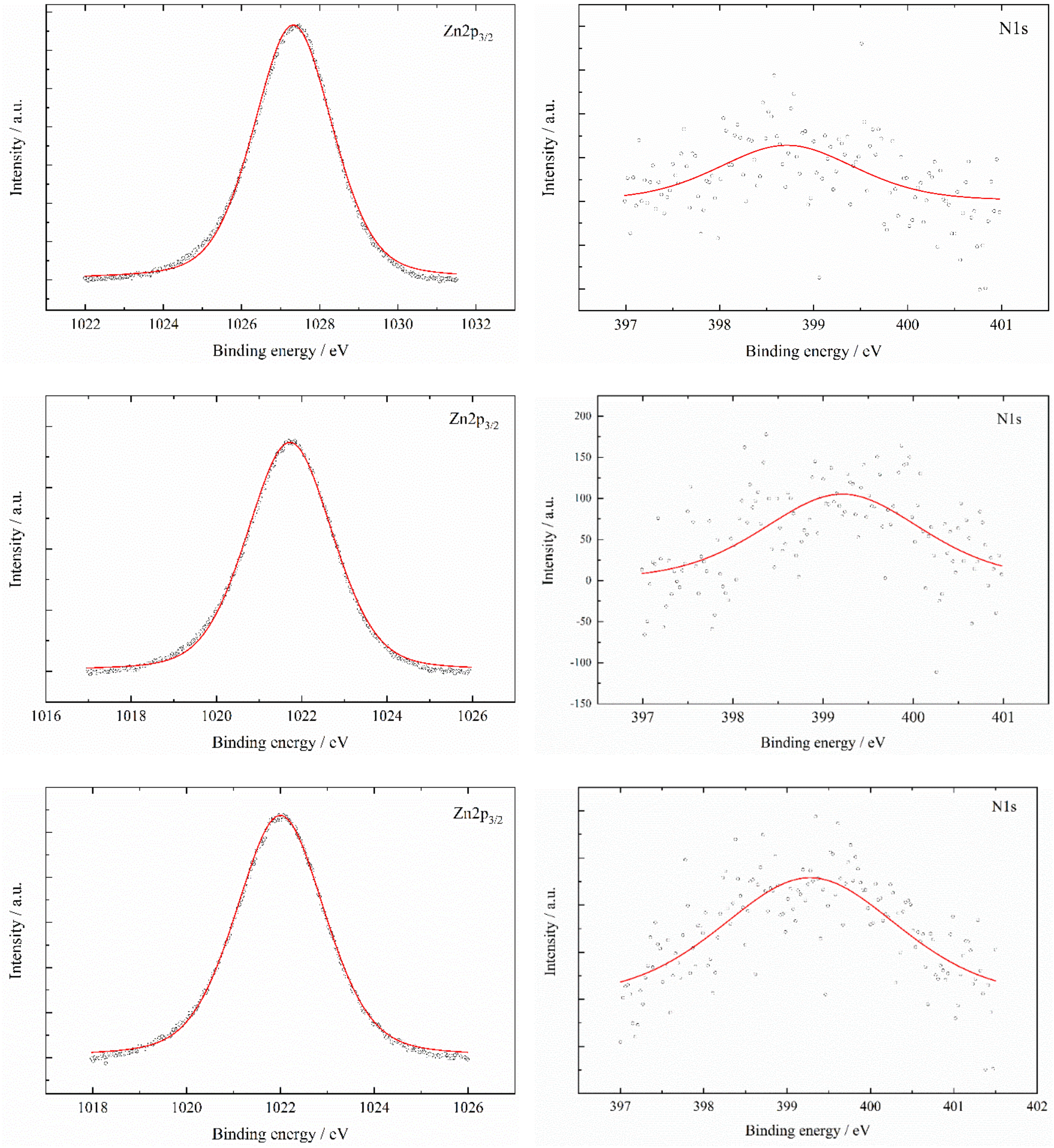

Figure S23. XPS spectra of the Zn 2p3/2 (left) and N 1s peak (right) of Phth@MOF-5 with molar guest-to-host ratios set to 0.1:1 (top), 0.25:1 (center), and 0.5:1 (bottom) with experimental data (black dots) and fit (red line).

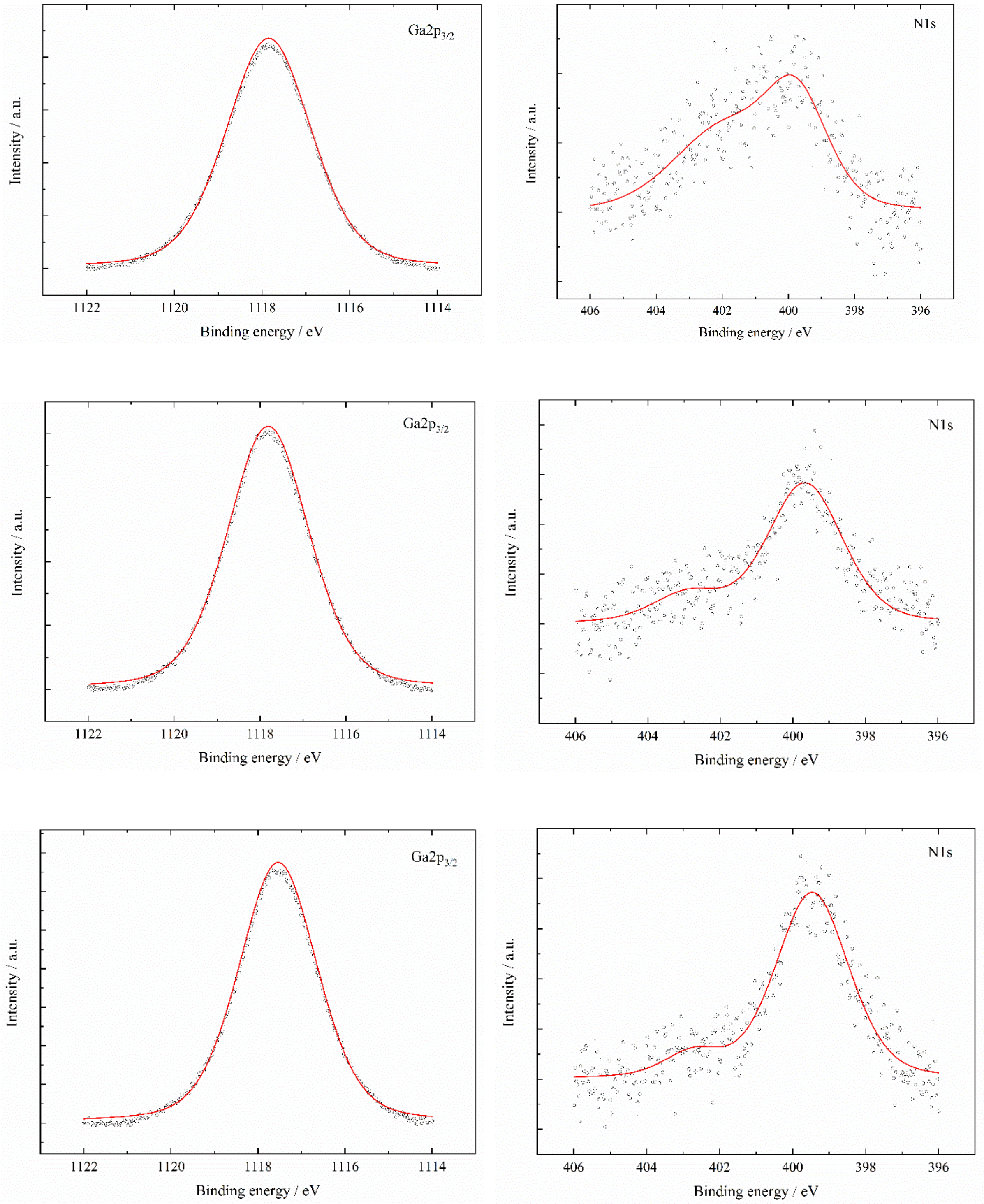

Figure S24. XPS spectra of the Ga 2p3/2 (left) and N 1s peak (right) of Phth@MIL-68(Ga) with molar guest-to-host ratios set to 0.1:1 (top), 0.25:1 (center), and 0.5:1 (bottom) with experimental data (black dots) and fit (red line).

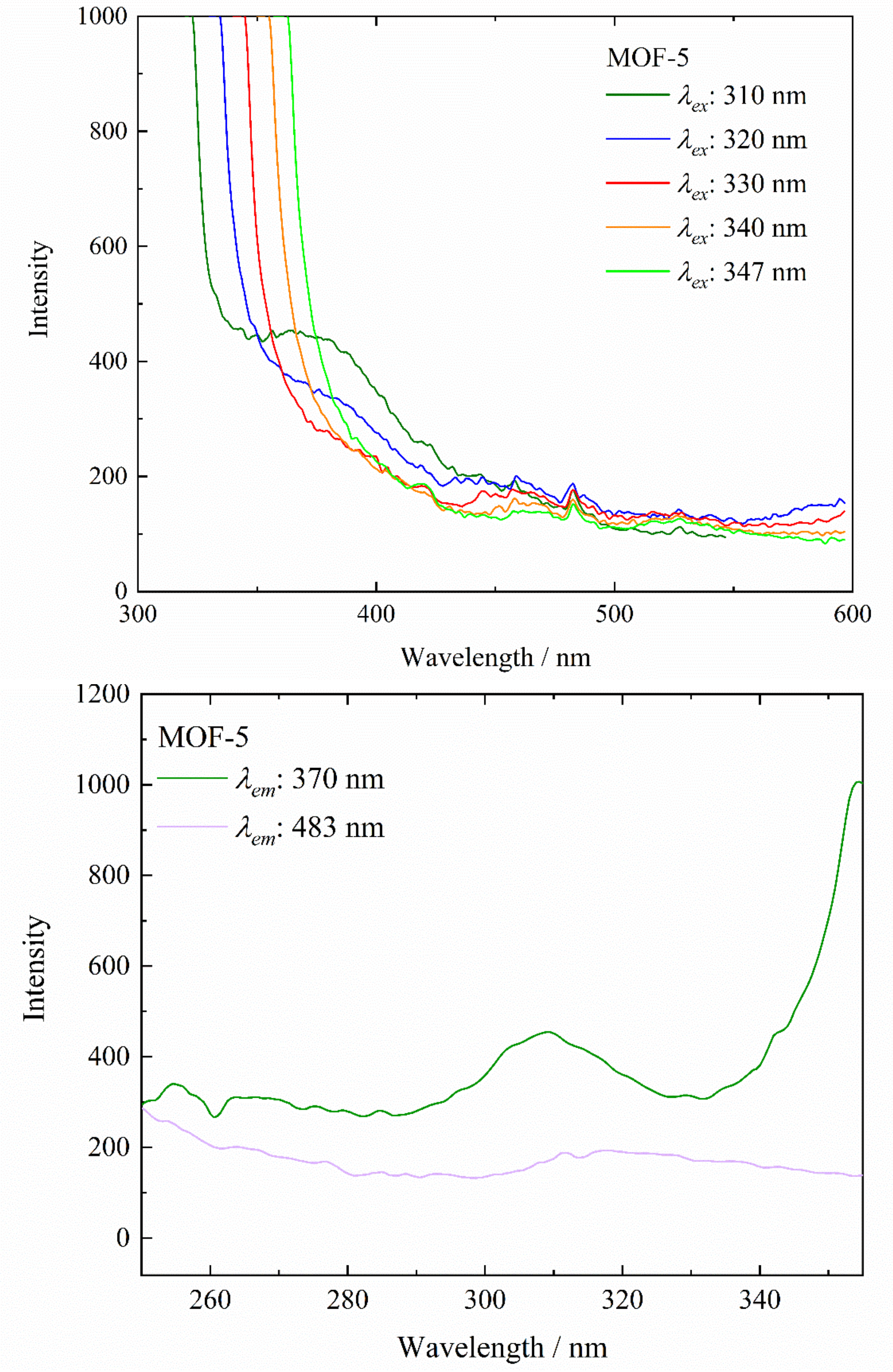

Figure S25. Emission (top) and excitation (bottom) spectra of MOF-5 with respective excitation and emission wavelengths given within the figure.

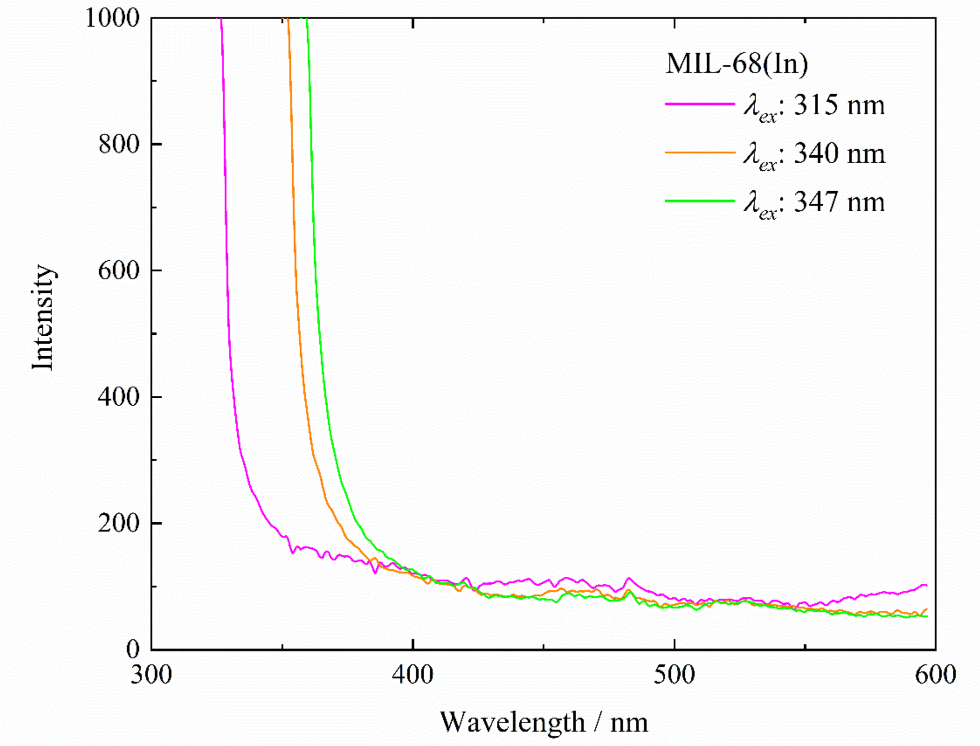

Figure S26. Emission spectra of MIL-68(In) with respective excitation wavelengths given within the figure.

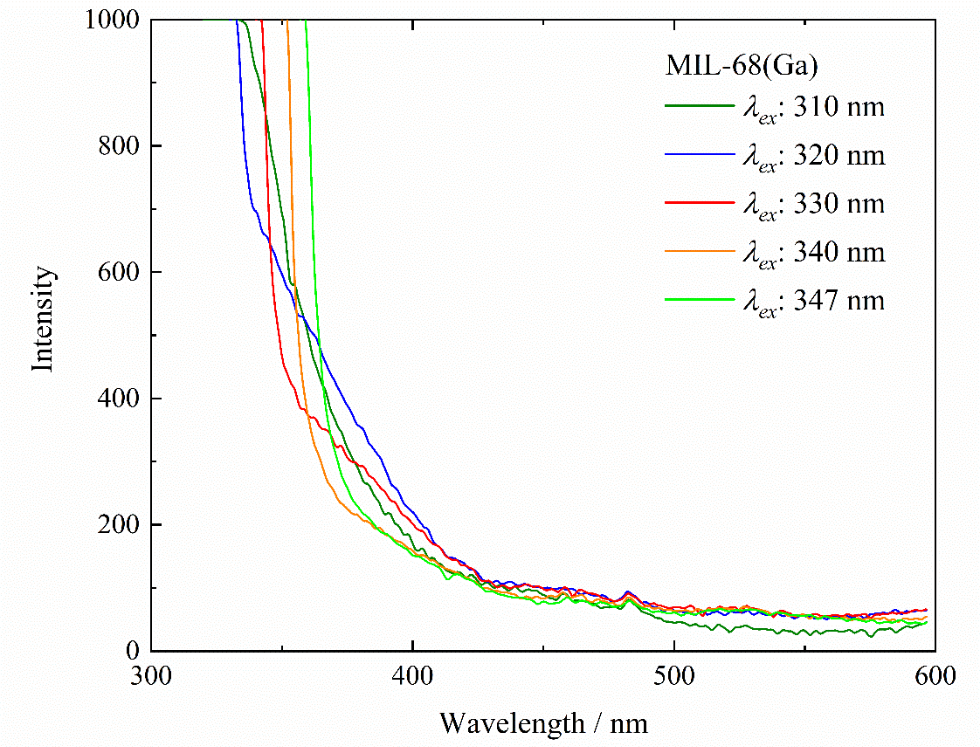

Figure S27. Emission spectra of MIL-68(Ga) with respective excitation wavelengths given within the figure.

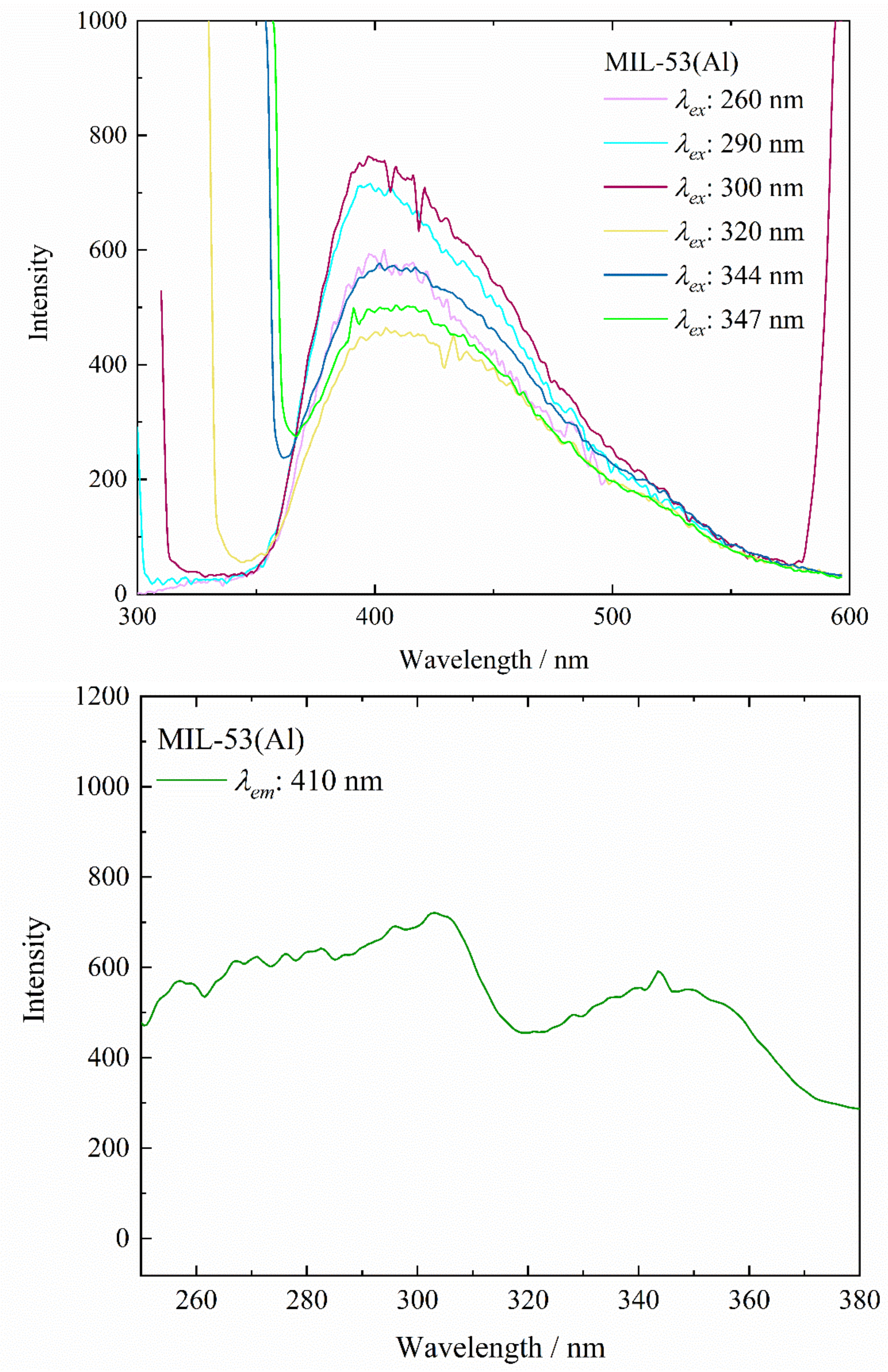

Figure S28. Emission (top) and excitation (bottom) spectra of MIL-53(Al) with respective excitation and emission wavelengths given within the figure.

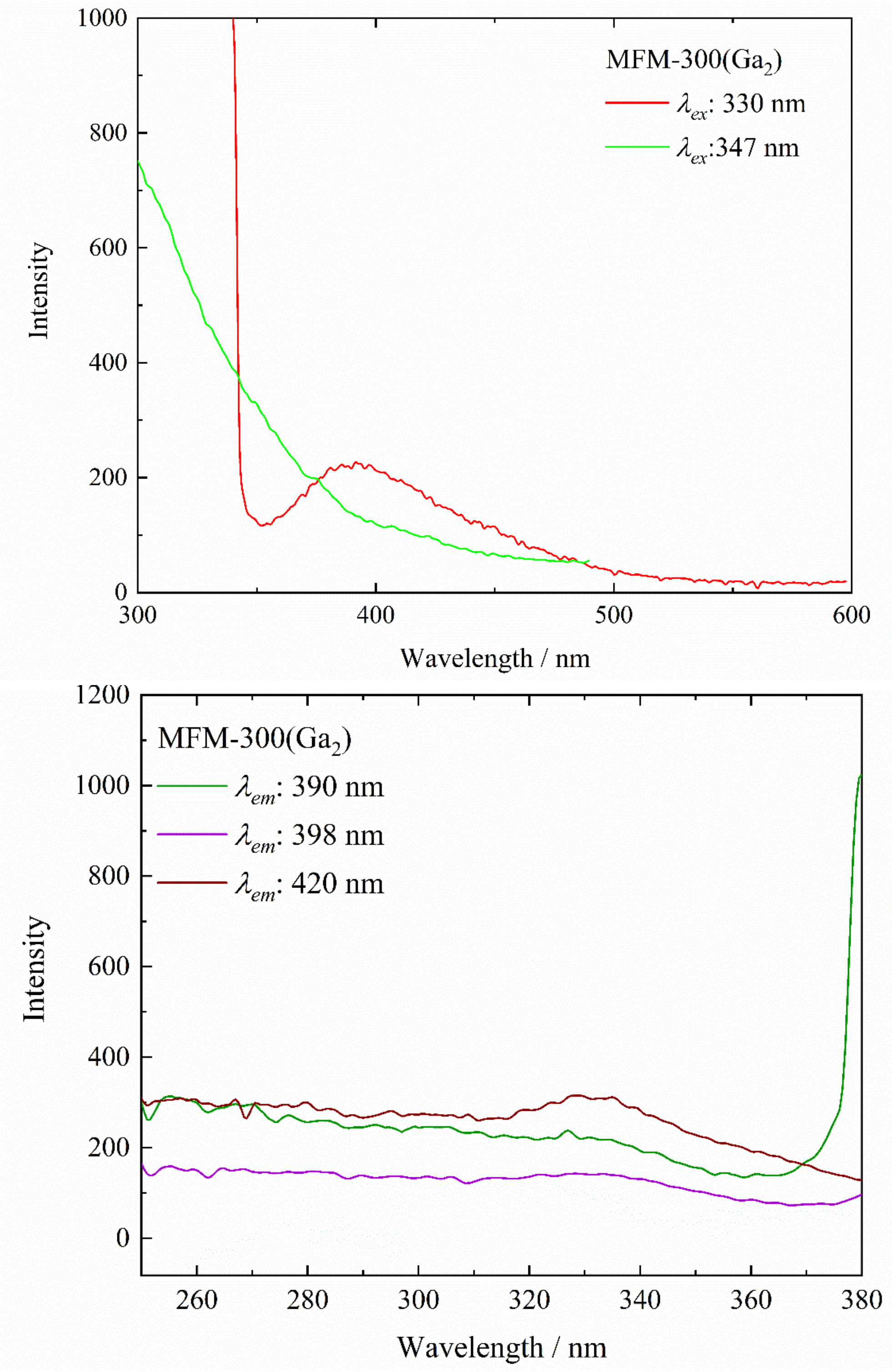

Figure S29. Emission (top) and excitation (bottom) spectra of MFM-300(Ga_2) with respective excitation and emission wavelengths given within the figure.

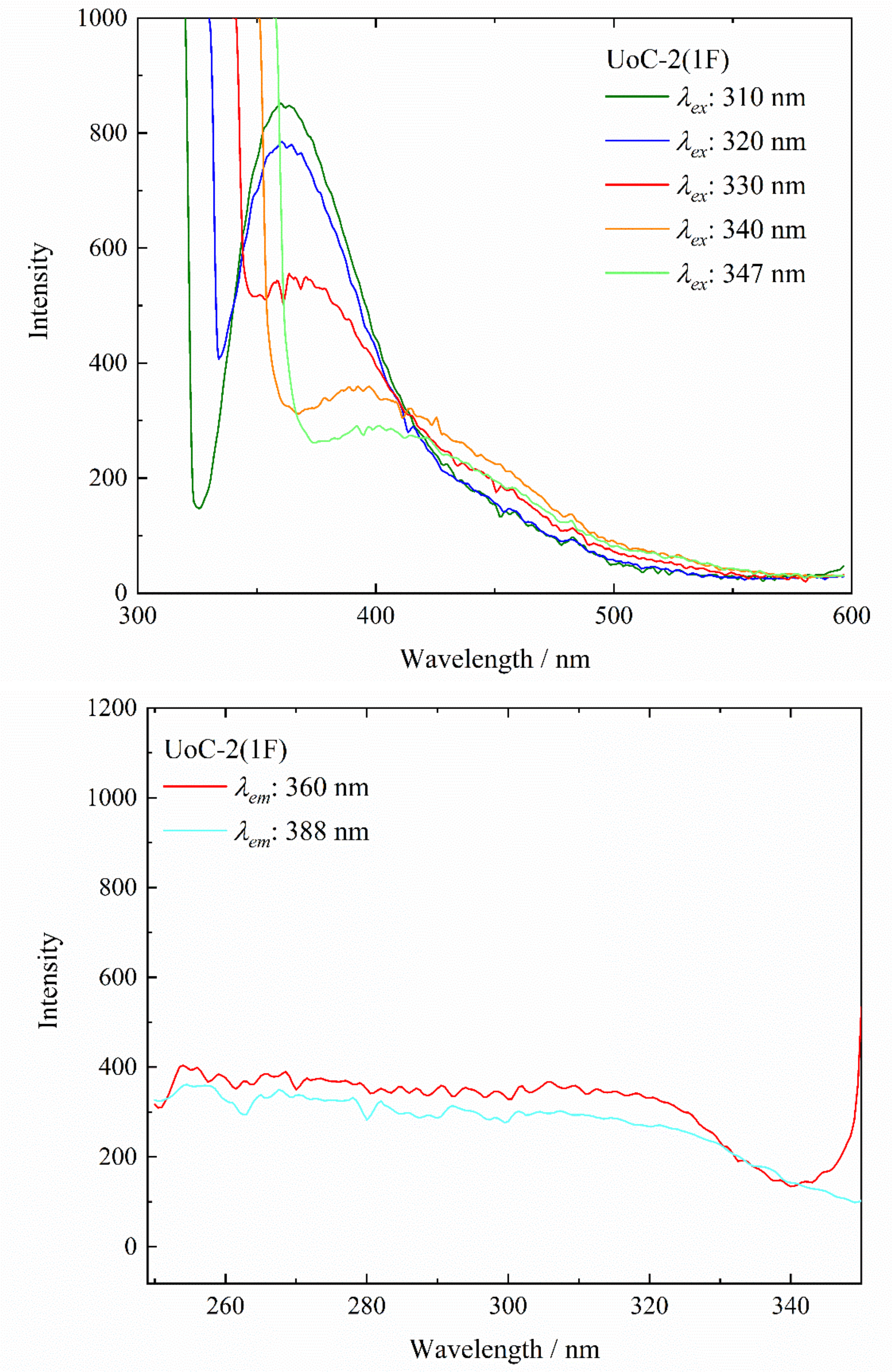

Figure S30. Emission (top) and excitation (bottom) spectra of UoC-2(1F) with respective excitation and emission wavelengths given within the figure.

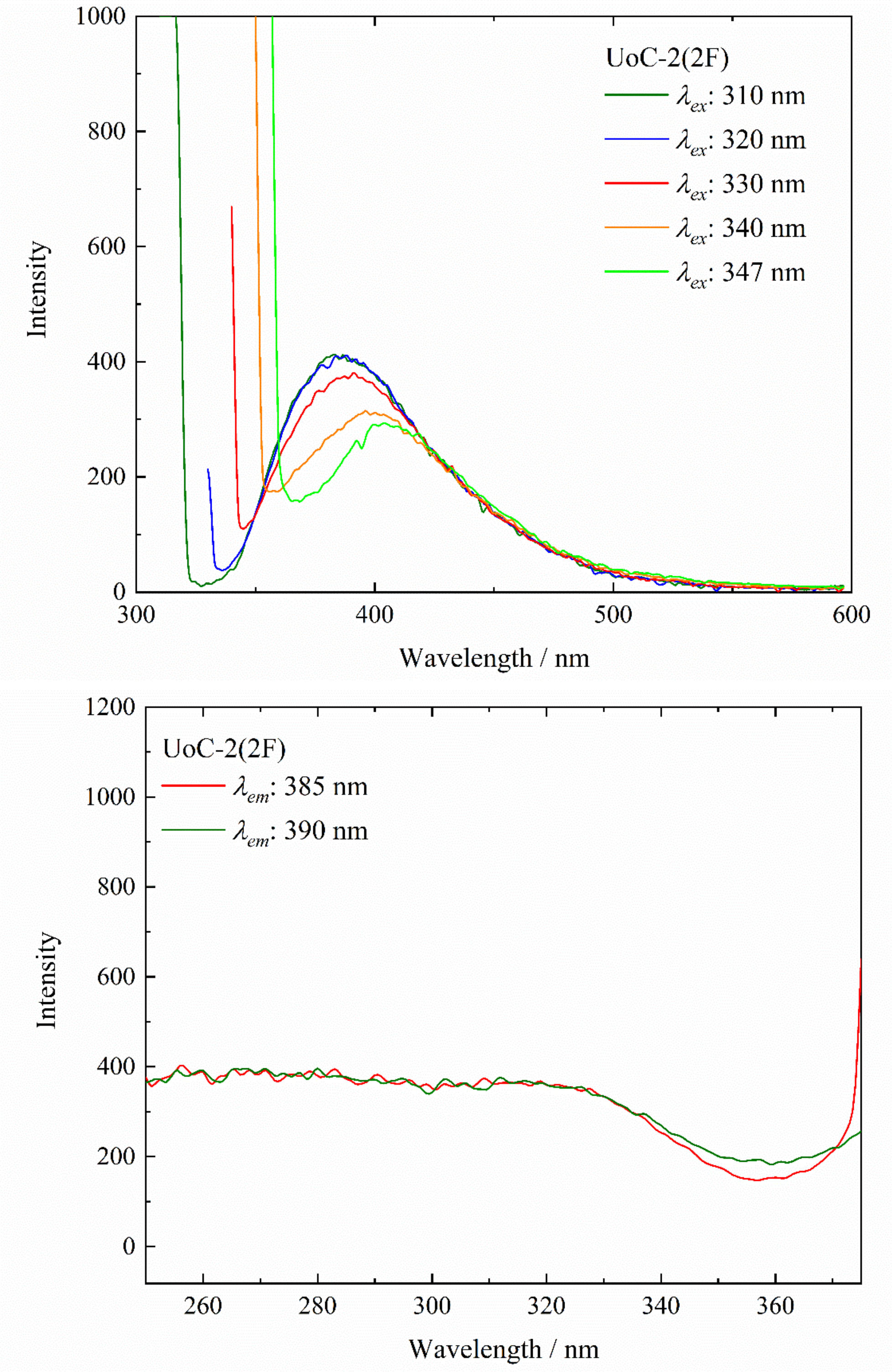

Figure S31. Emission (top) and excitation (bottom) spectra of UoC-2(2F) with respective excitation and emission wavelengths given within the figure.

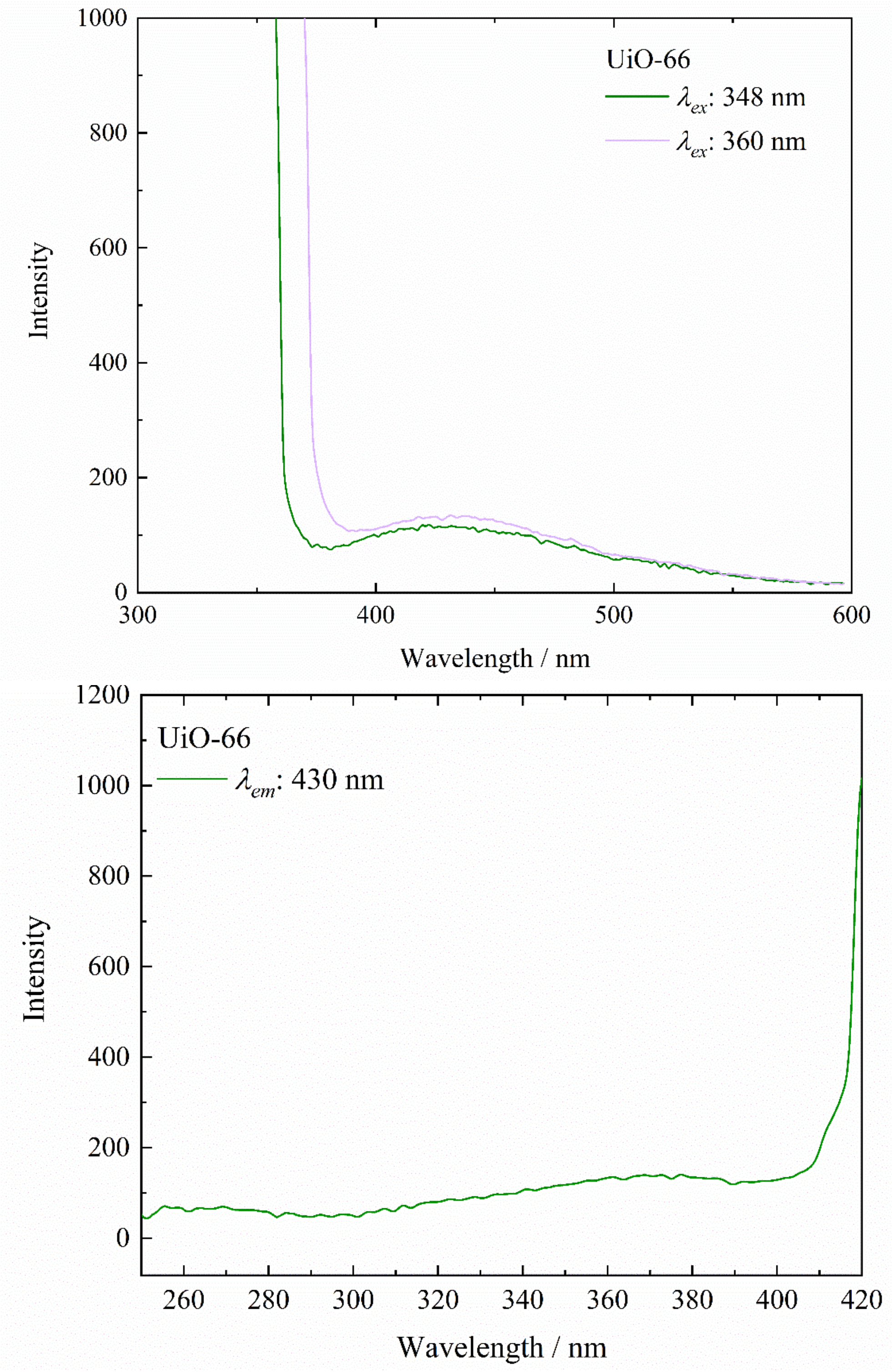

Figure S32. Emission (top) and excitation (bottom) spectra of UiO-66 with respective excitation and emission wavelengths given within the figure.

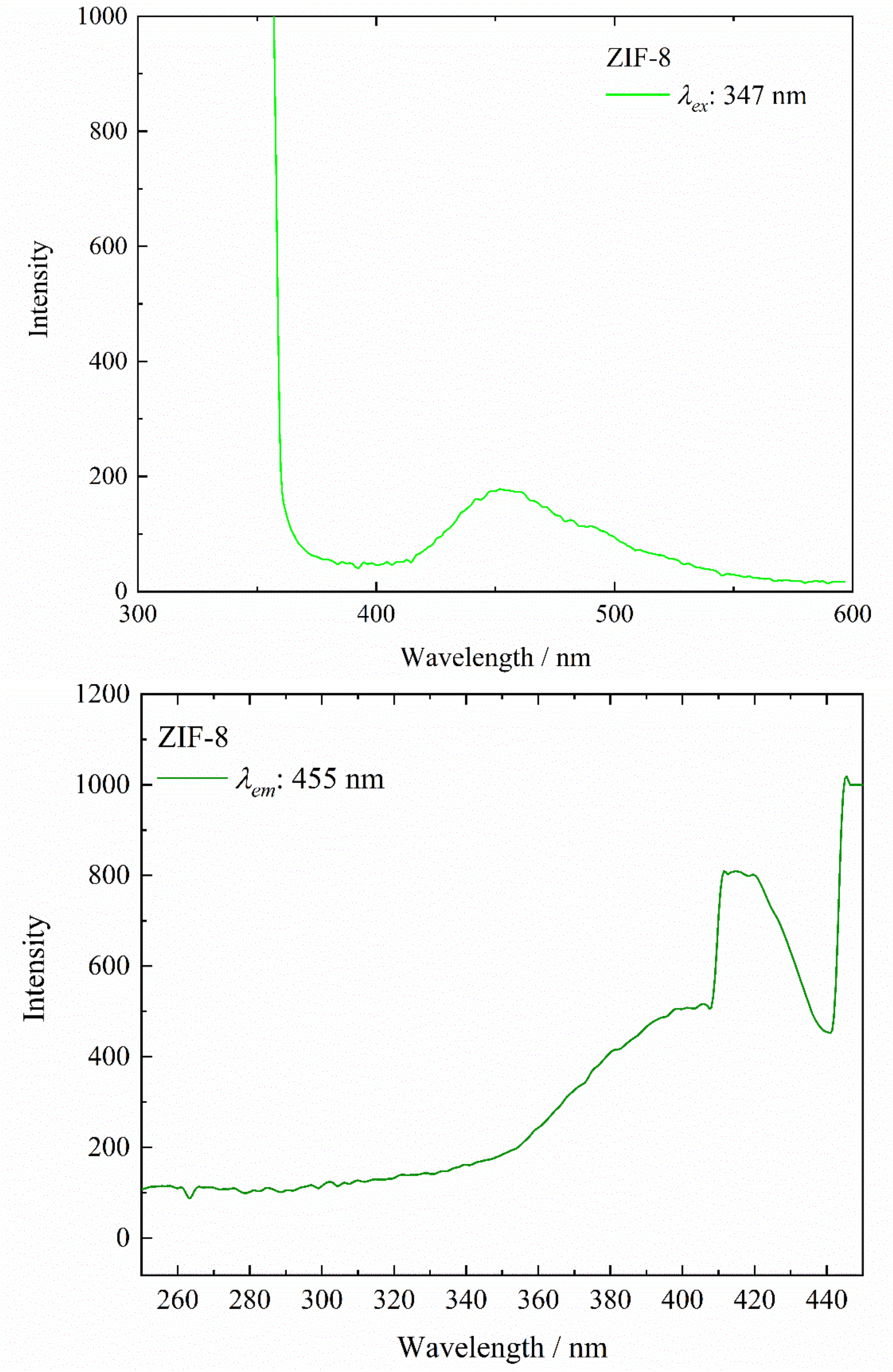

Figure S33. Emission (top) and excitation (bottom) spectra of ZIF-8 with respective excitation and emission wavelengths given within the figure.

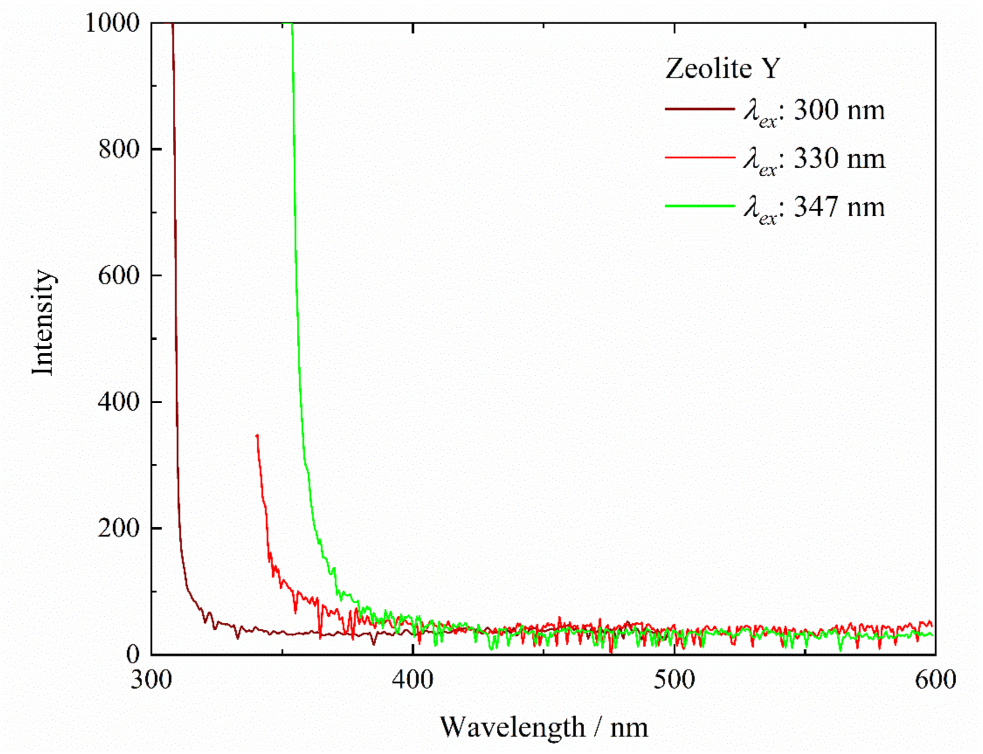

Figure S34. Emission spectra of Zeolite Y with respective excitation wavelengths given within the figure.

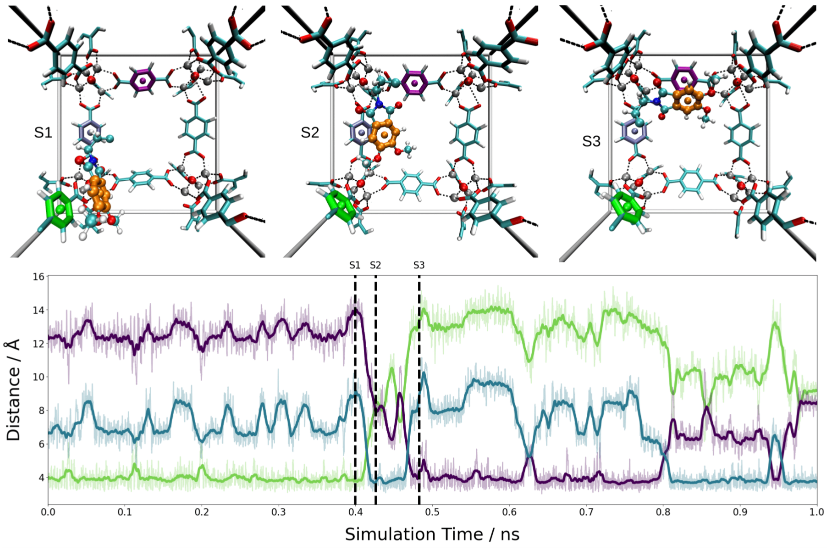

Figure S35. Time evolution of the nearest-neighbor distances between Phth and bdc groups of the $Phth_1$@MOF-5 host system analyzed based on the centroids of the aromatic units. The latter were determined as an average over the respective carbon atoms. Snapshots displaying the key configurations encountered along the DFTB MD simulations are marked S1, S2 and S3. The colors of the nearest-neighbor distances (bottom, purple, blue and green lines) reflect the distances between the inserted Phth to the similarly colored phenyl-rings of the bdc linkers (top, purple, blue and green colored phenyl rings).

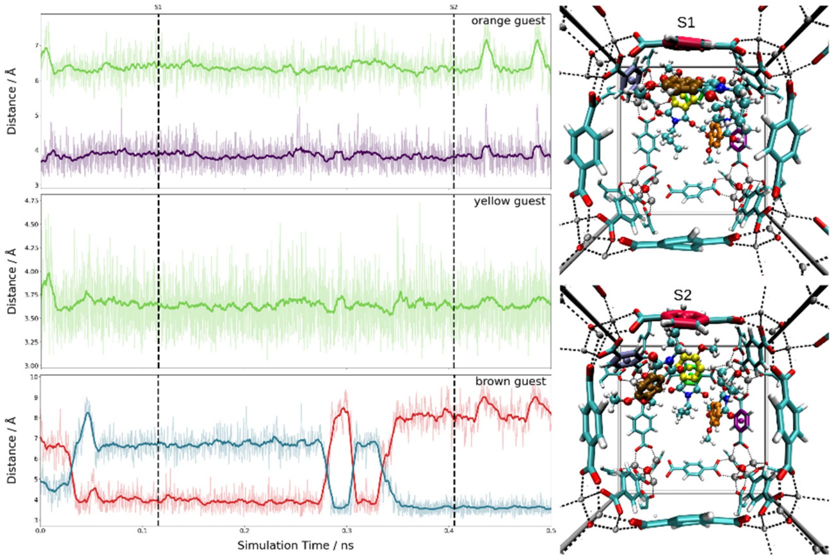

Figure S36. Time evolution of the nearest-neighbor distances between Phth and bdc groups of the $Phth_3$@MOF-5 host system analyzed based on the centroids of the aromatic units. The latter were determined as an average over the respective carbon atoms. Snapshots displaying the key configurations encountered along the DFTB MD simulations are marked S1 and S2. The colors of the nearest-neighbor distances (left, purple, blue, green and red lines) reflect the distances between the inserted Phth to the similarly colored phenyl-rings of the bdc linkers (right, purple, blue, green and red colored phenyl rings).

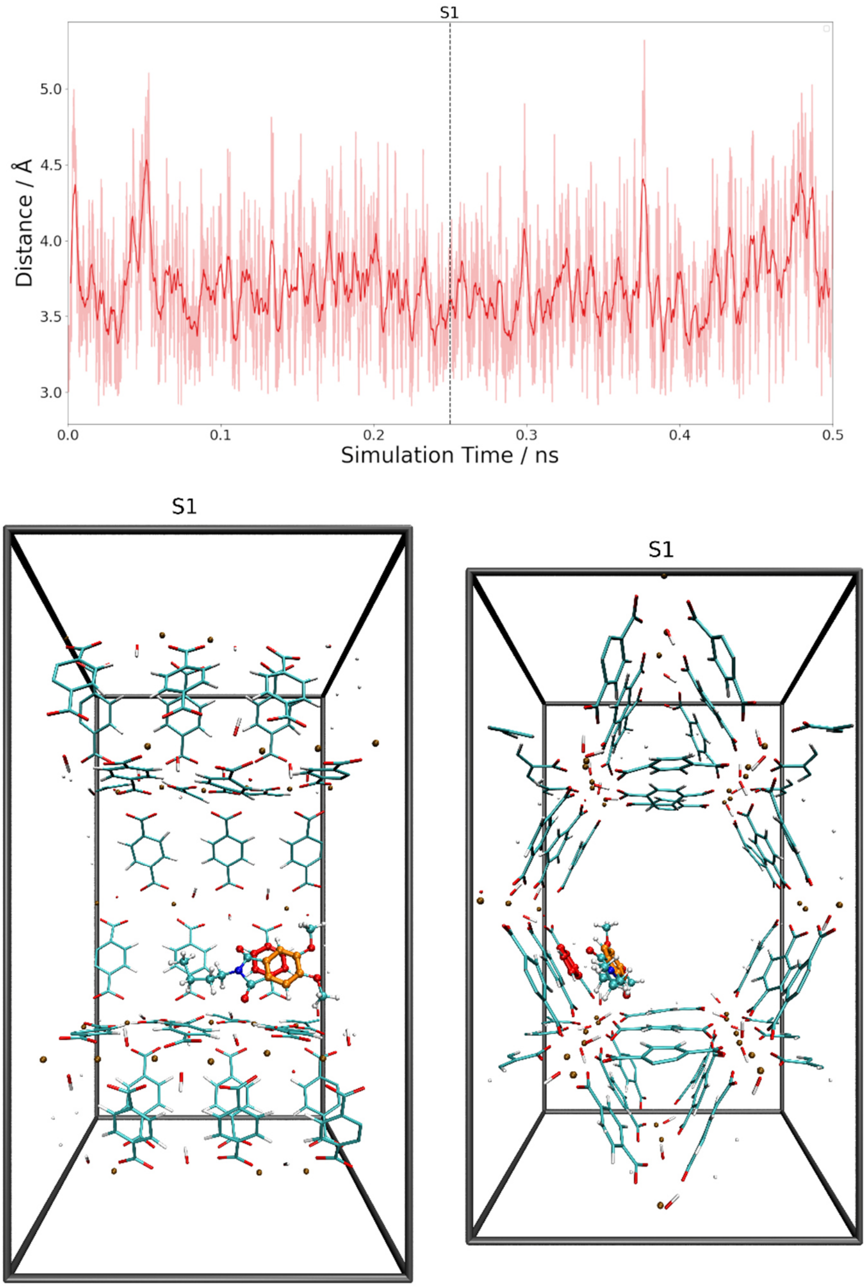

Figure S37. Time evolution of the nearest-neighbor distances between Phth and bdc groups of the $Phth_1$@MIL-68(Ga) host system analyzed based on the centroids of the aromatic units. The latter were determined as an average over the respective carbon atoms. Snapshots along two viewing directions (bottom left: side view on the MOF channel; bottom right: front view on the MOF channel) displaying the key configuration encountered along the DFTB MD simulations are marked S1. The red color of the nearest-neighbor distances (top) reflects the distances between the inserted Phth to the also red colored phenyl-ring of the bdc linker (bottom).

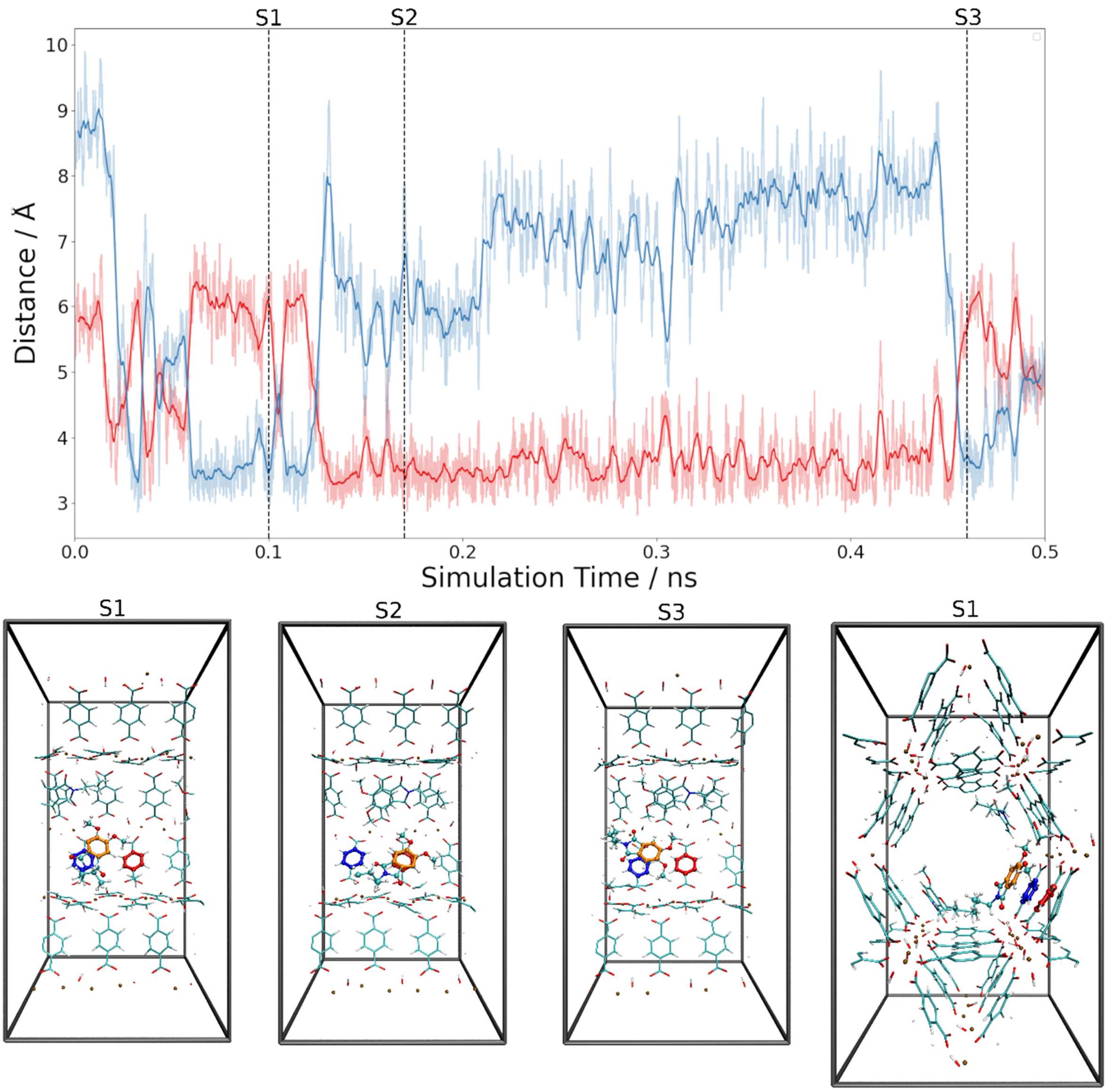

Figure S38. Time evolution of the nearest-neighbor distances between Phth and bdc groups of the $Phth_3$@MIL-68(Ga) host system analyzed based on the centroids of the aromatic units. The latter were determined as an average over the respective carbon atoms. Snapshots displaying the key configurations encountered along the DFTB MD simulations are marked S1 (S1 to S3 (bottom left): side view on the MOF channel; S1 (bottom right): front view on the MOF channel), S2 and S3. The colors of the nearest-neighbor distances (top) reflect the distances between the inserted Phth to the similarly colored phenyl-rings of the bdc linkers (bottom).

Table S1. Solvents used in this investigation (see Fig. S1) with their elution power ε^0 according to Snyder[4] and λ_{max} of 2-butyl-5,6-dimethoxyisoindoline-1,3-dione (= Phth) dissolved in these solvents.

solvent	λ_{max} of $Phth_{(dissolved)}$ / nm	elution power ε^0
methanol	490.5	0.95
ethanol	481	0.88
chloroform	444	0.44
acetone	439.5	0.56
toluene	431	0.29

Table S2. Results of the *Le Bail* fit of high-resolution synchrotron powder diffraction data of Phth@MIL-53(Al) (**4**) compared to the published structural data of MIL-53(Al) *ht*.[3,4]

	Phth@MIL-53(Al) (**4**)	MIL-53(Al) ***ht***
T/K	298	548
Space Group / No.	*Imma* / 74	*Imma* / 74
GOF	3.67	
R_p	0.087	0.137
wR_p	0.1236	0.170
background function	Chebyshev (6 terms)	
GW	4.07(8)	
LY	19(1)	
zero shift	-0.23(3)	
number of data points	5228	
V/Å^3	1445.5(2)	1411.95
a/Å	6.6008(5)	6.6085(9)
b/Å	16.137(1)	16.675(3)
c/Å	13.571(1)	12.813(2)

Composition. For compounds **1** to **10**, XPS measurements were conducted to determine the composition of the Phth@PM hybrid systems (PM: porous material), i.e. the degree of guest loading. The calculations for the fits are listed in Tables S3 (**1-5**) and S4 (**6-10**), the results of these calculations are summarized in Table S5. In Tables S6 and S7 the respective calculations and results are given for the dilution series of Phth@MOF-5 and Phth@MIL-68(Ga). The fits for the XPS measurements of all compounds are presented in Figures S13 to S24. For compound **8**, a significantly higher amount of Phth is found compared to the weights applied in the synthesis. As known from previous studies,[6] the guest molecules within UiO-66 prefer positions close to the $[Zr_6O_4(OH)_4]$ metal-nodes. This leads to shielding effects of the Zr-cations and thus a reduced XPS signal of these cations, which, on the other hand, leads to an overestimation of the guest molecule loading.

Table S3. Calculation of the composition of **1** to **5** *via* XPS.

	**1**	**2**	**3**	**4**	**5**
Nitrogen atoms per formula unit of Phth	1	1	1	1	1
Nitrogen atoms per formula unit of the MOF	0	0	0	0	0
Metal atoms per formula unit of the MOF	4<br>$Zn_4O(bdc)_3$	1<br>In(OH)(bdc)	1<br>Ga(OH)(bdc)	1<br>Al(OH)(bdc)	2<br>$Ga_2(OH)_2(bptc)$
RSF factor for nitrogen	1.77<br>N 1s	1.77<br>N 1s	1.77<br>N 1s	1.77<br>N 1s	1.77<br>N 1s
RSF factor for the respective metal	18.01<br>Zn $2p_{3/2}$	13.23<br>In $3d_{5/2}$	20.47<br>Ga $2p_{3/2}$	0.681<br>Al $2s_{1/2}$	20.47<br>Ga $2p_{3/2}$
Peak area for nitrogen	1494	1074	1411	1026	1238
Peak area for the respective metal	25888	16452	44027	979	33026
Phth : MOF	2.4 : 1	0.5 : 1	0.4 : 1	0.4 : 1	0.9 : 1

Table S4. Calculation of the composition of **6** to **10** *via* XPS (PM: porous material).

	**6**	**7**	**8**	**9**	**10**
Nitrogen atoms per formula unit of Phth	1	1	1	1	1
Nitrogen atoms per formula unit of the PM	0	0	0	4	0
Metal atoms per formula unit of the PM	2<br>$Ga_2(OH)_2$(F-bptc)	2<br>$Ga_2(OH)_2(F_2$-bptc)	6<br>$Zr_6O_4(OH)_4(bdc)_6$	1<br>$ZnC_6H_6N_4$	2 (Al)<br>$(SiO_2)_{5.1}$:Al_2O_3
RSF factor for nitrogen	1.77<br>N 1s	1.77<br>N 1s	1.77<br>N 1s	1.77<br>N 1s	1.77<br>N 1s
RSF factor for the respective metal	20.47<br>Ga $2p_{3/2}$	20.47<br>Ga $2p_{3/2}$	1.54<br>Zr $3d_{5/2}$	18.01<br>Zn $2p_{3/2}$	0.681<br>Al $2s_{1/2}$
Peak area for nitrogen	550	854	215	1189	838
Peak area for the respective metal	13273	17943	862	4168	867
Phth : PM	1 : 1	1.1 : 1	3.5 : 1	0.6 : 1	0.7 : 1

Table S5. Ratio of Phth per formula unit of the PM in **1** to **10** as calculated from the XPS data.

		Phth : PM ratio
$Phth_x$@MOF-5 (**1**)	Phth : $Zn_4O(bdc)_3$	2.4 : 1
$Phth_x$@MIL-68(In) (**2**)	Phth : In(OH)(bdc)	0.5 : 1
$Phth_x$@MIL-68(Ga) (**3**)	Phth : Ga(OH)(bdc)	0.4 : 1
$Phth_x$@MIL-53(Al) (**4**)	Phth : Al(OH)(bdc)	0.4 : 1
$Phth_x$@MFM-300(Ga_2) (**5**)	Phth : $Ga_2(OH)_2$(bptc)	0.9 : 1
$Phth_x$@UoC-2(Ga,1F) (**6**)	Phth : $Ga_2(OH)_2$(F-bptc)	1 : 1
$Phth_x$@UoC-2(Ga,2F) (**7**)	Phth : $Ga_2(OH)_2$(F_2-bptc)	1.1 : 1
$Phth_x$@UiO-66 (**8**)	Phth : $Zr_6O_4(OH)_4(bdc)_6$	3.5 : 1
$Phth_x$@ZIF-8 (**9**)	Phth : $ZnC_6H_6N_4$	0.6 : 1
$Phth_x$@Zeolite Y (**10**)	Phth : $(SiO_2)_{5.1}$:Al_2O_3	0.7 : 1

Table S6. Calculation of the composition of the dilution series of Phth@MOF-5 *via* XPS.

Set guest:host ratio (synthesis)	**0.1:1**	**0.25:1**	**0.5:1**
Nitrogen atoms per formula unit of Phth	1	1	1
Metal atoms per formula unit of MOF-5	4<br>$Zn_4O(bdc)_3$	4<br>$Zn_4O(bdc)_3$	4<br>$Zn_4O(bdc)_3$
RSF factor for nitrogen	1.77<br>N 1s	1.77<br>N 1s	1.77<br>N 1s
RSF factor for Zn	18.01<br>Zn $2p_{3/2}$	18.01<br>Zn $2p_{3/2}$	18.01<br>Zn $2p_{3/2}$
Peak area for nitrogen	135	266	394
Peak area for Zn	48294	52653	36545
Phth : MOF-5	0.1 : 1	0.2 : 1	0.4 : 1

Table S7. Calculation of the composition of the dilution series of Phth@MIL-68(Ga) *via* XPS.

Set guest:host ratio (synthesis)	**0.1:1**	**0.25:1**	**0.5:1**
Nitrogen atoms per formula unit of Phth	1	1	1
Metal atoms per formula unit of MIL-68(Ga)	1<br>Ga(OH)(bdc)	1<br>Ga(OH)(bdc)	1<br>Ga(OH)(bdc)
RSF factor for nitrogen	1.77<br>N 1s	1.77<br>N 1s	1.77<br>N 1s
RSF factor for Ga	20.47<br>Ga $2p_{3/2}$	20.47<br>Ga $2p_{3/2}$	20.47<br>Ga $2p_{3/2}$
Peak area for nitrogen	930	1008	1190
Peak area for Ga	60926	45081	43736
Phth : MIL-68(Ga)	0.2 : 1	0.3 : 1	0.3: 1

References

(1) Schwartz, H. A.; Werker, M.; Tobeck, C.; Christoffels, R.; Schaniel, D.; Olthof, S.; Meerholz, K.; Kopacka, H.; Huppertz, H.; Ruschewitz, U. Novel Photoactive Spirooxazine Based Switch@MOF Composite Materials. *ChemPhotoChem* **2020**, *4* (3), 195–206. https://doi.org/10.1002/cptc.201900193.

(2) Schwartz, H. A.; Olthof, S.; Schaniel, D.; Meerholz, K.; Ruschewitz, U. Solution-Like Behavior of Photoswitchable Spiropyrans Embedded in Metal–Organic Frameworks. *Inorg. Chem.* **2017**, *56* (21), 13100–13110. https://doi.org/10.1021/acs.inorgchem.7b01908.

(3) Coelho, A. A.; Evans, J.; Evans, I.; Kern, A.; Parsons, S. The TOPAS Symbolic Computation System. *Powder Diffr.* **2011**, *26* (S1), S22–S25. https://doi.org/10.1154/1.3661087.

(4) Petrícek, V.; Dušek, M.; Palatinus, L. Crystallographic Computing System JANA2006: General Features. *Zeitschrift fur Krist.* **2014**, *229* (5), 345–352. https://doi.org/10.1515/zkri-2014-1737.

(5) Loiseau, T.; Serre, C.; Huguenard, C.; Fink, G.; Taulelle, F.; Henry, M.; Bataille, T.; Férey, G. A Rationale for the Large Breathing of the Porous Aluminum Terephthalate (MIL-53) Upon Hydration. *Chem. Eur. J.* **2004**, *10* (6), 1373–1382. https://doi.org/10.1002/chem.200305413.

(6) Küssner, K.; Listyarini, R. V.; Rödl, M.; Olthof, S.; Meerholz, K.; Hofer, T. S.; Schwartz, H. A. Tuning Solid-State Switching of the First Dihydroazulene@MOF Hybrid Materials. *Chem. Mater.* **2023**, *35*, 6953–6865. https://doi.org/10.1021/acs.chemmater.3c01224.
